# Supplementary material for: Add-on effect of Chinese herbal medicine in the treatment of mild to moderate COVID-19: A systematic review and meta-analysis
Source: PLoS One. 2021 Aug 20;16(8):e0256429. doi: 10.1371/journal.pone.0256429 (PMC8378756; doi:10.1371/journal.pone.0256429)
Supplement: Attachment — Submitted filename: PONE-D-20-38124_reviewer(COVID-19 popular chineese medicine) reviewed.pdf [file pone.0256429.s002.pdf]

PLOS ONE

Chinese herbal medicine in adults with mild to moderate coronavirus disease 2019(COVID-19): A systematic review and meta-analysis

--Manuscript Draft--

Manuscript Number: PONE-D-20-38124

Article Type: Research Article

Full Title: Chinese herbal medicine in adults with mild to moderate coronavirus disease 2019(COVID-19): A systematic review and meta-analysis

Short Title: A systematic review and meta-analysis

Corresponding Author: Lipeng Shi
Traditional Chinese Medicine Hospital of Dianjiang Chongqing
Chongqing, CHINA

Keywords: Coronavirus disease 2019, Chinese herbal medicine, Systematic review, Meta-analysis, Randomized controlled trials

Abstract:

Introduction

Coronavirus disease 2019 (COVID-19) infected by SARS-CoV-2 has spread all over the world, which is a serious threat to human life and health. In China's experience in fighting COVID-19, traditional Chinese medicine (TCM), especially Chinese herbal medicine (CHM), has played an important role. Human studies reported the beneficial effects of CHM in the treatment of adult patients with mild to moderate COVID-19. Presently there is no systematic evaluation of the clinical efficacy of CHM in adult patients with mild to moderate COVID-19. Therefore, this review was designed to evaluate the efficacy and safety of CHM in the treatment of adult patients with mild to moderate COVID-19.

Methods

RCTs about CHM for mild to moderate COVID-19 were searched in the following eight electronic databases: PubMed, EMBASE, Cochrane Central Register of Controlled trials, the Clinical Trials.gov website, China National Knowledge Infrastructure (CNKI), China Science and Technology Journal Database (VIP), Wanfang Database and China Biology Medicine (CBM) from December 2019 to November 2020. Two reviewers independently searched, selected studies and extracted data according to the eligibility criteria. Cochrane Risk of Bias (ROB) tool was used to assess the methodological quality of the included RCTs. ~~And~~ Revman5.3.0 software was used for statistical analysis.

Results

Twelve eligible RCTs were included with a total sample size of 1393. Our meta-analyses found that compared with the conventional western medicine (CWM) treatment, the effective rate of lung CT [RR=1.26, 95%CI (1.15, 1.38), P < 0.00001], and clinical cure rate [RR=1.26, 95%CI (1.16, 1.38), P < 0.00001] of the CHM treatment were better. Besides, CHM could reduce the rate of conversion to severe cases [RR=0.48, 95%CI (0.32, 0.73), P =0.0005], TCM symptom score of fever [MD=-0.62, 95%CI (-0.79, -0.45), P < 0.00001], the cough cases [RR=1.43, 95%CI (1.16, 1.75), P =0.0006], TCM symptom score of cough[MD=-1.07, 95%CI (-1.29, -0.85), P < 0.00001], TCM symptom score of fatigue[MD=-0.66, 95%CI (-1.05, -0.28), P =0.0007], CRP[MD=-5.46, 95%CI (-8.19, -2.72), P < 0.0001], and improve WBC count[MD=0.38, 95%CI (0.31, 0.44), P < 0.00001], and the above meta-analysis results were robust and reliable through sensitivity analysis.

Conclusion

Chinese herbal medicine is effective and safe in the treatment of adults with mild to moderate COVID-19. And Chinese herbal medicine may be a promising candidate for the treatment of mild to moderate COVID-19.

Order of Authors:
Xuqin Du
Lipeng Shi
Wenfu Cao
Biao Zuo

Powered by Editorial Manager® and ProduXion Manager® from Aries Systems Corporation

	Aimin Zhou
**Additional Information:**	
**Question**	**Response**
**Financial Disclosure**<br><br>Enter a financial disclosure statement that describes the sources of funding for the work included in this submission. Review the submission guidelines for detailed requirements. View published research articles from *PLOS ONE* for specific examples.<br><br>This statement is required for submission and **will appear in the published article** if the submission is accepted. Please make sure it is accurate.<br><br>**Unfunded studies**<br>Enter: *The author(s) received no specific funding for this work.*<br><br>**Funded studies**<br>Enter a statement with the following details:<br>• Initials of the authors who received each award<br>• Grant numbers awarded to each author<br>• The full name of each funder<br>• URL of each funder website<br>• Did the sponsors or funders play any role in the study design, data collection and analysis, decision to publish, or preparation of the manuscript?<br>• **NO** - Include this sentence at the end of your statement: *The funders had no role in study design, data collection and analysis, decision to publish, or preparation of the manuscript.*<br>• **YES** - Specify the role(s) played.<br><br>* typeset	This study was funded by the National Natural Science Foundation of China (No.81573860) and the postdoctoral research project of Chongqing Medical University (No. R11004). The funders had no role in study design, data collection and analysis, decision to publish, or preparation of the manuscript.
**Competing Interests**<br><br>Use the instructions below to enter a competing interest statement for this submission. On behalf of all authors, disclose any competing interests that could be perceived to bias this work—acknowledging all financial support and any other relevant financial or non-financial competing interests.	The authors declare that they have no competing interests.

Powered by Editorial Manager® and ProduXion Manager® from Aries Systems Corporation

This statement **will appear in the published article** if the submission is accepted. Please make sure it is accurate. View published research articles from *PLOS ONE* for specific examples.<br><br>**NO authors have competing interests**<br><br>Enter: *The authors have declared that no competing interests exist.*<br><br>**Authors with competing interests**<br><br>Enter competing interest details beginning with this statement:<br><br>*I have read the journal's policy and the authors of this manuscript have the following competing interests: [insert competing interests here]*<br><br>* typeset	
**Ethics Statement**<br><br>Enter an ethics statement for this submission. This statement is required if the study involved:<br><br>• Human participants<br>• Human specimens or tissue<br>• Vertebrate animals or cephalopods<br>• Vertebrate embryos or tissues<br>• Field research<br><br>Write "N/A" if the submission does not require an ethics statement.<br><br>General guidance is provided below. Consult the submission guidelines for detailed instructions. **Make sure that all information entered here is included in the Methods section of the manuscript.**	Ethical approval and patient consent are not required since this is an overview based on published studies.

Powered by Editorial Manager® and ProduXion Manager® from Aries Systems Corporation

**Format for specific study types**<br><br>**Human Subject Research (involving human participants and/or tissue)**<br>• Give the name of the institutional review board or ethics committee that approved the study<br>• Include the approval number and/or a statement indicating approval of this research<br>• Indicate the form of consent obtained (written/oral) or the reason that consent was not obtained (e.g. the data were analyzed anonymously)<br><br>**Animal Research (involving vertebrate animals, embryos or tissues)**<br>• Provide the name of the Institutional Animal Care and Use Committee (IACUC) or other relevant ethics board that reviewed the study protocol, and indicate whether they approved this research or granted a formal waiver of ethical approval<br>• Include an approval number if one was obtained<br>• If the study involved *non-human primates*, add *additional details* about animal welfare and steps taken to ameliorate suffering<br>• If anesthesia, euthanasia, or any kind of animal sacrifice is part of the study, include briefly which substances and/or methods were applied<br><br>**Field Research**<br><br>Include the following details if this study involves the collection of plant, animal, or other materials from a natural setting:<br>• Field permit number<br>• Name of the institution or relevant body that granted permission	
**Data Availability**<br><br>Authors are required to make all data underlying the findings described fully available, without restriction, and from the time of publication. PLOS allows rare exceptions to address legal and ethical concerns. See the PLOS Data Policy and FAQ for detailed information.	No - some restrictions will apply

Powered by Editorial Manager® and ProduXion Manager® from Aries Systems Corporation

A Data Availability Statement describing where the data can be found is required at submission. Your answers to this question constitute the Data Availability Statement and **will be published in the article**, if accepted.<br><br>**Important:** Stating 'data available on request from the author' is not sufficient. If your data are only available upon request, select 'No' for the first question and explain your exceptional situation in the text box.<br><br>Do the authors confirm that all data underlying the findings described in their manuscript are fully available without restriction?	
**Describe where the data may be found in full sentences. If you are copying our sample text, replace any instances of XXX with the appropriate details.**<br><br>• If the data are **held or will be held in a public repository**, include URLs, accession numbers or DOIs. If this information will only be available after acceptance, indicate this by ticking the box below. For example: *All XXX files are available from the XXX database (accession number(s) XXX, XXX.).*<br>• If the data are all contained **within the manuscript and/or Supporting Information files**, enter the following: *All relevant data are within the manuscript and its Supporting Information files.*<br>• If neither of these applies but you are able to provide **details of access elsewhere**, with or without limitations, please do so. For example:<br><br>*Data cannot be shared publicly because of [XXX]. Data are available from the XXX Institutional Data Access / Ethics Committee (contact via XXX) for researchers who meet the criteria for access to confidential data.*<br><br>*The data underlying the results presented in the study are available from (include the name of the third party*	Data cannot be shared publicly because the article data is from a meta-analysis of the published literature.

Powered by Editorial Manager® and ProduXion Manager® from Aries Systems Corporation

and contact information or URL).<br>• This text is appropriate if the data are owned by a third party and authors do not have permission to share the data.<br><br>* typeset	
Additional data availability information:	

Manuscript

Click here to access/download;Manuscript;Chinese herbal medicine in adults with COVID-19.pdf

Chinese herbal medicine in adults with mild to moderate coronavirus disease 2019(COVID-19): A systematic review and meta-analysis

Xuqin Du[1,2], Lipeng Shi [3,*], Wenfu Cao [1,2,4,*] , Biao Zuo[1,2], Aimin Zhou[3]

[1] College of Traditional Chinese Medicine, Chongqing Medical University, Chongqing 400016, People's Republic of China

[2] Chongqing Key Laboratory of Traditional Chinese Medicine for Prevention and Cure of Metabolic Diseases, Chongqing 400016, People's Republic of China

[3] Department of Cardiovascular Unit, Traditional Chinese medicine hospital Dianjiang Chongqing, Chongqing 408300, People's Republic of China

[4] Department of Chinese Traditional Medicine, The First Affiliated Hospital of Chongqing Medical University, Chongqing 400016, People's Republic of China

* shilipeng0206@163.com； caowenfu9316@163.com

Data Availability Statement: All relevant data are within the manuscript and its Supporting Information files.

Funding： This study was funded by the National Natural Science Foundation of China (No.81573860) and the postdoctoral research project of Chongqing Medical University (No. R11004). The funders had no role in study design, data collection and analysis, decision to publish, or preparation of the manuscript.

Competing interests: The authors declare that they have no competing interests.

ABSTRACT

Introduction

Coronavirus disease 2019 (COVID-19) infected by SARS-CoV-2 has spread all over the world, which is a serious threat to human life and health. In China's experience in fighting COVID-19, traditional Chinese medicine (TCM), especially Chinese herbal medicine (CHM), has played an important role. Human studies reported the beneficial effects of CHM in the treatment of adult patients with mild to moderate COVID-19.

Presently there is no systematic evaluation of the clinical efficacy of CHM in adult patients with mild to moderate COVID-19. Therefore, this review was designed to evaluate the efficacy and safety of CHM in the treatment of adult patients with mild to moderate COVID-19.

Methods

RCTs about CHM for mild to moderate COVID-19 were searched in the following eight electronic databases: PubMed, EMBASE, Cochrane Central Register of Controlled trials, the Clinical Trials.gov website, China National Knowledge Infrastructure (CNKI), China Science and Technology Journal Database (VIP), Wanfang Database and China Biology Medicine (CBM) from December 2019 to November 2020. Two reviewers independently searched, selected studies and extracted data according to the eligibility criteria. Cochrane Risk of Bias (ROB) tool was used to assess the methodological quality of the included RCTs. And Revman5.3.0 software was used for statistical analysis.

Results

Twelve eligible RCTs were included with a total sample size of 1393. Our meta-analyses found that compared with the conventional western medicine (CWM) treatment, the effective rate of lung CT [RR=1.26, 95%CI (1.15, 1.38), $P<0.00001$], and clinical cure rate [RR=1.26, 95%CI (1.16, 1.38), $P<0.00001$] of the CHM treatment were better. Besides, CHM could reduce the rate of conversion to severe cases [RR=0.48, 95%CI (0.32, 0.73), $P=0.0005$], TCM symptom score of fever [MD=-0.62, 95%CI (-0.79, -0.45), $P<0.00001$], the cough cases [RR=1.43, 95%CI (1.16, 1.75), $P=0.0006$], TCM symptom score of cough[MD=-1.07, 95%CI (-1.29, -0.85), $P<0.00001$], TCM symptom score of fatigue[MD=-0.66, 95%CI (-1.05, -0.28), $P=0.0007$], CRP[MD=-5.46, 95%CI (-8.19, -2.72), $P<0.0001$], and improve WBC count[MD=0.38, 95%CI (0.31, 0.44), $P<0.00001$], and the above meta-analysis results were robust and reliable through sensitivity analysis.

Conclusion

Chinese herbal medicine is effective and safe in the treatment of adults with mild to moderate COVID-19. And Chinese herbal medicine may be a promising candidate for the treatment of mild to moderate COVID-19.

Introduction

Coronavirus disease 2019 (COVID-19) is a novel coronavirus "2019-nCoV" causing a clinical syndrome dominated by the acute respiratory tract, with a large-scale epidemic [1-2]. Up to now, the epidemic has been basically brought under control in China, but the situation in many countries is still grim. As of November 21, 2020, 16765323 cases have been confirmed worldwide; 57969680 cases have been cumulatively diagnosed; 1375205 cases have died, and the mortality rate is 2.4%. COVID-19 has developed into a global public health emergency. Therefore, it is an urgent task to control COVID-19 effectively.

In China's experience in fighting COVID-19, traditional Chinese medicine (TCM), especially Chinese herbal medicine (CHM), has played an important role [3]. A large number of epidemiological investigations showed that mild to moderate COVID-19 accounted for the largest proportion [4]. Conventional western medicine (CWM) in the treatment of mild to moderate COVID-19 is mainly antiviral and symptomatic support treatment, so far, no specific drug for the virus has been developed. CHM treatment which is based on syndrome differentiation could effectively alleviate clinical symptoms, reduce the rate of conversion to severe cases, and improve the cure rate [5]. Although there are several reviews of CHM for COVID-19 published [5-7], retrospective studies were included in the review [5-6], and no subgroup analysis of mild to moderate COVID-19 was performed [5-7].

In our review, the RCTs on CHM in the treatment of adult patients with mild to moderate COVID-19 have been searched since the outbreak of the epidemic. ~~And~~ the efficacy and safety of CHM in adults with mild to moderate COVID-19 were objectively evaluated by systematic evaluation and meta-analysis, in order to further provide evidence-based evidence for CHM in the treatment of COVID-19.

Methods

This review was based on the guidelines of Preferred Reporting Items for Systematic Reviews and Meta-Analyses (PRISMA) [8]. Also, the protocol for our review has been registered on the International Prospective Register of Systematic Reviews (PROSPERO) with registration number CRD42020213528.

Eligibility criteria

Types of studies

Only RCTs of CHM for adults with mild to moderate COVID-19 were included in this review. The retrospective study, observational study, studies with data duplication, cross-over RCTs, and laboratory studies were excluded.

Types of participants

Adult patients (aged≥18 years) diagnosed as mild to moderate COVID-19 could be enrolled in this review without the restriction of race or gender.

Types of interventions

Adult patients in the treatment group were treated by a combination of CHM and CWM. The dosage forms of CHM for mild to moderate COVID-19 in this review contained decoction, granule, capsule, and oral liquid. Patients in the control group were treated by CWM. Also, CWM in the treatment group and the control group was identical. Furthermore, RCTs would be excluded if the treatment group was treated with TCM injection, moxibustion, acupuncture, massage, etc.

Types of outcome measures

The primary outcome of this review was lung computed tomography (CT). High-resolution CT was utilized to observe changes in the chest and lung field before and after treatment. The secondary outcomes included the following items: clinical cure rate, viral nucleic acid testing, rate of conversion to severe cases, clinical symptoms (fever, cough, fatigue), inflammatory biomarkers including white blood cell (WBC) count, lymphocyte (LYM) count, LYM percentage, neutrophils (NEU) percentage, C-reactive protein (CRP), and adverse drug reactions (number of adverse effects cases, nausea and vomit, diarrhea, liver damage).

Literature search

RCTs assessing the efficacy and adverse events of CHM for adults with mild to moderate COVID-19 were searched in the following eight electronic databases: PubMed, EMBASE, Cochrane Central Register of Controlled Trials, the Clinical Trials.gov website, China National Knowledge Infrastructure (CNKI), China Science and Technology Journal Database (VIP), Wanfang Database and China Biology Medicine (CBM) from December 2019 to November 2020. Besides, there was no language restriction in our review. And, the search terms included "coronavirus disease 2019", "COVID-19", "novel coronavirus pneumonia", "traditional Chinese medicine", "Chinese herbal medicine", "Chinese herb", "clinical trial", "randomized controlled trial", "randomised controlled trial".

Study selection and data extraction

The study selection and data extraction of our review were conducted independently by two reviewers (Du XQ and Shi LP) according to the eligibility criteria. The following information would be extracted from the included RCTs: basic characteristics of included RCTs (the title of RCTs, first authors' name, publication date, sample size, and methodological quality), participant characteristics (age, gender, number in each group), intervention details (the type of interventions, type of controls, dose, route of oral administration), and outcome measures including primary and secondary outcome measures, as well as adverse events. Any disagreements would be resolved by consultation with a third reviewer (Cao WF).

Assessment of methodological quality

According to the Cochrane Collaboration's tool [9], the methodological quality of the included RCTs was assessed by two reviewers (Du XQ and Shi LP) independently. Seven items of risk of bias (ROB) including adequate sequence generation, concealment of allocation, blinding (patient, investigator and assessor), incomplete outcome data addressed, free of selective reporting, and other biases were evaluated. Each item of ROB was assessed to be low ROB, high ROB, or unclear ROB. Additionally, any disagreements of ROB were resolved by consultation with a third reviewer (Cao WF).

Statistical analysis

Revman5.3.0 software (The Cochrane Collaboration, Copenhagen, Denmark) was used for statistical analysis. The relative risk (RR) was adopted for the dichotomous variables. And the mean difference (MD) or standard mean difference (SMD) were adopted for the continuous variables. Also, we would set the confidence intervals (CIs) as 95%. The heterogeneity test utilized the $\chi 2$ test and the I^2 statistical value. When the $P \geq 0.10$ or $I^2 \leq 50\%$, a fixed-effect model would be adopted to assess the difference, otherwise, a random-effects model would be selected. Besides, we would conduct a subgroup analysis of the primary outcome according to different treatment courses. And the sensitivity analysis would be performed by removing each included RCT of both primary outcome and secondary outcomes in turn. When the number of included RCTs on an outcome measure was larger than ten, a funnel plot analysis would be performed to evaluate the reporting bias. Moreover, $P < 0.05$ was considered as a statistical difference.

Results

Eligible studies

The flow diagram of study selection and identification was showed in Fig. 1. And the characteristics of included RCTs were listed in Table 1. In this review, a total of twelve eligible RCTs were included [10-21]. Among the twelve RCTs [10-21], three were multi-centered trials [13,14,17] and the rest nine were single-centered trials. All twelve RCTs were conducted in mainland China in 2020. One RCT was online published in English [14], and the rest were online reported in Chinese. The sample size of the included RCTs ranged from 45 to 284 (total 1393). All twelve RCTs assessed the effects of oral CHM combined with CWM compared to CWM alone. The name, usage, dosage of western medicine used in the treatment group were identical to the control group. The treatment duration varied from 5 to 15 days. Seven RCTs [11,14-19] described the effective rate of lung CT. Five RCTs [11-12,14,17,21] described the clinical cure rate. Four RCTs [13-14,17,20] described the viral nucleic acid testing. Nine RCTs [11-18,20] described the rate of conversion to severe cases. Clinical symptoms of fever, cough and fatigue was described in seven RCTs [10-12,16-18,20], of which three RCTs [10,16,20] described number of

fever/cough/fatigue reduction cases, and four RCTs [11-12,18-19] described TCM symptom score of fever/cough/fatigue. Inflammatory biomarkers were described in six RCTs [11-12,17-19,21], of which four RCTs [11-12,18-19] described WBC count, four RCTs [11-12,17-18] described LYM count, three RCTs [11-12,19] described LYM percentage, two RCTs [11,17] described NEU percentage, and six RCTs [11-12,17-19,21] described CRP. Adverse effects were described in ten RCTs [10-14,17-21].

Assessment of methodological quality

The methodological quality of the included RCTs was assessed according to the Cochrane handbook criteria. As shown in Fig.2a and Fig.2b, green and "+" indicate "Low risk"; yellow and "?" indicate "Unclear". Detailed information on sequence generation of randomization was described in ten trials (10/12, 83.33%) [10-18,21]. Detailed information on allocation concealment, blinding of the patient, and blinding of the investigator was not described in this review. One RCT reported blinding of the assessor [14]. All included RCTs described incomplete outcome data addressed. And free of selective reporting and other biases of the included RCTs were unclear.

Efficacy and safety assessment

Effective rate of lung CT

Seven RCTs [11,14-19] reported effective rate of lung CT. A significant improvement in lung CT was identified by CHM treatment in this meta-analysis [n=845, RR=1.26, 95%CI (1.15, 1.38), I^2=8%, $P<0.00001$] (Fig. 3).

Clinical cure rate

Five RCTs evaluated the effects of CHM on clinical cure rate [11-12,14,17,21]. CHM exhibited a significant improvement on clinical cure rate [n=821, RR=1.26, 95%CI (1.16, 1.38), I^2=0%, $P<0.00001$] (Fig. 4).

Viral nucleic acid testing

Viral nucleic acid testing was reported in four RCTs [13-14,17,20]. Compared with CWM, no statistical difference on viral nucleic acid testing was identified [n=581, RR=1.09, 95%CI (0.98, 1.21), I^2=57%, P=0.13] (Fig. 5).

Rate of conversion to severe cases

Rate of conversion to severe cases was reported in nine RCTs [11-18,20]. CHM significantly reduced the rate of conversion to severe cases [n=1121, RR=0.48, 95%CI (0.32, 0.73), I^2 = 0%, P=0.0005] (Fig. 6).

Clinical symptoms of fever, cough and fatigue

Clinical symptoms of fever, cough and fatigue was reported in seven RCTs [10-12,16-18,20]. Among them, three RCTs [10,16,20] reported number of fever/cough/fatigue reduction cases, and four RCTs [11-12,17-18] reported TCM symptom score of fever/cough/fatigue.

Meta-analysis revealed no statistical difference on the number of fever reduction cases between CHM and CWM [n=205, RR=1.14, 95%CI (0.58, 2.25), I^2=95%, P=0.70] (Fig.7a). TCM symptom score of fever is significantly reduced by CHM [n=482, MD=-0.62, 95%CI (-0.79, -0.45), I^2=79%, $P<$0.00001] (Fig.7b).

CHM significantly reduced the cough cases [n=205, RR=1.43, 95%CI (1.16, 1.75), I^2 = 0%, P=0.0006] (Fig.7c); as well as a significant reduction in TCM symptom score of cough was identified by CHM [n=482, MD=-1.07, 95%CI (-1.29, -0.85), I^2=84%, $P<$0.00001] (Fig.7d).

It has been identified that fatigue cases is reduced by CHM[n=205, RR=1.23, 95%CI (1.03, 1.47), I^2=28%, P=0.02] (Fig.7e); also a significant reduction in TCM symptom score of fatigue by CHM [n=482, MD=-0.66, 95%CI (-1.05, -0.28), I^2=98%, P=0.0007] (Fig.7f).

Inflammatory biomarkers

Inflammatory biomarkers were reported in six RCTs [11-12,17-19,21], of which four RCTs [11-12,18-19] reported WBC count, four RCTs [11-12,17-18] reported LYM count, three RCTs [11-12,19] reported LYM percentage, two RCTs [11,17] reported NEU percentage, and six RCTs [11-12,17-19,21] reported CRP.

Meta-analysis revealed a significant improvement on WBC count by CHM [n=478, MD=0.38, 95%CI (0.31, 0.44), I^2=5%, $P <$ 0.00001] (Fig.8a); a significant improvement on LYM count by CHM [n=482, MD=0.26, 95%CI (0.05, 0.47), I^2=97%, P=0.01] (Fig.8b); a significant improvement on LYM percentage by CHM

[n=183, MD=6.65, 95%CI (3.36, 9.94), I^2=93%, $P<0.0001$] (Fig.8c); a significant reduction in NEU percentage by CHM [n=114, MD=-4.56, 95%CI (-5.76, -3.36), I^2=0%, $P<0.00001$] (Fig.8d); a significant reduction in CRP by CHM [n=631, MD=-5.46, 95%CI (-8.19, -2.72), I^2=96%, $P<0.0001$] (Fig.8e).

Adverse effects

In this review, adverse effects were reported in ten RCTs [10-14,17-21], and the rest two RCTs [15-16] did not describe drug adverse effects. Among them, no adverse effect was identified in both treatment and control groups [11-12,17-19]. Adverse effects in the rest five RCTs included gastrointestinal reactions (diarrhea, poor appetite, nausea, vomiting), headache, and abnormal liver function [10,13-14,20-21]. All reported adverse reactions were mild in the treatment and control groups, and were tolerable or alleviated after withdrawal.

Compared with CWM, there was no statistical difference in the number of adverse effects cases [n=759, RR=1.13, 95%CI (0.45, 2.83), I^2=63%, P=0.79] (Fig.9a); no statistical difference in the number of nausea and vomiting cases [n=388, RR=1.09, 95%CI (0.49, 2.41), I^2=0%, P=0.83] (Fig.9b); no statistical difference in the number of diarrhea cases [n=759, RR=1.72, 95%CI (0.34, 8.67), I^2=70%, P=0.51] (Fig.9c); no statistical difference in the number of abnormal liver function cases [n=388, RR=0.41, 95%CI (0.05, 3.69), I^2=78%, P=0.43] (Fig.9d). Additionally, the poor appetite and headache were reported in one RCT [14], and no statistical difference was identified between CHM and CWM.

Subgroup analysis of the primary outcomes

Subgroup analysis of the primary outcomes was shown in Fig.2. Subgroup analysis revealed an improvement on lung CT of 7days treatment duration by CHM [n=845, RR=1.18, 95%CI (1.02, 1.36), I^2 =44%, P=0.03] (Fig.3); a significant improvement on lung CT of 10 to 14 days treatment duration by CHM [n=845, RR=1.34, 95%CI (1.19, 1.50), I^2 = 0%, $P<0.00001$] (Fig. 3).

Sensitivity analysis

Sensitivity analysis revealed that there was a small change in the effect amount, and was a significant difference in effective rate of lung CT, clinical cure rate, rate of

conversion to severe cases, TCM symptom score of fever, number of cough reduction cases, TCM symptom score of cough, TCM symptom score of fatigue, WBC count, and CRP, which indicated the above meta-analysis results to be robust and reliable.

Publication bias

As the number of RCTs in any comparative outcome measure was less than ten, we did not assess the publication bias.

Discussion

Since December 2019, COVID-19 infected by SARS-CoV-2 has spread all over the world, which is a serious threat to human life and health. COVID-19 is highly contagious and has a long incubation period. It is generally susceptible to human infection and can be transmitted to each other [22]. In addition, severe cases are more likely to have serious complications, such as shock, ARDS, arrhythmia and acute heart injury[23-24], which significantly increases the difficulty and cost of treatment. Therefore, it is of great significance to prevent COVID-19 from developing from mild and moderate to severe. Since the outbreak of COVID-19, CHM has been widely used to control COVID-19 in China. To our knowledge, this review would be the first one, which objectively assesses the efficacy and safety of CHM in the treatment of adults with mild to moderate COVID-19.

This review systematically evaluated the efficacy and safety of CHM in the treatment of adult patients with mild to moderate COVID-19. After a comprehensive search of eight databases, twelve RCTs were enrolled in our review. The meta-analysis results showed that compared with CWM, CHM combined with CWM has a better therapeutic effect. According to the theory of traditional Chinese medicine, COVID-19 belongs to epidemic disease. The pathogenesis of mild to moderate COVID-19 is dampness-heat or cold-dampness obstructing the lung. Therefore, the treatment principles of heat-clearing, eliminating dampness, resolving phlegm and dispersing cold are widely used. In the included studies, nine different oral CHM were used, including Jinhua Qinggan granule [10], Toujie Quwen granule [11-12], Jinyinhua oral liquid [13,20], Lianhua Qingwen capsule (granule) [14,18], Maxing Xuanfei Jiedu Decoction [15], Lianhua Qingke granule [16], Reyanning mixture [17],

Jiawei Dayuan Decoction [19], diammonium glycyrrhizinate [21]. Among the nine oral CHM, the most frequently used Chinese medicine was honeysuckle, which was used in seven trials (7/12, 58.33%) [10-14,18,20], followed by forsythia (6/12, 50.00%) [10-12,14,16,18], and ephedra (6/12, 50.00%) [10,14-16,18-19]. Honeysuckle and forsythia have the function of clearing heat-toxicity and dispersing wind-heat in the theory of TCM. Honeysuckle polysaccharide is an active component of honeysuckle, which can regulate non-specific immunity [25], inhibit the expression of inflammatory factors TNF-α and IL-1β [26], and inhibit a variety of viruses [27]. Phillyrin is an active component of forsythia, which has antiviral and anti-inflammatory activities [28-29]. Ephedra has the function of dissipating cold and diffusing the lung to calm panting in TCM theory. Ephedrine is an active component of ephedra, which can increase the production of anti-inflammatory cytokines IL-10, reduce the production of pro-inflammatory cytokines TNF-α and IL-12[30], and play an antiviral role by inhibiting viral replication [31].

In this review, it was found that CHM was effective and safe for adult patients with mild to moderate COVID-19. CHM could not only improve the lung CT, clinical cure rate and the main clinical symptoms (fever, cough and fatigue), but also reduce the rate of conversion to severe cases, and regulate the inflammatory response, with fewer adverse reactions. Therefore, CHM may be a promising candidate for the treatment of adults with mild to moderate COVID-19.

Limitations

The limitations of this review were as follows. First of all, most of the included RCTs had deficiencies in methodology design, including hidden allocation and inadequate reporting of blind methods. Secondly, the composition, dosage and frequency of CHM were different in the treatment group. Thirdly, the multicenter RCTs were lacking. In addition, the duration of the included studies ranged from 5 to 15 days. Therefore, it may be necessary to design more high-quality RCTs with a multicenter, large sample and longer follow-up to better observe the efficacy and possible adverse reactions of CHM in the treatment of adults with mild to moderate COVID-19.

Conclusion

CHM is effective and safe in the treatment of adults with mild to moderate COVID-19. It can improve the clinical cure rate, main clinical symptoms, imaging and laboratory indexes, and reduce the rate of conversion to severe cases. However, limited to the fact that COVID-19 is a sudden disease, it is difficult to carry out double-blind clinical trials, which results in the insufficient methodology of the existing-related trials. Therefore, more high-quality trials are needed to evaluate the efficacy and safety of CHM in the treatment of adults with mild to moderate COVID-19 in the future.

Supporting information

S1 Checklist. PRISMA 2009 checklist.

(DOC)

Author Contributions

Conceptualization: Lipeng Shi, Wenfu Cao.

Data curation: Xuqin Du, Lipeng Shi.

Formal analysis: Xuqin Du.

Funding acquisition: Xuqin Du.

Investigation: Xuqin Du, Lipeng Shi.

Methodology: Xuqin Du, Lipeng Shi.

Software: Xuqin Du.

Supervision: Wenfu Cao.

Validation: Biao Zuo, Aimin Zhou.

Writing – original draft: Xuqin Du.

Writing – review & editing: Xuqin Du, Lipeng Shi, Wenfu Cao, Biao Zuo, Aimin Zhou.

References

1. Huang C, Wang Y, Li X, et al. Clinical features of patients infected with 2019 novel coronavirus in Wuhan, China[J]. Lancet. 2020, 395(10223): 497-506.

2. Ralph R, Lew J, Zeng T, et al. 2019-nCoV (Wuhan virus), a novel Coronavirus: human-to-human transmission, travel-related cases, and vaccine readiness [J]. J Infect Dev Ctries, 2020, 14(1): 3-17.
3. Ren JL, Zhang AH, Wang XJ. Traditional Chinese medicine for COVID-19 treatment[J]. Pharmacol Res, 2020, 155:104743.
4. Wu ZY, McGoogan JM. Characteristics of and important lessons from the coronavirus disease 2019(COVID-19) outbreak in China[J]. J Am Med Assoc, 2020, 323(13): 1239-1242.
5. Xiong X, Wang P, Su K, et al. Chinese herbal medicine for coronavirus disease 2019: A systematic review and meta-analysis[J]. Pharmacol Res, 2020, 160:105056.
6. Zeng M, Li L, Wu Z. Traditional Chinese medicine Lianhua Qingwen treating corona virus disease 2019(COVID-19): Meta-analysis of randomized controlled trials[J]. PLoS One, 2020, 15(9):e0238828.
7. Liu M, Gao Y, Yuan Y, et al. Efficacy and safety of integrated traditional Chinese and western medicine for corona virus disease 2019 (COVID-19): a systematic review and meta-analysis[J]. Pharmacol Res, 2020, 158:104896.
8. Moher D, Liberati A, Tetzlaff J, et al. Preferred reporting items for systematic reviews and meta-analyses: the PRISMA statement[J]. Ann Intern Med, 2009,151(4):264-W64.
9. Higgins JPT, Green S. Cochrane Reviewers' Handbook 5.3.0 [updated March 2014], Review Manager (RevMan) [Computer program]. Version 5.3.0. Available from www.cochrane-handbook.org
10. Duan C, Xia GW, Zheng CJ, et al. Clinical observation on Jinhua Qinggan granule combined with conventional western medicine therapy in treating mild cases of coronavirus disease 2019 [J]. J Tradit Chin Med,2020,61(17):1473-1477.
11. Fu XX, Lin LP, Tan XX. Clinical observation on effect of Toujie Quwen granules in treatment of COVID-19 [J]. Chin J Exp Tradit Med Formul,2020,26(12):44-48.
12. Fu XX, Lin LP, Tan XX. Clinical study on 37 case of COVID- 19 treated with integrated traditional Chinese and western medicine [J]. Tradit Chin Drug Res

Clin Pharmacol,2020,31(05):600-604.

13. Hu F, Guo AH, Huang L, et al. Multi-center clinical observation of Jinyinhua oral liquid combined with western medicine in treatment of moderate COVID-19 [J/OL]. J Tradit Chin Med:1-6[2020-11-4]. http://kns.cnki.net/kcms/detail/11.2166.R.20200819.0837.002.html.
14. Hu K, Guan WJ, Bi Y, et al. Efficacy and safety of Lianhuaqingwen capsules, a repurposed Chinese herb, in patients with coronavirus disease 2019: A multicenter, prospective, randomized controlled trial [published online ahead of print, 2020 May 16]. Phytomedicine, 2020,153242. https://doi.org/10.1016/j.phymed.2020.153242.
15. Qiu M, Li Q T, Zhu DP, et al. Efficacy observation of Maxing Xuanfei Jiedu decoction on moderate COVID-19 patients[J]. J Emerg Tradit Chin Med,2020,29(07):1129-1130+1132.
16. Sun HM, Xu F, Zhang L, et al. Study on clinical efficacy of Lianhua Qingke granule in treatment of mild and ordinary COVID-19 [J]. Chin J Exp Tradit Med Formul,2020,26(14):29-34.
17. Yang MB, Dang SS, Haung S, et al. Multi-center clinical observation of Reyanning mixture in treatment of COVID-19 [J]. Chin J Exp Tradit Med Formul,2020,26(14):7-12.
18. Yu P, Li YZ, Wan SB, et al. Clinical efficacy of Lianhua Qingwen granule combined with abidol in treating coronavirus disease 2019 [J]. Chin Pharm J,2020,55(12):1042-1045.
19. Zhang CT, Yang Y, You FM, et al. Clinical study on COVID-19 from the perspective of "Yidujiashi" theory [J]. Pharmacol Clin Chin Materia Medica,2020,36(02):43-45.
20. Zhang YL, Lei L, Xu Y, et al. Clinical efficacy of Jinyinhua oral liquid in the treatment of 80 patients with coronavirus disease 2019 [J]. Chin Pharm,2020,29(09):23-26.
21. Zhou WM, Zhao FM, Li BL, et al. Clinical value of diammonium glycyrrhizinate in treatment of COVID-19 [J]. Chin J Virol,2020,36(02):160-164.

22. Li Q, Guan X, Wu P, et al. Early Transmission Dynamics in Wuhan, China, of Novel Coronavirus-Infected Pneumonia [J]. N Engl J Med, 2020,382(13):1199-1207.
23. Chen N, Zhou M, Dong X, et al. Epidemiological and clinical characteristics of 99 cases of 2019 novel coronavirus pneumonia in Wuhan, China: a descriptive study [J]. Lancet, 2020,395(10223):507-513.
24. Wang DW, Hu B, Hu C, et al. Clinical characteristics of 138 hospitalized patients with 2019 novel coronavirus-infected pneumonia in Wuhan, China [J]. JAMA, 2020, 323(11): 1061-1069.
25. Zhou XN, Dong Q, Kan XZ, et al. Immunomodulatory activity of a novel polysaccharide from Lonicera japonica immunosuppressed mice induced by cyclophosphamide [J]. PloS one,2018,13(10):1-15.
26. Bai XY, Chai YW, Shi WL, et al. Lonicera japonica poly-saccharides attenuate ovalbumin-induced allergic rhinitis by regulation of Th17 cells in BALB/c mice[J]. J Functional Foods,2020,65:103758.
27. Wang J, Hou L, Chen YQ, et al. Extraction and purification of the Lonicera japonica polysaccharide and its antiviral activity in vitro[J]. Chin J Hosp Pharm, 2018,38(8): 810-812.
28. Qu XY, Li QJ, Zhang HM, et al. Protective effects of phillyrin against influenza a virus in vivo[J]. Arch Pharm Res, 2016, 39(7):998-1005.
29. Jiang Q, Chen J, Long X, et al. Phillyrin protects mice from traumatic brain injury by inhibiting the inflammation of microglia via PPARγ signaling pathway[J]. Int Immunopharmacol, 2020,79:106083.
30. He W, Ma J, Chen Y, et al. Ephedrine hydrochloride protects mice from staphylococcus aureus-induced peritonitis[J]. Am J Transl Res, 2018,10(3):670-683.
31. Wei W, Du H, Shao C, et al. Screening of antiviral components of Ma Huang Tang and investigation on the ephedra alkaloids efficacy on influenza virus type A[J]. Front Pharmacol, 2019,10:961.

The figure legend:

Fig. 1. Flow diagram of study selection and identification.

Fig.2.Assessment of methodological quality.

Fig.3 Effective rate of lung CT.

Fig.4 Clinical cure rate.

Fig.5 Viral nucleic acid testing.

Fig.6 Rate of conversion to severe cases.

Fig.7. Clinical symptoms of fever, cough and fatigue.

Fig.8. Inflammatory biomarkers.

Fig.9. Adverse effects.

The table:

Table 1 Characteristics of included RCTs.

Figure

Click here to access/download;Figure;Fig. 1. Flow diagram of study selection and identification..pdf

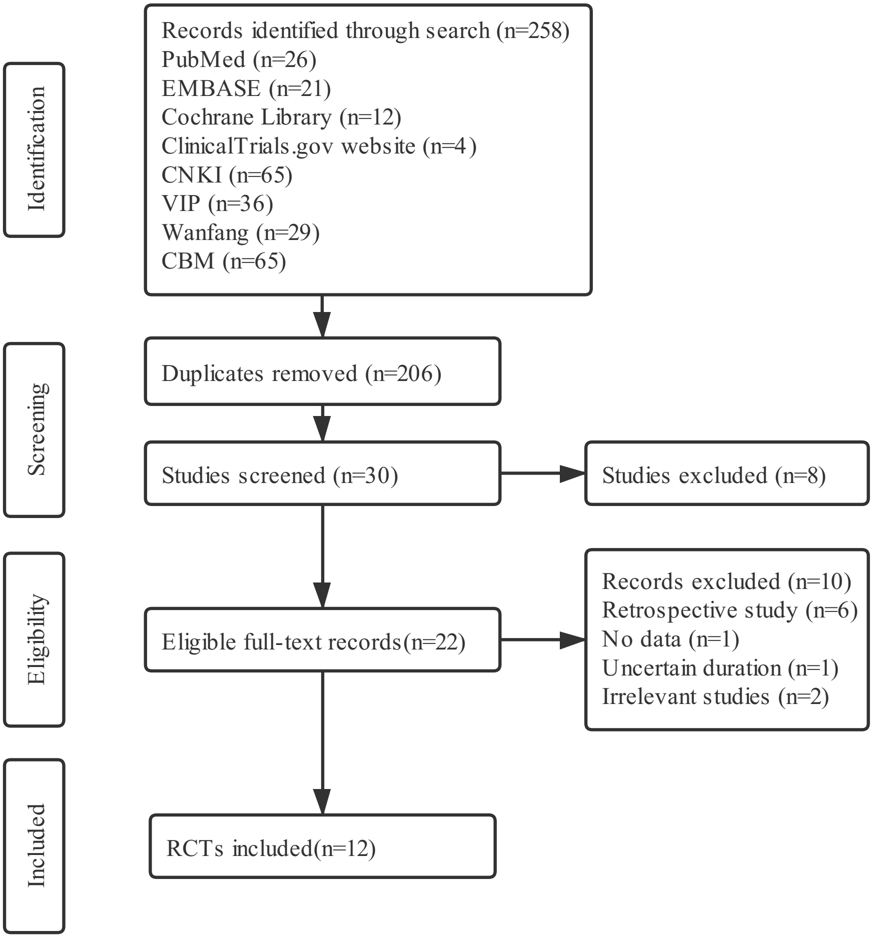

Fig. 1. Flow diagram of study selection and identification.

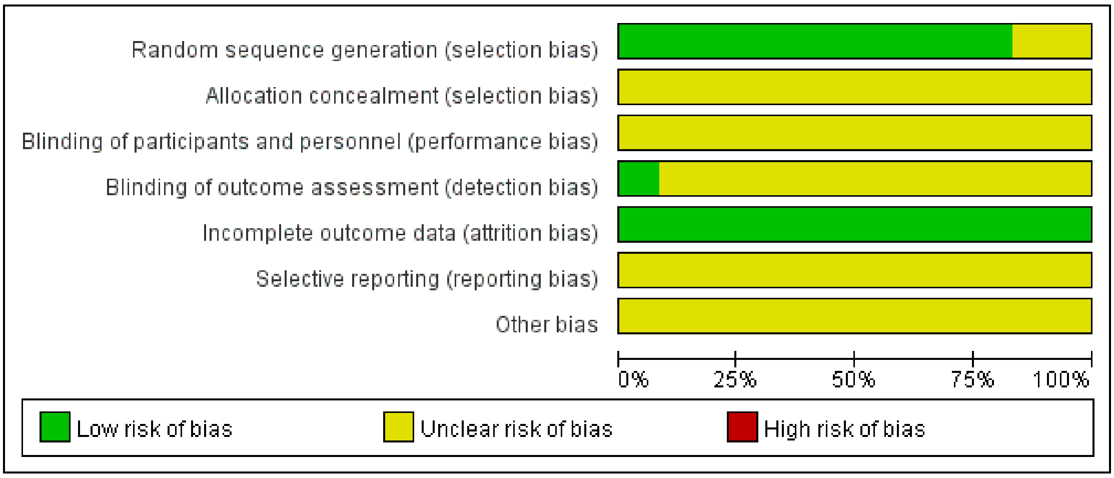

Fig.2a Risk of bias graph.

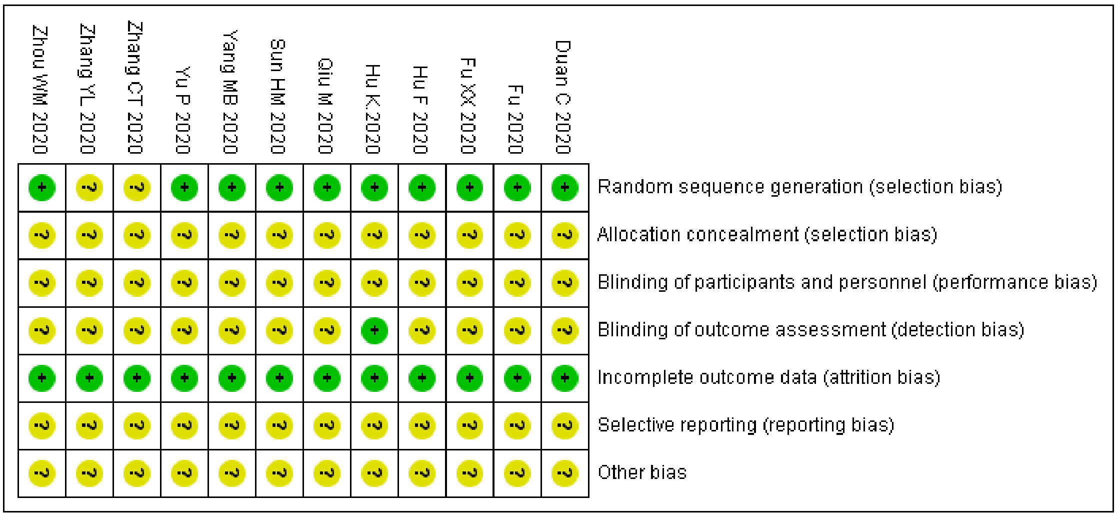

Fig.2b Risk of bias summary.

Fig.2. Assessment of methodological quality. 2a Risk of bias graph. 2b Risk of bias summary.

Figure

Click here to access/download;Figure;Fig.3. Effective rate of lung CT.pdf

Study or Subgroup	Experimental Events	Experimental Total	Control Events	Control Total	Weight	Risk Ratio M-H, Fixed, 95% CI
**1.1.1 Treatment duration of 7 days**						
Yang MB 2020	23	26	17	23	7.3%	1.20 [0.90, 1.58]
Yu P 2020	102	147	93	148	37.3%	1.10 [0.94, 1.30]
Zhang CT 2020	12	22	5	23	2.0%	2.51 [1.06, 5.95]
**Subtotal (95% CI)**		**195**		**194**	**46.5%**	**1.18 [1.02, 1.36]**
Total events	137		115			
Heterogeneity: Chi² = 3.55, df = 2 (P = 0.17); I² = 44%						
Test for overall effect: Z = 2.22 (P = 0.03)						
**1.1.2 Treatment duration of 10 to 14 days**						
Fu 2020	26	32	20	33	7.9%	1.34 [0.97, 1.85]
Hu K.2020	119	142	91	142	36.6%	1.31 [1.13, 1.51]
Qiu M 2020	5	25	2	25	0.8%	2.50 [0.53, 11.70]
Sun HM 2020	31	32	18	25	8.1%	1.35 [1.05, 1.73]
**Subtotal (95% CI)**		**231**		**225**	**53.5%**	**1.34 [1.19, 1.50]**
Total events	181		131			
Heterogeneity: Chi² = 0.72, df = 3 (P = 0.87); I² = 0%						
Test for overall effect: Z = 4.78 (P < 0.00001)						
**Total (95% CI)**		**426**		**419**	**100.0%**	**1.26 [1.15, 1.38]**
Total events	318		246			
Heterogeneity: Chi² = 6.50, df = 6 (P = 0.37); I² = 8%						
Test for overall effect: Z = 4.96 (P < 0.00001)						
Test for subgroup differences: Chi² = 1.74, df = 1 (P = 0.19), I² = 42.4%						

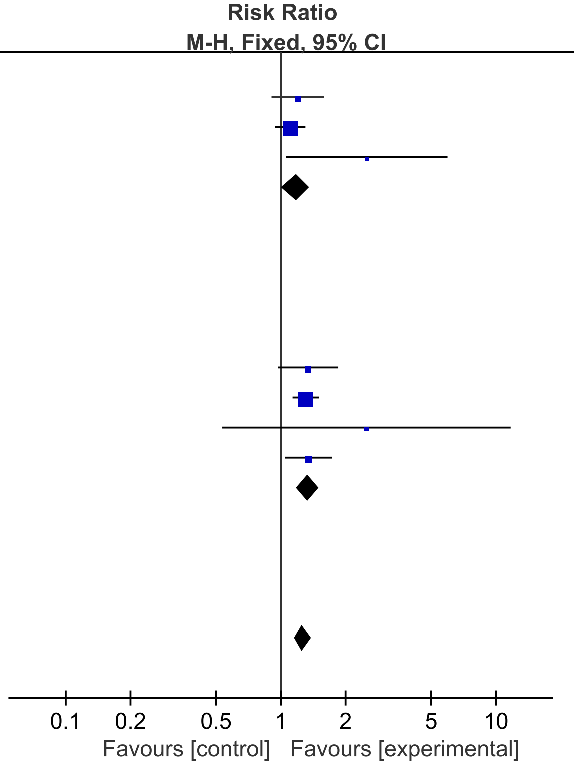

Fig.3 Effective rate of lung CT

Figure
Click here to access/download;Figure;Fig.4. Clinical cure rate.pdf

	Experimental		Control			Risk Ratio
Study or Subgroup	Events	Total	Events	Total	Weight	M-H, Fixed, 95% CI
Fu 2020	30	32	23	33	8.8%	1.35 [1.06, 1.71]
Fu XX 2020	33	37	25	36	9.8%	1.28 [1.01, 1.64]
Hu K.2020	119	147	96	148	37.0%	1.25 [1.08, 1.44]
Yu P 2020	112	142	94	142	36.3%	1.19 [1.03, 1.38]
Zhou WM 2020	32	52	21	52	8.1%	1.52 [1.03, 2.26]
**Total (95% CI)**		**410**		**411**	**100.0%**	**1.26 [1.16, 1.38]**
Total events	326		259			

Heterogeneity: Chi² = 1.79, df = 4 (P = 0.77); I² = 0%
Test for overall effect: Z = 5.21 (P < 0.00001)

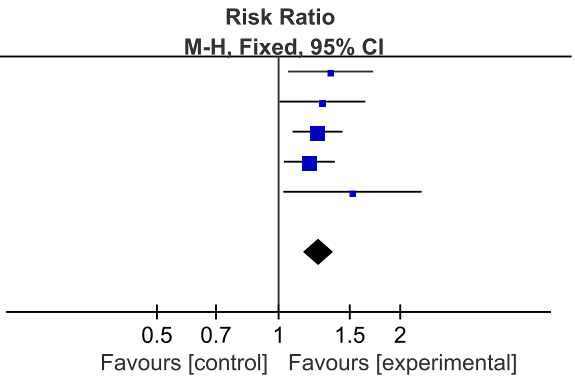

Fig.4 Clinical cure rate

Figure
Click here to access/download;Figure;Fig.5. Viral nucleic acid testing.pdf

Study or Subgroup	Experimental Events	Experimental Total	Control Events	Control Total	Weight	Risk Ratio M-H, Random, 95% CI
Hu F 2020	53	57	62	71	31.4%	1.06 [0.95, 1.19]
Hu K.2020	109	142	101	142	27.0%	1.08 [0.94, 1.24]
Yang MB 2020	25	26	14	23	8.6%	1.58 [1.13, 2.21]
Zhang YL 2020	75	80	37	40	33.1%	1.01 [0.91, 1.13]
**Total (95% CI)**		**305**		**276**	**100.0%**	**1.09 [0.98, 1.21]**
Total events	262		214			

Heterogeneity: Tau² = 0.01; Chi² = 6.90, df = 3 (P = 0.08); I² = 57%
Test for overall effect: Z = 1.51 (P = 0.13)

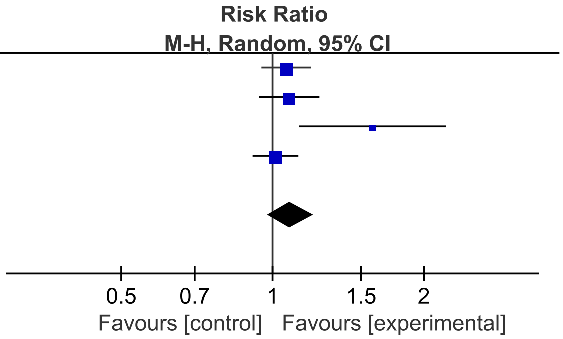

Fig. 5 Viral nucleic acid testing

Figure

Click here to access/download;Figure;Fig.6. Rate of conversion to severe cases.pdf

Study or Subgroup	Experimental Events	Experimental Total	Control Events	Control Total	Weight	Risk Ratio M-H, Fixed, 95% CI
Fu 2020	1	32	3	33	4.9%	0.34 [0.04, 3.13]
Fu XX 2020	2	37	4	36	6.8%	0.49 [0.09, 2.49]
Hu F 2020	0	57	3	71	5.2%	0.18 [0.01, 3.36]
Hu K.2020	3	142	6	142	10.0%	0.50 [0.13, 1.96]
Qiu M 2020	0	25	1	25	2.5%	0.33 [0.01, 7.81]
Sun HM 2020	0	32	2	25	4.7%	0.16 [0.01, 3.14]
Yang MB 2020	0	26	0	23		Not estimable
Yu P 2020	21	147	35	148	58.2%	0.60 [0.37, 0.99]
Zhang YL 2020	0	80	3	40	7.8%	0.07 [0.00, 1.37]
**Total (95% CI)**		**578**		**543**	**100.0%**	**0.48 [0.32, 0.73]**
Total events	27		57			

Heterogeneity: Chi² = 3.54, df = 7 (P = 0.83); I^2 = 0%

Test for overall effect: Z = 3.48 (P = 0.0005)

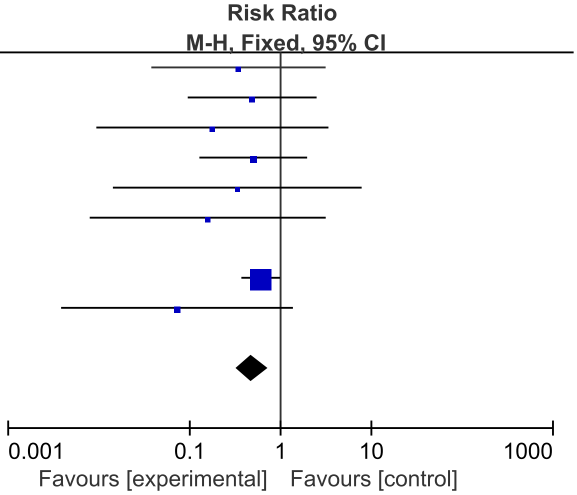

Fig. 6 Rate of conversion to severe cases

Figure

Click here to access/download;Figure;Fig.7. Clinical symptoms of fever, cough and fatigue..pdf

Study or Subgroup	Experimental Events	Experimental Total	Control Events	Control Total	Weight	Risk Ratio M-H, Random, 95% CI
Duan C 2020	53	66	17	32	32.5%	1.51 [1.07, 2.14]
Sun HM 2020	4	4	5	5	32.0%	1.00 [0.68, 1.46]
Zhang YL 2020	68	68	30	30	35.5%	1.00 [0.95, 1.05]
**Total (95% CI)**		**138**		**67**	**100.0%**	**1.14 [0.58, 2.25]**
Total events	125		52			

Heterogeneity: Tau² = 0.34; Chi² = 40.51, df = 2 (P < 0.00001); I² = 95%
Test for overall effect: Z = 0.39 (P = 0.70)

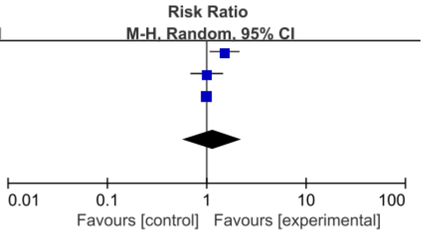

Fig.7a Number of fever reduction cases

Study or Subgroup	Experimental Mean	Experimental SD	Experimental Total	Control Mean	Control SD	Control Total	Weight	Mean Difference IV, Random, 95% CI
Fu 2020	0.46	0.1	32	1.26	0.42	33	33.1%	-0.80 [-0.95, -0.65]
Fu XX 2020	0.56	0.3	37	1.06	0.62	36	24.9%	-0.50 [-0.72, -0.28]
Yang MB 2020	0	0	26	0.22	0.6	23		Not estimable
Yu P 2020	0.56	0.14	147	1.12	0.32	148	42.0%	-0.56 [-0.62, -0.50]
**Total (95% CI)**			**242**			**240**	**100.0%**	**-0.62 [-0.79, -0.45]**

Heterogeneity: Tau² = 0.02; Chi² = 9.48, df = 2 (P = 0.009); I² = 79%
Test for overall effect: Z = 7.20 (P < 0.00001)

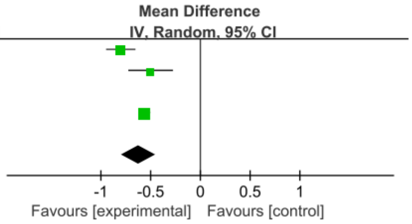

Fig.7b TCM symptom score of fever

Study or Subgroup	Experimental Events	Experimental Total	Control Events	Control Total	Weight	Risk Ratio M-H, Fixed, 95% CI
Duan C 2020	41	62	12	28	28.7%	1.54 [0.97, 2.45]
Sun HM 2020	29	32	16	25	31.2%	1.42 [1.03, 1.94]
Zhang YL 2020	56	62	16	24	40.1%	1.35 [1.01, 1.82]
**Total (95% CI)**		**156**		**77**	**100.0%**	**1.43 [1.16, 1.75]**
Total events	126		44			

Heterogeneity: Chi² = 0.23, df = 2 (P = 0.89); I² = 0%
Test for overall effect: Z = 3.41 (P = 0.0006)

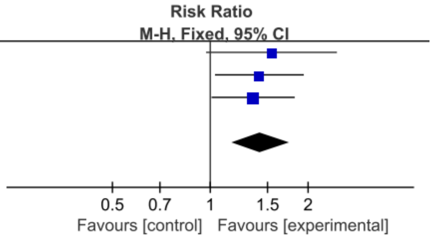

Fig.7c Number of cough reduction cases

Study or Subgroup	Experimental Mean	Experimental SD	Experimental Total	Control Mean	Control SD	Control Total	Weight	Mean Difference IV, Random, 95% CI
Fu 2020	1.45	0.36	32	2.8	0.35	33	26.0%	-1.35 [-1.52, -1.18]
Fu XX 2020	1.55	0.37	37	2.58	0.38	36	26.1%	-1.03 [-1.20, -0.86]
Yang MB 2020	0.23	0.43	26	0.83	0.65	23	19.0%	-0.60 [-0.91, -0.29]
Yu P 2020	1.39	0.42	147	2.56	0.51	148	28.9%	-1.17 [-1.28, -1.06]
**Total (95% CI)**			**242**			**240**	**100.0%**	**-1.07 [-1.29, -0.85]**

Heterogeneity: Tau² = 0.04; Chi² = 18.99, df = 3 (P = 0.0003); I² = 84%
Test for overall effect: Z = 9.63 (P < 0.00001)

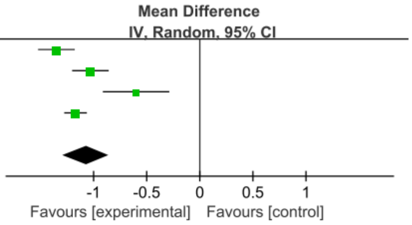

Fig.7d TCM symptom score of cough

Study or Subgroup	Experimental Events	Experimental Total	Control Events	Control Total	Weight	Risk Ratio M-H, Fixed, 95% CI
Duan C 2020	45	58	14	26	34.8%	1.44 [0.98, 2.11]
Sun HM 2020	14	14	8	10	17.6%	1.25 [0.90, 1.75]
Zhang YL 2020	53	58	18	21	47.6%	1.07 [0.88, 1.29]
**Total (95% CI)**		**130**		**57**	**100.0%**	**1.23 [1.03, 1.47]**
Total events	112		40			

Heterogeneity: Chi² = 2.79, df = 2 (P = 0.25); I² = 28%
Test for overall effect: Z = 2.28 (P = 0.02)

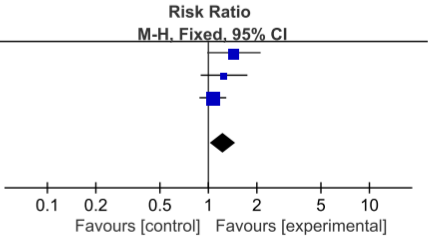

Fig.7e Number of fatigue reduction cases

Study or Subgroup	Experimental Mean	Experimental SD	Experimental Total	Control Mean	Control SD	Control Total	Weight	Mean Difference IV, Random, 95% CI
Fu 2020	0.72	0.21	32	1.06	0.24	33	26.0%	-0.34 [-0.45, -0.23]
Fu XX 2020	2.72	0.25	37	3.86	0.33	36	25.8%	-1.14 [-1.27, -1.01]
Yang MB 2020	0.12	0.33	26	1	0.8	23	21.8%	-0.88 [-1.23, -0.53]
Yu P 2020	0.78	0.25	147	1.12	0.28	148	26.4%	-0.34 [-0.40, -0.28]
**Total (95% CI)**			**242**			**240**	**100.0%**	**-0.66 [-1.05, -0.28]**

Heterogeneity: Tau² = 0.14; Chi² = 123.26, df = 3 (P < 0.00001); I² = 98%
Test for overall effect: Z = 3.40 (P = 0.0007)

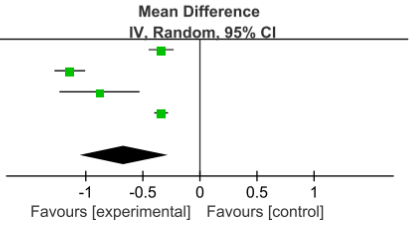

Fig.7f TCM symptom score of fatigue

Fig.7. Clinical symptoms of fever, cough and fatigue. 7a Number of fever reduction cases. 7b TCM symptom score of fever. 7c Number of cough reduction cases. 7d TCM symptom score of cough. 7e Number of fatigue reduction cases. 7f TCM symptom score of fatigue.

Figure

Click here to access/download;Figure;Fig.8. Inflammatory biomarkers..pdf

Study or Subgroup	Experimental Mean	Experimental SD	Experimental Total	Control Mean	Control SD	Control Total	Weight	Mean Difference IV, Fixed, 95% CI
Fu 2020	5.9	0.32	32	5.54	0.34	33	17.1%	0.36 [0.20, 0.52]
Fu XX 2020	5.9	0.36	37	5.64	0.37	36	15.7%	0.26 [0.09, 0.43]
Yu P 2020	5.87	0.36	147	5.46	0.35	148	67.0%	0.41 [0.33, 0.49]
Zhang CT 2020	6.77	2.32	22	7.02	3.1	23	0.2%	-0.25 [-1.85, 1.35]
**Total (95% CI)**			**238**			**240**	**100.0%**	**0.38 [0.31, 0.44]**

Heterogeneity: $Chi^2 = 3.15, df = 3 (P = 0.37); I^2 = 5\%$
Test for overall effect: Z = 11.13 (P < 0.00001)

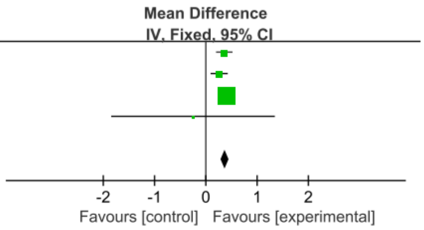

Fig.8a WBC count

Study or Subgroup	Experimental Mean	Experimental SD	Experimental Total	Control Mean	Control SD	Control Total	Weight	Mean Difference IV, Random, 95% CI
Fu 2020	1.84	0.11	32	1.58	0.12	33	33.1%	0.26 [0.20, 0.32]
Fu XX 2020	1.97	0.16	37	1.52	0.11	36	32.9%	0.45 [0.39, 0.51]
Yang MB 2020	2.42	5.39	26	3.39	6.38	23	0.4%	-0.97 [-4.30, 2.36]
Yu P 2020	1.68	0.15	147	1.59	0.18	148	33.6%	0.09 [0.05, 0.13]
**Total (95% CI)**			**242**			**240**	**100.0%**	**0.26 [0.05, 0.47]**

Heterogeneity: $Tau^2 = 0.03; Chi^2 = 98.13, df = 3 (P < 0.00001); I^2 = 97\%$
Test for overall effect: Z = 2.48 (P = 0.01)

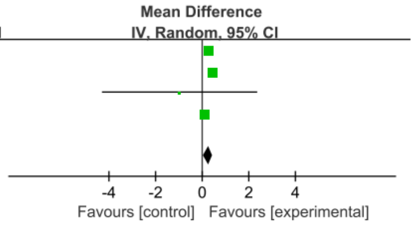

Fig.8b LYM count

Study or Subgroup	Experimental Mean	Experimental SD	Experimental Total	Control Mean	Control SD	Control Total	Weight	Mean Difference IV, Random, 95% CI
Fu 2020	34.03	2	32	28.85	2.4	33	38.4%	5.18 [4.11, 6.25]
Fu XX 2020	32.03	2	37	28.85	2.4	36	38.6%	3.18 [2.17, 4.19]
Zhang CT 2020	37.2	8.63	22	22.3	6.45	23	23.0%	14.90 [10.43, 19.37]
**Total (95% CI)**			**91**			**92**	**100.0%**	**6.65 [3.36, 9.94]**

Heterogeneity: $Tau^2 = 7.05; Chi^2 = 28.81, df = 2 (P < 0.00001); I^2 = 93\%$
Test for overall effect: Z = 3.96 (P < 0.0001)

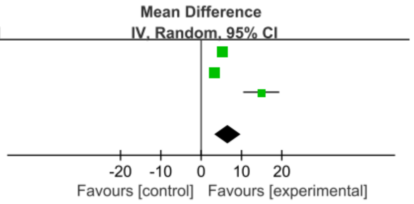

Fig.8c LYM percentage

Study or Subgroup	Experimental Mean	Experimental SD	Experimental Total	Control Mean	Control SD	Control Total	Weight	Mean Difference IV, Fixed, 95% CI
Fu 2020	55.48	2.01	32	60.06	2.98	33	94.5%	-4.58 [-5.81, -3.35]
Yang MB 2020	61.12	6.61	26	65.31	10.86	23	5.5%	-4.19 [-9.30, 0.92]
**Total (95% CI)**			**58**			**56**	**100.0%**	**-4.56 [-5.76, -3.36]**

Heterogeneity: $Chi^2 = 0.02, df = 1 (P = 0.88); I^2 = 0\%$
Test for overall effect: Z = 7.46 (P < 0.00001)

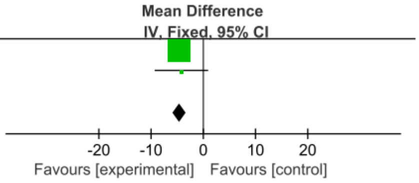

Fig.8d NEU percentage

Study or Subgroup	Experimental Mean	Experimental SD	Experimental Total	Control Mean	Control SD	Control Total	Weight	Mean Difference IV, Random, 95% CI
Fu 2020	22.75	4.8	32	31.86	5.1	33	17.0%	-9.11 [-11.52, -6.70]
Fu XX 2020	24.75	4.8	37	32.86	5.2	36	17.2%	-8.11 [-10.41, -5.81]
Yang MB 2020	2.91	3.02	26	9.73	17.78	23	8.1%	-6.82 [-14.18, 0.54]
Yu P 2020	22.37	4.37	147	24.37	4.37	148	19.1%	-2.00 [-3.00, -1.00]
Zhang CT 2020	8.35	1.03	22	15.23	2.65	23	19.0%	-6.88 [-8.05, -5.71]
Zhou WM 2020	1.9	0.85	52	3.26	1.63	52	19.5%	-1.36 [-1.86, -0.86]
**Total (95% CI)**			**316**			**315**	**100.0%**	**-5.46 [-8.19, -2.72]**

Heterogeneity: $Tau^2 = 9.89; Chi^2 = 128.31, df = 5 (P < 0.00001); I^2 = 96\%$
Test for overall effect: Z = 3.91 (P < 0.0001)

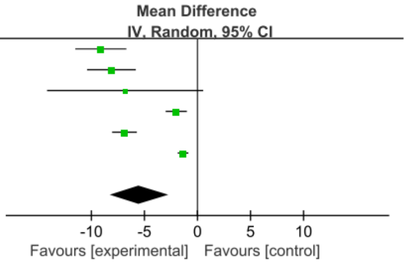

Fig.8e CRP

Fig.8. Inflammatory biomarkers. 8a WBC count. 8b LYM count. 8c LYM percentage. 8d NEU percentage. 8e CRP.

Figure

Click here to access/download;Figure;Fig.9. Adverse effects..pdf

Study or Subgroup	Experimental Events	Experimental Total	Control Events	Control Total	Weight	Risk Ratio M-H, Random, 95% CI
Duan C 2020	27	82	0	41	8.8%	27.83 [1.74, 445.11]
Hu F 2020	1	57	0	71	7.0%	3.72 [0.15, 89.72]
Hu K.2020	65	142	77	142	43.3%	0.84 [0.67, 1.07]
Zhang YL 2020	1	80	0	40	7.0%	1.52 [0.06, 36.46]
Zhou WM 2020	8	52	15	52	34.0%	0.53 [0.25, 1.15]
**Total (95% CI)**		**413**		**346**	**100.0%**	**1.13 [0.45, 2.83]**
Total events	102		92			

Heterogeneity: Tau^2 = 0.49; Chi^2 = 10.95, df = 4 (P = 0.03); I^2 = 63%
Test for overall effect: Z = 0.27 (P = 0.79)

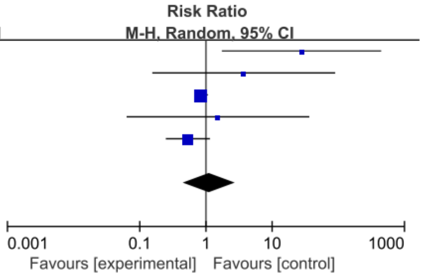

Fig.9a Number of adverse effects cases

Study or Subgroup	Experimental Events	Experimental Total	Control Events	Control Total	Weight	Risk Ratio M-H, Fixed, 95% CI
Hu K.2020	8	142	8	142	72.7%	1.00 [0.39, 2.59]
Zhou WM 2020	4	52	3	52	27.3%	1.33 [0.31, 5.67]
**Total (95% CI)**		**194**		**194**	**100.0%**	**1.09 [0.49, 2.41]**
Total events	12		11			

Heterogeneity: Chi^2 = 0.11, df = 1 (P = 0.74); I^2 = 0%
Test for overall effect: Z = 0.21 (P = 0.83)

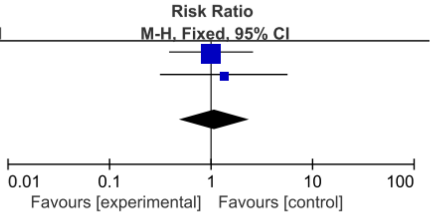

Fig.9b Number of nausea and vomiting cases

Study or Subgroup	Experimental Events	Experimental Total	Control Events	Control Total	Weight	Risk Ratio M-H, Random, 95% CI
Duan C 2020	27	82	0	41	16.6%	27.83 [1.74, 445.11]
Hu F 2020	1	57	0	71	14.3%	3.72 [0.15, 89.72]
Hu K.2020	8	142	19	142	29.9%	0.42 [0.19, 0.93]
Zhang CT 2020	1	80	0	40	14.4%	1.52 [0.06, 36.46]
Zhou WM 2020	3	52	3	52	24.8%	1.00 [0.21, 4.73]
**Total (95% CI)**		**413**		**346**	**100.0%**	**1.72 [0.34, 8.67]**
Total events	40		22			

Heterogeneity: Tau^2 = 2.12; Chi^2 = 13.44, df = 4 (P = 0.009); I^2 = 70%
Test for overall effect: Z = 0.65 (P = 0.51)

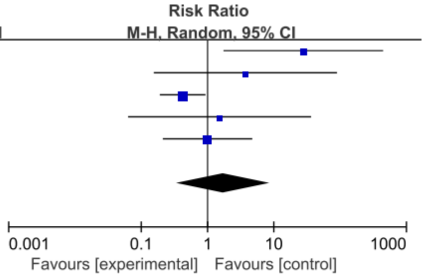

Fig.9c Number of diarrhea cases

Study or Subgroup	Experimental Events	Experimental Total	Control Events	Control Total	Weight	Risk Ratio M-H, Random, 95% CI
Hu K.2020	32	142	32	142	59.9%	1.00 [0.65, 1.54]
Zhou WM 2020	1	52	9	52	40.1%	0.11 [0.01, 0.85]
**Total (95% CI)**		**194**		**194**	**100.0%**	**0.41 [0.05, 3.69]**
Total events	33		41			

Heterogeneity: Tau^2 = 2.03; Chi^2 = 4.63, df = 1 (P = 0.03); I^2 = 78%
Test for overall effect: Z = 0.79 (P = 0.43)

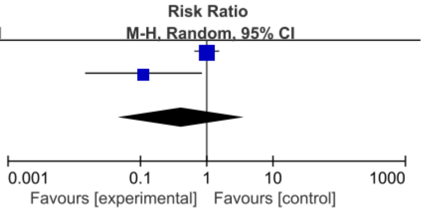

Fig.9d Number of abnormal liver function cases

Fig.9. Adverse effects. 9a Number of adverse effects cases. 9b Number of nausea and vomiting cases. 9c Number of diarrhea cases. 9d Number of abnormal liver function cases.

Table 1
Characteristics of included RCTs.

First author	Type of COVID-19	Sample size (M/F)	Age (years)	Intervention	Control	Duration	Outcome measures
Duan C [10]	mild	T:82(39/43) C:41(23/18)	T:51.99±13.88 C:50.29±13.17	Jinhua Qinggan granule + CWM	CWM including antiviral, anti-infection, and symptomatic therapies	5 days	Clinical symptoms, and adverse effects
Fu [11]	mild/ moderate	T:32(17/15) C:33(19/14)	T:43.26±7.15 C:43.68±6.45	Toujie Quwen granule + CWM	CWM including abidor tablets, moxifloxacin tablets, and ambroxol tablets	10 days	Lung CT, clinical cure rate, rate of conversion to severe cases, clinical symptoms, inflammatory biomarkers, and adverse effects
Fu XX [12]	moderate	T:37(19/18) C:36(19/17)	T:45.26±7.25 C:44.68±7.45	Toujie Quwen granule + CWM	CWM including abidor tablets, and ambroxol tablets	15 days	Clinical cure rate, rate of conversion to severe cases, clinical symptoms, inflammatory biomarkers, and adverse effects
Hu F [13]	moderate	T:100(49/51) C:100(55/45)	T:47.00±14.06 C:49.28±11.14	Jinyinhua oral liquid + CWM	CWM including interferon-α, lopinavir and tonavir tablets, symptomatic and supportive therapies	10 days	Lung CT, virus nucleic acid testing, rate of conversion to severe cases, and adverse effects
Hu K [14]	mild/ moderate	T:142(79/63) C:142(71/71)	T:50.4±15.2 C:51.8±14.8	Lianhua Qingwen capsule + CWM	CWM including oxygen therapy, antiviral, and symptomatic therapies	14 days	Lung CT, clinical cure rate, virus nucleic acid testing, rate of conversion to severe cases, clinical symptoms, and adverse effects
Qiu M [15]	moderate	T:25(13/12) C:25(14/11)	T:53.35±18.35 C:51.32±14.62	Maxing Xuanfei Jiedu Decoction + CWM	CWM including interferon-α, lopinavir and tonavir tablets	10 days	Lung CT, rate of conversion to severe cases, and clinical symptoms

Sun HM [16]	mild/ moderate	T:32(17/15) C:25(11/14)	T:45.4±14.10 C:42.0±11.70	Lianhua Qingke granule + CWM	CWM including interferon-α, lopinavir and tonavir tablets, symptomatic and supportive therapies	14days	Lung CT, rate of conversion to severe cases, and clinical symptoms
Yang MB [17]	moderate	T:26(16/10) C:23(9/14)	T:50.35±13.37 C:47.17±16.57	Reyanning mixture + CWM	CWM including interferon-α, lopinavir and tonavir tablets, abidor tablets, and ribavirin	7 days	Virus nucleic acid testing, rate of conversion to severe cases, clinical symptoms, inflammatory biomarkers, and adverse effects
Yu P [18]	mild/ moderate	T:147(82/65) C:148(89/59)	T:48.27±9.56 C:47.25±8.67	Lianhua Qingwen granule+ CWM	CWM including abidor tablets, moxifloxacin tablets, and ambroxol tablets	7 days	Lung CT, clinical cure rate, rate of conversion to severe cases, clinical symptoms, inflammatory biomarkers, and adverse effects
Zhang CT [19]	moderate	T: 22 (9/ 13) C: 23 (10/13)	T: 53.7 ± 3.5 C: 55.6 ± 4.2	Jiawei Dayuan Decoction + CWM	CWM including oxygen therapy, antivirus, and symptomatic therapies	7 days	Lung CT, clinical symptoms, inflammatory biomarkers, and adverse effects
Zhang YL [20]	moderate	T: 80 (50/ 30) C: 40(23/17)	T: 53.4±13.70 C:52.0±14.10	Jinyinhua oral liquid + CWM	CWM including interferon-α, lopinavir and tonavir tablets, symptomatic and supportive therapies	10 days	Rate of conversion to severe cases, clinical symptoms, and adverse effects
Zhou WM [21]	moderate	T: 52 (32/ 20) C: 52(28/24)	T: 52.47±10.99 C:51.11±9.87	diammonium glycyrrhizinate + CWM	CWM including lopinavir and tonavir tablets, symptomatic and supportive therapies	14 days	Clinical cure rate, inflammatory biomarkers, and adverse effects

Click here to access/download
Supporting Information
S1 Checklist. PRISMA 2009 checklist..doc
