# Supplementary material for: Add-on effect of Chinese herbal medicine in the treatment of mild to moderate COVID-19: A systematic review and meta-analysis
Source: PLoS One. 2021 Aug 20;16(8):e0256429. doi: 10.1371/journal.pone.0256429 (PMC8378756; doi:10.1371/journal.pone.0256429)
Supplement: Attachment — Submitted filename: PONE-D-20-38124_reviewer.pdf [file pone.0256429.s003.pdf]

PLOS ONE

# Chinese herbal medicine in adults with mild to moderate coronavirus disease 2019(COVID-19): A systematic review and meta-analysis

--Manuscript Draft--

| | |
|---|---|
| **Manuscript Number:** | PONE-D-20-38124 |
| **Article Type:** | Research Article |
| **Full Title:** | Chinese herbal medicine in adults with mild to moderate coronavirus disease 2019(COVID-19): A systematic review and meta-analysis |
| **Short Title:** | A systematic review and meta-analysis |
| **Corresponding Author:** | Lipeng Shi<br>Traditional Chinese Medicine Hospital of Dianjiang Chongqing<br>Chongqing, CHINA |
| **Keywords:** | Coronavirus disease 2019, Chinese herbal medicine, Systematic review, Meta-analysis, Randomized controlled trials |
| **Abstract:** | Introduction<br>Coronavirus disease 2019 (COVID-19) infected by SARS-CoV-2 has spread all over the world, which is a serious threat to human life and health. In China's experience in fighting COVID-19, traditional Chinese medicine (TCM), especially Chinese herbal medicine (CHM), has played an important role. Human studies reported the beneficial effects of CHM in the treatment of adult patients with mild to moderate COVID-19. Presently there is no systematic evaluation of the clinical efficacy of CHM in adult patients with mild to moderate COVID-19. Therefore, this review was designed to evaluate the efficacy and safety of CHM in the treatment of adult patients with mild to moderate COVID-19.<br>Methods<br>RCTs about CHM for mild to moderate COVID-19 were searched in the following eight electronic databases: PubMed, EMBASE, Cochrane Central Register of Controlled trials, the Clinical Trials.gov website, China National Knowledge Infrastructure (CNKI), China Science and Technology Journal Database (VIP), Wanfang Database and China Biology Medicine (CBM) from December 2019 to November 2020. Two reviewers independently searched, selected studies and extracted data according to the eligibility criteria. Cochrane Risk of Bias (ROB) tool was used to assess the methodological quality of the included RCTs. And Revman5.3.0 software was used for statistical analysis.<br>Results<br>Twelve eligible RCTs were included with a total sample size of 1393. Our meta-analyses found that compared with the conventional western medicine (CWM) treatment, the effective rate of lung CT [RR=1.26, 95%CI (1.15, 1.38), P ＜0.00001], and clinical cure rate [RR=1.26, 95%CI (1.16, 1.38), P ＜0.00001] of the CHM treatment were better. Besides, CHM could reduce the rate of conversion to severe cases [RR=0.48, 95%CI (0.32, 0.73), P =0.0005], TCM symptom score of fever [MD=-0.62, 95%CI (-0.79, -0.45), P ＜0.00001], the cough cases [RR=1.43, 95%CI (1.16, 1.75), P =0.0006], TCM symptom score of cough[MD=-1.07, 95%CI (-1.29, -0.85), P ＜0.00001], TCM symptom score of fatigue[MD=-0.66, 95%CI (-1.05, -0.28), P =0.0007], CRP[MD=-5.46, 95%CI (-8.19, -2.72), P ＜0.0001], and improve WBC count[MD=0.38, 95%CI (0.31, 0.44), P ＜0.00001], and the above meta-analysis results were robust and reliable through sensitivity analysis.<br>Conclusion<br>Chinese herbal medicine is effective and safe in the treatment of adults with mild to moderate COVID-19. And Chinese herbal medicine may be a promising candidate for the treatment of mild to moderate COVID-19. |
| **Order of Authors:** | Xuqin Du<br>Lipeng Shi<br>Wenfu Cao<br>Biao Zuo |

Powered by Editorial Manager® and ProduXion Manager® from Aries Systems Corporation

| | Aimin Zhou |
|---|---|
| **Additional Information:** | |
| **Question** | **Response** |
| **Financial Disclosure**<br><br>Enter a financial disclosure statement that describes the sources of funding for the work included in this submission. Review the submission guidelines for detailed requirements. View published research articles from *PLOS ONE* for specific examples.<br><br>This statement is required for submission and **will appear in the published article** if the submission is accepted. Please make sure it is accurate.<br><br>**Unfunded studies**<br>Enter: *The author(s) received no specific funding for this work.*<br><br>**Funded studies**<br>Enter a statement with the following details:<br>• Initials of the authors who received each award<br>• Grant numbers awarded to each author<br>• The full name of each funder<br>• URL of each funder website<br>• Did the sponsors or funders play any role in the study design, data collection and analysis, decision to publish, or preparation of the manuscript?<br>• **NO** - Include this sentence at the end of your statement: *The funders had no role in study design, data collection and analysis, decision to publish, or preparation of the manuscript.*<br>• **YES** - Specify the role(s) played.<br><br>* typeset | This study was funded by the National Natural Science Foundation of China (No.81573860) and the postdoctoral research project of Chongqing Medical University (No. R11004). The funders had no role in study design, data collection and analysis, decision to publish, or preparation of the manuscript. |
| **Competing Interests**<br><br>Use the instructions below to enter a competing interest statement for this submission. On behalf of all authors, disclose any competing interests that could be perceived to bias this work—acknowledging all financial support and any other relevant financial or non-financial competing interests. | The authors declare that they have no competing interests. |

Powered by Editorial Manager® and ProduXion Manager® from Aries Systems Corporation

| | |
|---|---|
| This statement **will appear in the published article** if the submission is accepted. Please make sure it is accurate. View published research articles from *PLOS ONE* for specific examples.<br><br>**NO authors have competing interests**<br><br>Enter: *The authors have declared that no competing interests exist.*<br><br>**Authors with competing interests**<br><br>Enter competing interest details beginning with this statement:<br><br>*I have read the journal's policy and the authors of this manuscript have the following competing interests: [insert competing interests here]*<br><br>* typeset | |
| **Ethics Statement**<br><br>Enter an ethics statement for this submission. This statement is required if the study involved:<br><br>• Human participants<br>• Human specimens or tissue<br>• Vertebrate animals or cephalopods<br>• Vertebrate embryos or tissues<br>• Field research<br><br>Write "N/A" if the submission does not require an ethics statement.<br><br>General guidance is provided below. Consult the submission guidelines for detailed instructions. **Make sure that all information entered here is included in the Methods section of the manuscript.** | Ethical approval and patient consent are not required since this is an overview based on published studies. |

Powered by Editorial Manager® and ProduXion Manager® from Aries Systems Corporation

| | |
|---|---|
| **Format for specific study types**<br><br>**Human Subject Research (involving human participants and/or tissue)**<br>• Give the name of the institutional review board or ethics committee that approved the study<br>• Include the approval number and/or a statement indicating approval of this research<br>• Indicate the form of consent obtained (written/oral) or the reason that consent was not obtained (e.g. the data were analyzed anonymously)<br><br>**Animal Research (involving vertebrate animals, embryos or tissues)**<br>• Provide the name of the Institutional Animal Care and Use Committee (IACUC) or other relevant ethics board that reviewed the study protocol, and indicate whether they approved this research or granted a formal waiver of ethical approval<br>• Include an approval number if one was obtained<br>• If the study involved *non-human primates*, add *additional details* about animal welfare and steps taken to ameliorate suffering<br>• If anesthesia, euthanasia, or any kind of animal sacrifice is part of the study, include briefly which substances and/or methods were applied<br><br>**Field Research**<br><br>Include the following details if this study involves the collection of plant, animal, or other materials from a natural setting:<br>• Field permit number<br>• Name of the institution or relevant body that granted permission | |
| **Data Availability**<br><br>Authors are required to make all data underlying the findings described fully available, without restriction, and from the time of publication. PLOS allows rare exceptions to address legal and ethical concerns. See the PLOS Data Policy and FAQ for detailed information. | No - some restrictions will apply |

| | |
|---|---|
| A Data Availability Statement describing where the data can be found is required at submission. Your answers to this question constitute the Data Availability Statement and **will be published in the article**, if accepted.<br><br>**Important:** Stating 'data available on request from the author' is not sufficient. If your data are only available upon request, select 'No' for the first question and explain your exceptional situation in the text box.<br><br>Do the authors confirm that all data underlying the findings described in their manuscript are fully available without restriction? | |
| **Describe where the data may be found in full sentences. If you are copying our sample text, replace any instances of XXX with the appropriate details.**<br><br>• If the data are **held or will be held in a public repository**, include URLs, accession numbers or DOIs. If this information will only be available after acceptance, indicate this by ticking the box below. For example: *All XXX files are available from the XXX database (accession number(s) XXX, XXX.*).<br>• If the data are all contained **within the manuscript and/or Supporting Information files**, enter the following: *All relevant data are within the manuscript and its Supporting Information files.*<br>• If neither of these applies but you are able to provide **details of access elsewhere**, with or without limitations, please do so. For example:<br><br>*Data cannot be shared publicly because of [XXX]. Data are available from the XXX Institutional Data Access / Ethics Committee (contact via XXX) for researchers who meet the criteria for access to confidential data.*<br><br>*The data underlying the results presented in the study are available from (include the name of the third party* | Data cannot be shared publicly because the article data is from a meta-analysis of the published literature. |

| | |
|---|---|
| *and contact information or URL).*<br>• This text is appropriate if the data are owned by a third party and authors do not have permission to share the data.<br><br>* typeset | |
| Additional data availability information: | |

Powered by Editorial Manager® and ProduXion Manager® from Aries Systems Corporation

Manuscript

Click here to access/download;Manuscript;Chinese herbal medicine in adults with COVID-19.pdf

# Chinese herbal medicine in adults with mild to moderate coronavirus disease 2019(COVID-19): A systematic review and meta-analysis

Xuqin Du[1,2], Lipeng Shi [3,*], Wenfu Cao [1,2,4,*] , Biao Zuo[1,2], Aimin Zhou[3]

[1] College of Traditional Chinese Medicine, Chongqing Medical University, Chongqing 400016, People's Republic of China

[2] Chongqing Key Laboratory of Traditional Chinese Medicine for Prevention and Cure of Metabolic Diseases, Chongqing 400016, People's Republic of China

[3] Department of Cardiovascular Unit, Traditional Chinese medicine hospital Dianjiang Chongqing, Chongqing 408300, People's Republic of China

[4] Department of Chinese Traditional Medicine, The First Affiliated Hospital of Chongqing Medical University, Chongqing 400016, People's Republic of China

[*] shilipeng0206@163.com； caowenfu9316@163.com

**Data Availability Statement:** All relevant data are within the manuscript and its Supporting Information files.

**Funding:** This study was funded by the National Natural Science Foundation of China (No.81573860) and the postdoctoral research project of Chongqing Medical University (No. R11004). The funders had no role in study design, data collection and analysis, decision to publish, or preparation of the manuscript.

**Competing interests:** The authors declare that they have no competing interests.

## ABSTRACT

### Introduction

Coronavirus disease 2019 (COVID-19) infected by SARS-CoV-2 has spread all over the world, which is a serious threat to human life and health. In China's experience in fighting COVID-19, traditional Chinese medicine (TCM), especially Chinese herbal medicine (CHM), has played an important role. Human studies reported the beneficial effects of CHM in the treatment of adult patients with mild to moderate COVID-19.

Presently there is no systematic evaluation of the clinical efficacy of CHM in adult patients with mild to moderate COVID-19. Therefore, this review was designed to evaluate the efficacy and safety of CHM in the treatment of adult patients with mild to moderate COVID-19.

**Methods**

RCTs ~~about~~ on CHM for mild to moderate COVID-19 were searched in the following eight electronic databases: PubMed, EMBASE, Cochrane Central Register of Controlled trials, the Clinical Trials.gov website, China National Knowledge Infrastructure (CNKI), China Science and Technology Journal Database (VIP), Wanfang Database and China Biology Medicine (CBM) from December 2019 to November 2020. Two reviewers independently searched, selected studies ^, and extracted data according to the eligibility criteria. Cochrane Risk of Bias (ROB) tool was used to assess the methodological quality of the included RCTs. And Revman (space) 5.3.0 software was used for statistical analysis.

**Results**

Twelve eligible RCTs were included with a total sample size of 1393. Our meta-analyses found that compared with the conventional western medicine (CWM) treatment, the effective rate of lung CT [RR=1.26, 95%CI (1.15, 1.38), $P<0.00001$], and clinical cure rate [RR=1.26, 95%CI (1.16, 1.38), $P<0.00001$] of the CHM treatment were better. Besides, CHM could reduce the rate of conversion to severe cases [RR=0.48, 95%CI (0.32, 0.73), $P=0.0005$], TCM symptom score of fever [MD=-0.62, 95%CI (-0.79, -0.45), $P<0.00001$], the cough cases [RR=1.43, 95%CI (1.16, 1.75), $P=0.0006$], TCM symptom score of cough[MD=-1.07, 95%CI (-1.29, -0.85), $P<0.00001$], TCM symptom score of fatigue[MD=-0.66, 95%CI (-1.05, -0.28), $P=0.0007$], CRP[MD=-5.46, 95%CI (-8.19, -2.72), $P<0.0001$], and improve WBC count[MD=0.38, 95%CI (0.31, 0.44), $P<0.00001$], and the above meta-analysis results were robust and reliable through sensitivity analysis.

The results section is only made up of one long sentence, which make it hard to understand. Also, the spacing is not consistent. This needs to be broken down to several sentence

**Conclusion**

Chinese herbal medicine is effective and safe in the treatment of adults with mild to moderate COVID-19. And Chinese herbal medicine may be a promising candidate for the treatment of mild to moderate COVID-19.

Not advisable to start a sentence with the word 'and'

## Introduction

Kindly rewrite this sentence as it is not gramatically correct

Coronavirus disease 2019 (COVID-19) is a novel coronavirus "2019-nCoV" causing a clinical syndrome dominated by the acute respiratory tract, with a large-scale epidemic [1-2]. Up to now, the epidemic has been basically brought under control in China, but the situation in many countries is still grim. As of November 21, 2020, 16765323 cases have been confirmed worldwide; 57969680 cases have been cumulatively diagnosed; 1375205 cases have died, and the mortality rate is 2.4%. COVID-19 has developed into a global public health emergency. Therefore, it is an urgent task to control COVID-19 effectively.

language check

Ref?

Suggest to use more scientifically accurate words (this apply to the entire manuscript throughout, this is just an example. The word 'basically' here is vague and not a precise word to be used in a scientific manuscript.

In China's experience in fighting COVID-19, traditional Chinese medicine (TCM), especially Chinese herbal medicine (CHM), has played an important role [3]. A large number of epidemiological investigations showed that mild to moderate COVID-19 accounted for the largest proportion [4]. Conventional western medicine (CWM) in the treatment of mild to moderate COVID-19 is mainly antiviral and symptomatic support treatment, so far, no specific drug for the virus has been developed. CHM treatment which is based on syndrome differentiation could effectively alleviate clinical symptoms, reduce the rate of conversion to severe cases, and improve the cure rate [5]. Although ~~there are~~ several reviews ~~of~~ CHM for COVID-19 published [5-7], retrospective studies were included in the review[5-6], and no subgroup analysis of mild to moderate COVID-19 was performed [5-7].

largest proportion of cases

Break into two sentence. .... treatment. So far, no......

evaluating the efficacy of

were

only

break into two sentence. review. Furthermore, no.....

s

'developed' is an inaccurate word here. Sugest to discuss about approved antivirals and their efficacy instead of saying no drug has been developed.

abbreviation not abbreviated on first encounter

[In our review, the RCTs on CHM in the treatment of adult patients with mild to moderate COVID-19 have been searched since the outbreak of the epidemic. And the efficacy and safety of CHM in adults with mild to moderate COVID-19 were objectively evaluated by systematic evaluation and meta-analysis, in order to further provide evidence-based evidence for CHM in the treatment of COVID-19.]

## Methods

Need language check and proof read on sentence structuring, grammar, and words selection.

was conducted

This review was based on the guidelines of Preferred Reporting Items for Systematic Reviews and Meta-Analyses (PRISMA) [8]. Also, the protocol for our review has been registered on the International Prospective Register of Systematic Reviews (PROSPERO) with the registration number CRD42020213528.

Please also check the use of connective words.

**Eligibility criteria**

**Types of studies**

Only RCTs of CHM for adults with mild to moderate COVID-19 were included in this review. The retrospective study, observational study, studies with data duplication, cross-over RCTs, and laboratory studies were excluded.

by laboratory you meant preclinical studies?

Check language, study, studies, singular, plural

**Types of participants**

need to define/ quote reference that define mild to moderate covid-19

Adult patients (aged≥18 years) diagnosed as mild to moderate COVID-19 could be enrolled in this review without the restriction of race or gender.

was included

Identical is a strong word. Are they really identical? This is the method section hence the eligibility criteria should present the inclusion criteria instead e.g. CHM of all formulations. Characteristics of included studies should be presented in results instead

**Types of interventions**

with

Adult patients in the treatment group were treated by a combination of CHM and CWM. The dosage forms of CHM for mild to moderate COVID-19 in this review contained decoction, granule, capsule, and oral liquid. Patients in the control group were treated by CWM. Also, CWM in the treatment group and the control group was identical. Furthermore, RCTs would be excluded if the treatment group was treated with TCM injection, moxibustion, acupuncture, massage, etc.

what is etc. in the exclusion criteria? and what is the rationale of exclusion criteria (for injection) here

**Types of outcome measures**

The primary outcome of this review was lung computed tomography (CT).

suggest to define clearly each outcome, and present them clearly in categories

High-resolution CT was utilized to observe changes in the chest and lung field before and after treatment. The secondary outcomes included the following items: clinical cure rate, viral nucleic acid testing, rate of conversion to severe cases, clinical symptoms (fever, cough, fatigue), inflammatory biomarkers including white blood cell (WBC) count, lymphocyte (LYM) count, LYM percentage, neutrophils (NEU) percentage, C-reactive protein (CRP), and adverse drug reactions (number of adverse effects cases, nausea and vomit, diarrhea, liver damage).

adverse effects? adverse events? related/ not related to drug/CHM? kindly specify

**Literature search**

Here mentioned as adverse events instead

RCTs assessing the efficacy and adverse events of CHM for adults with mild to moderate COVID-19 were searched in the following eight electronic databases: PubMed, EMBASE, Cochrane Central Register of Controlled Trials, the Clinical Trials.gov website, China National Knowledge Infrastructure (CNKI), China Science and Technology Journal Database (VIP), Wanfang Database and China Biology Medicine (CBM) from December 2019 to November 2020. ~~Besides~~, there was no language restriction in our review. ~~And~~, the search terms included “coronavirus disease 2019”, “COVID-19”, “novel coronavirus pneumonia”, “traditional Chinese medicine”, “Chinese herbal medicine”, “Chinese herb”, “clinical trial”, “randomized controlled trial”, “randomised controlled trial”.

use of tenses here needs to be corrected, everything should be in past tense

**Study selection and data extraction**

The study selection and data extraction of our review were conducted independently by two reviewers (Du XQ and Shi LP) according to the eligibility criteria. The following information was ~~would be extracted~~ from the included RCTs: basic characteristics ~~of included RCTs~~ (the title ~~of RCTs~~, first authors’ name, publication date, sample size, and methodological quality), participant characteristics (age, gender, sample size ~~number in each group~~), intervention details (description of ~~the type of~~ interventions, description of ~~type of~~ controls, no duration? dose, route of ~~oral~~ administration), and outcome measures including primary and secondary outcome measures, as well as adverse events. Any disagreements was ~~would be~~ resolved by ~~consultation with~~ a third reviewer (Cao WF).

Methodological quality is part of data analysis, not part of data extracted

**Assessment of methodological quality**

~~According to the Cochrane Collaboration’s tool [9],~~ the methodological quality of the included RCTs was assessed by two reviewers (Du XQ and Shi LP) independently using the Cochrane Collaboration's tool [9]. Seven items of risk of bias (ROB) including adequate sequence generation, concealment of allocation, blinding (patient, investigator and assessor), incomplete outcome data addressed, free of selective reporting, and other biases were evaluated. Each item of ROB was assessed to be low ROB, high ROB, or unclear ROB. Additionally, any disagreements of ROB were resolved by consultation with a third reviewer (Cao WF).

**Statistical analysis** (Suggested title: Meta-analyses)

spacing

Revman5.3.0 software (The Cochrane Collaboration, Copenhagen, Denmark) was
quantitative
used for ~~statistical~~ analysis. The relative risk (RR) was adopted for ~~the~~ dichotomous variables. ~~And the~~ mean difference (MD) or standard mean difference (SMD) were adopted for the continuous variables. ~~Also, we would set the~~ confidence intervals (CIs) were set
with p < 0.005 considered as statistically significant difference. Heterogeneity was assessed with
as 95%. ~~The heterogeneity test utilized~~ the χ2 test and the $I^2$ statistical value. When
was
the $P \geq 0.10$ or $I^2 \leq 50\%$, a fixed-effect model ~~would be~~ adopted ~~to assess the difference~~,
Otherwise    was applied
~~otherwise,~~ a random-effects model ~~would be selected~~. ~~Besides, we would conduct a~~ subgroup analysis of the primary outcome according to different treatment courses.
what are treatment courses?
was
~~And the~~ sensitivity analysis ~~would be~~ performed by removing each included RCT of both primary outcome and secondary outcomes in turn. ~~When the number of included RCTs on an outcome measure was larger than ten, a~~ funnel plot analysis ~~would be~~ was
for outcome measures with more than 10 RCTs
performed to evaluate the reporting bias. ~~Moreover, *P* < 0.05 was considered as a statistical difference.~~

**Results**

**Eligible studies**

is shown
The flow diagram of study selection and identification ~~was showed~~ in Fig. 1. ~~And~~ the characteristics of included RCTs were listed in Table 1. In this review, a total of twelve eligible RCTs were included [10-21]. Among the twelve RCTs [10-21], three were multi-centered trials [13,14,17] and the rest nine were single-centered trials. All twelve RCTs were conducted in mainland China in 2020. One RCT was ~~online~~
online    online
published in English [14], and the rest were ~~online~~ reported in Chinese. The sample size of the included RCTs ranged from 45 to 284 (total 1393). All twelve RCTs assessed the effects of oral CHM combined with CWM compared to CWM alone. The name, usage, dosage of western medicine used in the treatment group were identical to the control group. The treatment duration varied from 5 to 15 days. Seven RCTs [11,14-19] described the effective rate of lung CT. Five RCTs [11-12,14,17,21] described the clinical cure rate. Four RCTs [13-14,17,20] described the viral nucleic acid testing. Nine RCTs [11-18,20] described the rate of conversion to severe cases. Clinical symptoms of fever, cough and fatigue was described in seven RCTs [10-12,16-18,20], of which three RCTs [10,16,20] described number of

fever/cough/fatigue reduction cases, and four RCTs [11-12,18-19] described TCM symptom score of fever/cough/fatigue. Inflammatory biomarkers were described in six RCTs [11-12,17-19,21], of which four RCTs [11-12,18-19] described WBC count, four RCTs [11-12,17-18] described LYM count, three RCTs [11-12,19] described LYM percentage, two RCTs [11,17] described NEU percentage, and six RCTs [11-12,17-19,21] described CRP. Adverse ~~effects~~ events were described in ten RCTs [10-14,17-21].

I am guessing that you meant that there was unclear risk of bias on allocation concealment, blinding and selective reporting. These sentences represent different meaning and needs to be rewritten

**Assessment of methodological quality**

The methodological quality of the included RCTs was assessed according to the Cochrane handbook criteria. As shown in Fig.2a and Fig.2b, green and "+" indicate "Low risk"; yellow and "?" indicate "Unclear". Detailed information on sequence generation of randomization was described in ten trials (~~10/12,~~ 83.33%) [10-18,21]. Detailed information on allocation concealment, blinding of the patient, and blinding of the investigator was not described in this review. One RCT reported blinding of the assessor [14]. All included RCTs described incomplete outcome data addressed. And free of selective reporting and other biases of the included RCTs were unclear.

Attrition bias is scored as 100% low risk which is in contrast to this sentence

**Efficacy and safety assessment**

**Effective rate of lung CT**

Seven RCTs [11,14-19] reported effective rate of lung CT. A significant improvement in lung CT was identified by CHM treatment in this meta-analysis [n=845, RR=1.26, 95%CI (1.15, 1.38), $I^2$=8%, $P<0.00001$] (Fig. 3).

**Clinical cure rate**

Five RCTs evaluated the effects of CHM on clinical cure rate [11-12,14,17,21]. CHM exhibited a significant improvement on clinical cure rate [n=821, RR=1.26, 95%CI (1.16, 1.38), $I^2$=0%, $P<0.00001$] (Fig. 4).

**Viral nucleic acid testing**

Viral nucleic acid testing was reported in four RCTs [13-14,17,20]. Compared with CWM, no statistical difference on viral nucleic acid testing was identified [n=581, RR=1.09, 95%CI (0.98, 1.21), $I^2$=57%, $P$=0.13] (Fig. 5).

**Rate of conversion to severe cases**

Rate of conversion to severe cases was reported in nine RCTs [11-18,20]. CHM significantly reduced the rate of conversion to severe cases [n=1121, RR=0.48, 95%CI (0.32, 0.73), $I^2 = 0\%$, $P$=0.0005] (Fig. 6).

**Clinical symptoms of fever, cough and fatigue**

Clinical symptoms of fever, cough and fatigue was reported in seven RCTs [10-12,16-18,20]. Among them, three RCTs [10,16,20] reported number of fever/cough/fatigue reduction cases, and four RCTs [11-12,17-18] reported TCM symptom score of fever/cough/fatigue.

Please consider rewriting this whole section with clearer sentences

Meta-analysis revealed no statistical difference on the number of fever reduction treated groups cases between CHM and CWM [n=205, RR=1.14, 95%CI (0.58, 2.25), $I^2$=95%, $P$=0.70] (Fig.7a). TCM symptom score of fever is was significantly reduced by CHM [n=482, MD=-0.62, 95%CI (-0.79, -0.45), $I^2$=79%, $P<0.00001$] (Fig.7b).

CHM significantly reduced the cough cases [n=205, RR=1.43, 95%CI (1.16, 1.75), $I^2 = 0\%$, $P$=0.0006] (Fig.7c); as well as a significant reduction in TCM symptom score of cough was identified by CHM [n=482, MD=-1.07, 95%CI (-1.29, -0.85), $I^2$=84%, $P<0.00001$] (Fig.7d).

It has been identified that fatigue cases is reduced by CHM[n=205, RR=1.23, 95%CI (1.03, 1.47), $I^2$=28%, $P$=0.02] (Fig.7e); also a significant reduction in TCM symptom score of fatigue by CHM [n=482, MD=-0.66, 95%CI (-1.05, -0.28), $I^2$=98%, $P$=0.0007] (Fig.7f).

**Inflammatory biomarkers**

Inflammatory biomarkers were reported in six RCTs [11-12,17-19,21], of which four RCTs [11-12,18-19] reported WBC count, four RCTs [11-12,17-18] reported LYM count, three RCTs [11-12,19] reported LYM percentage, two RCTs [11,17] reported NEU percentage, and six RCTs [11-12,17-19,21] reported CRP.

Meta-analysis revealed a significant improvement on WBC count by CHM [n=478, MD=0.38, 95%CI (0.31, 0.44), $I^2$=5%, $P < 0.00001$] (Fig.8a); a significant improvement on LYM count by CHM [n=482, MD=0.26, 95%CI (0.05, 0.47), $I^2$=97%, $P$=0.01] (Fig.8b); a significant improvement on LYM percentage by CHM

[n=183, MD=6.65, 95%CI (3.36, 9.94), $I^2$=93%, $P<0.0001$] (Fig.8c); a significant reduction in NEU percentage by CHM [n=114, MD=-4.56, 95%CI (-5.76, -3.36), $I^2$=0%, $P<0.00001$] (Fig.8d); a significant reduction in CRP by CHM [n=631, MD=-5.46, 95%CI (-8.19, -2.72), $I^2$=96%, $P<0.0001$] (Fig.8e).

**Adverse effects** adverse events? effects? Suggest to select a consistent term according to correct definition

In this review, adverse effects were reported in ten RCTs [10-14,17-21], ~~and the rest~~ while the remaining two RCTs [15-16] did not ~~describe drug adverse effects~~. Among them, no adverse effect was identified in both treatment and control groups [11-12,17-19]. Adverse effects in the rest five RCTs included gastrointestinal reactions (diarrhea, poor appetite, nausea, vomiting), headache, and abnormal liver function [10,13-14,20-21]. All reported adverse reactions were mild in the treatment and control groups, and were tolerable or alleviated after withdrawal. how many studies?

Compared with CWM, there was no statistical difference in the total number of adverse effects cases [n=759, RR=1.13, 95%CI (0.45, 2.83), $I^2$=63%, $P$=0.79] (Fig.9a); Sub group analysis revealed no statistical difference in the number of nausea and vomiting ~~cases~~ [n=388, RR=1.09, 95%CI (0.49, 2.41), $I^2$=0%, $P$=0.83] (Fig.9b); ~~no statistical difference in the number of~~ diarrhea ~~cases~~ [n=759, RR=1.72, 95%CI (0.34, 8.67), $I^2$=70%, $P$=0.51] (Fig.9c); ~~no statistical difference in the number of~~ and abnormal liver function cases [n=388, RR=0.41, 95%CI (0.05, 3.69), $I^2$=78%, $P$=0.43] (Fig.9d). ~~Additionally, the~~ poor appetite and headache were reported in one RCT [14]~~, and~~ with no statistical difference was identified between CHM and CWM.groups.

**Subgroup analysis of the primary outcomes**

Subgroup analysis of the primary outcomes ~~was~~ is shown in Fig.2. Subgroup analysis revealed an improvement on lung CT of 7days treatment duration by CHM [n=845, RR=1.18, 95%CI (1.02, 1.36), $I^2$ =44%, $P$=0.03] (Fig.3); a significant improvement on lung CT of 10 to 14 days treatment duration by CHM [n=845, RR=1.34, 95%CI (1.19, 1.50), $I^2$ = 0%, $P<0.00001$] (Fig. 3).

**Sensitivity analysis**

Sensitivity analysis revealed that there was a small change in the effect amount, and was a significant difference in effective rate of lung CT, clinical cure rate, rate of

conversion to severe cases, TCM symptom score of fever, number of cough reduction cases, TCM symptom score of cough, TCM symptom score of fatigue, WBC count, and CRP, which indicated the above meta-analysis results to be robust and reliable.

**Publication bias**

As the number of RCTs in any comparative outcome measure was less than ten, we did not assess the publication bias.

**Discussion**

[Since December 2019, COVID-19 infected by SARS-CoV-2 has spread all over the world, which is a serious threat to human life and health. COVID-19 is highly contagious and has a long incubation period. It is generally susceptible to human infection and can be transmitted to each other [22]. In addition, severe cases are more likely to have serious complications, such as shock, ARDS (abbreviation?), arrhythmia and acute heart injury[23-24], which significantly increases the difficulty and cost of treatment. Therefore, it is of great significance to prevent COVID-19 from developing from mild and moderate to severe. Since the outbreak of COVID-19, CHM has been widely used to control COVID-19 in China. To our knowledge, this review would be the first one, which objectively assesses the efficacy and safety of CHM in the treatment of adults with mild to moderate COVID-19.]

this section should be in introduction instead, however, repeat information should be avoided

~~This review systematically evaluated the efficacy and safety of CHM in the treatment of adult patients with mild to moderate COVID-19. After a comprehensive search of eight databases, twelve RCTs were enrolled in our review.~~ The meta-analysis results (meta-analyses (plural)) showed that compared with CWM, ~~CHM combined with CWM has a better therapeutic effect~~.

mild to moderate COVID-19 patients treated with both CHM and CWM had better outcomes in the parameters assessed in this review including xx, xx, and xx.

According to the theory of traditional Chinese medicine, COVID-19 belongs to epidemic disease.

I suggest more explanation of how theory of traditional chinese medicine translates this to belong to epidemic disease.

The pathogenesis of mild to moderate COVID-19 is dampness-heat or cold-dampness obstructing the lung. Therefore, the treatment principles of heat-clearing, eliminating dampness, resolving phlegm and dispersing cold are widely used. In the included studies, nine different oral CHM were used, including Jinhua Qinggan granule [10], Toujie Quwen granule [11-12], Jinyinhua oral liquid [13,20], Lianhua Qingwen capsule (granule) [14,18], Maxing Xuanfei Jiedu Decoction [15], Lianhua Qingke granule [16], Reyanning mixture [17],

Jiawei Dayuan Decoction [19], diammonium glycyrrhizinate [21]. Among the nine oral CHM, the most frequently used Chinese medicine was honeysuckle, which was used in seven trials (7~~/12~~, 58.33%) [10-14,18,20], followed by forsythia (6~~/12~~, 50.00%) [10-12,14,16,18], and ephedra (6~~/12~~, 50.00%) [10,14-16,18-19]. Honeysuckle and forsythia have the function of clearing heat-toxicity and dispersing wind-heat in the theory of TCM. ref? Honeysuckle polysaccharide is an active component of honeysuckle, which can regulate non-specific immunity [25], inhibit the expression of inflammatory factors TNF-α and IL-1β [26], and inhibit a variety of viruses [27]. Phillyrin is an active component of forsythia, which has antiviral and anti-inflammatory activities [28-29]. Ephedra has the function of dissipating cold and diffusing the lung to calm panting in TCM theory. ref? Ephedrine is an active component of ephedra, which can increase the production of anti-inflammatory cytokines IL-10, reduce the production of pro-inflammatory cytokines TNF-α and IL-12[30], and play an antiviral role by inhibiting viral replication [31].

In this review, it was found that CHM was effective and safe for adult patients with mild to moderate COVID-19. CHM could not only improve the lung CT, clinical cure rate and the main clinical symptoms (fever, cough and fatigue), but also reduce the rate of conversion to severe cases, and regulate the inflammatory response, with fewer adverse reactions. Therefore, CHM may be a promising candidate for the treatment of adults with mild to moderate COVID-19.

**Limitations**

The limitations of this review were as follows. First of all, most of the included RCTs had deficiencies in methodology design, including hidden allocation and inadequate reporting of blind methods. Secondly, the composition, dosage and frequency of CHM were different in the treatment group. Thirdly, the multicenter RCTs were lacking. In addition, the duration of the included studies ranged from 5 to 15 days. Therefore, it may be necessary to design more high-quality RCTs with a multicenter, large sample and longer follow-up to better observe the efficacy and possible adverse reactions of CHM in the treatment of adults with mild to moderate COVID-19.

**Conclusion**

CHM is effective and safe in the treatment of adults with mild to moderate COVID-19. It can improve the clinical cure rate, main clinical symptoms, imaging and laboratory indexes, and reduce the rate of conversion to severe cases. However, limited to the fact that COVID-19 is a sudden disease, it is difficult to carry out double-blind clinical trials, which results in the insufficient methodology of the existing-related trials. Therefore, more high-quality trials are needed to evaluate the efficacy and safety of CHM in the treatment of adults with mild to moderate COVID-19 in the future.

## Supporting information

**S1 Checklist. PRISMA 2009 checklist.**

(DOC)

## Author Contributions

**Conceptualization:** Lipeng Shi, Wenfu Cao.

**Data curation:** Xuqin Du, Lipeng Shi.

**Formal analysis:** Xuqin Du.

**Funding acquisition:** Xuqin Du.

**Investigation:** Xuqin Du, Lipeng Shi.

**Methodology:** Xuqin Du, Lipeng Shi.

**Software:** Xuqin Du.

**Supervision:** Wenfu Cao.

**Validation:** Biao Zuo, Aimin Zhou.

**Writing – original draft:** Xuqin Du.

**Writing – review & editing:** Xuqin Du, Lipeng Shi, Wenfu Cao, Biao Zuo, Aimin Zhou.

## References

1. Huang C, Wang Y, Li X, et al. Clinical features of patients infected with 2019 novel coronavirus in Wuhan, China[J]. Lancet. 2020, 395(10223): 497-506.

2. Ralph R, Lew J, Zeng T, et al. 2019-nCoV (Wuhan virus), a novel Coronavirus: human-to-human transmission, travel-related cases, and vaccine readiness [J]. J Infect Dev Ctries, 2020, 14(1): 3-17.
3. Ren JL, Zhang AH, Wang XJ. Traditional Chinese medicine for COVID-19 treatment[J]. Pharmacol Res, 2020, 155:104743.
4. Wu ZY, McGoogan JM. Characteristics of and important lessons from the coronavirus disease 2019(COVID-19) outbreak in China[J]. J Am Med Assoc, 2020, 323(13): 1239-1242.
5. Xiong X, Wang P, Su K, et al. Chinese herbal medicine for coronavirus disease 2019: A systematic review and meta-analysis[J]. Pharmacol Res, 2020, 160:105056.
6. Zeng M, Li L, Wu Z. Traditional Chinese medicine Lianhua Qingwen treating corona virus disease 2019(COVID-19): Meta-analysis of randomized controlled trials[J]. PLoS One, 2020, 15(9):e0238828.
7. Liu M, Gao Y, Yuan Y, et al. Efficacy and safety of integrated traditional Chinese and western medicine for corona virus disease 2019 (COVID-19): a systematic review and meta-analysis[J]. Pharmacol Res, 2020, 158:104896.
8. Moher D, Liberati A, Tetzlaff J, et al. Preferred reporting items for systematic reviews and meta-analyses: the PRISMA statement[J]. Ann Intern Med, 2009,151(4):264-W64.
9. Higgins JPT, Green S. Cochrane Reviewers' Handbook 5.3.0 [updated March 2014], Review Manager (RevMan) [Computer program]. Version 5.3.0. Available from www.cochrane-handbook.org
10. Duan C, Xia GW, Zheng CJ, et al. Clinical observation on Jinhua Qinggan granule combined with conventional western medicine therapy in treating mild cases of coronavirus disease 2019 [J]. J Tradit Chin Med,2020,61(17):1473-1477.
11. Fu XX, Lin LP, Tan XX. Clinical observation on effect of Toujie Quwen granules in treatment of COVID-19 [J]. Chin J Exp Tradit Med Formul,2020,26(12):44-48.
12. Fu XX, Lin LP, Tan XX. Clinical study on 37 case of COVID- 19 treated with integrated traditional Chinese and western medicine [J]. Tradit Chin Drug Res

Clin Pharmacol,2020,31(05):600-604.

13. Hu F, Guo AH, Huang L, et al. Multi-center clinical observation of Jinyinhua oral liquid combined with western medicine in treatment of moderate COVID-19 [J/OL]. J Tradit Chin Med:1-6[2020-11-4]. http://kns.cnki.net/kcms/detail/11.2166.R.20200819.0837.002.html.
14. Hu K, Guan WJ, Bi Y, et al. Efficacy and safety of Lianhuaqingwen capsules, a repurposed Chinese herb, in patients with coronavirus disease 2019: A multicenter, prospective, randomized controlled trial [published online ahead of print, 2020 May 16]. Phytomedicine, 2020,153242. https://doi.org/10.1016/j.phymed.2020.153242.
15. Qiu M, Li Q T, Zhu DP, et al. Efficacy observation of Maxing Xuanfei Jiedu decoction on moderate COVID-19 patients[J]. J Emerg Tradit Chin Med,2020,29(07):1129-1130+1132.
16. Sun HM, Xu F, Zhang L, et al. Study on clinical efficacy of Lianhua Qingke granule in treatment of mild and ordinary COVID-19 [J]. Chin J Exp Tradit Med Formul,2020,26(14):29-34.
17. Yang MB, Dang SS, Haung S, et al. Multi-center clinical observation of Reyanning mixture in treatment of COVID-19 [J]. Chin J Exp Tradit Med Formul,2020,26(14):7-12.
18. Yu P, Li YZ, Wan SB, et al. Clinical efficacy of Lianhua Qingwen granule combined with abidol in treating coronavirus disease 2019 [J]. Chin Pharm J,2020,55(12):1042-1045.
19. Zhang CT, Yang Y, You FM, et al. Clinical study on COVID-19 from the perspective of "Yidujiashi" theory [J]. Pharmacol Clin Chin Materia Medica,2020,36(02):43-45.
20. Zhang YL, Lei L, Xu Y, et al. Clinical efficacy of Jinyinhua oral liquid in the treatment of 80 patients with coronavirus disease 2019 [J]. Chin Pharm,2020,29(09):23-26.
21. Zhou WM, Zhao FM, Li BL, et al. Clinical value of diammonium glycyrrhizinate in treatment of COVID-19 [J]. Chin J Virol,2020,36(02):160-164.

22. Li Q, Guan X, Wu P, et al. Early Transmission Dynamics in Wuhan, China, of Novel Coronavirus-Infected Pneumonia [J]. N Engl J Med, 2020,382(13):1199-1207.
23. Chen N, Zhou M, Dong X, et al. Epidemiological and clinical characteristics of 99 cases of 2019 novel coronavirus pneumonia in Wuhan, China: a descriptive study [J]. Lancet, 2020,395(10223):507-513.
24. Wang DW, Hu B, Hu C, et al. Clinical characteristics of 138 hospitalized patients with 2019 novel coronavirus-infected pneumonia in Wuhan, China [J]. JAMA, 2020, 323(11): 1061-1069.
25. Zhou XN, Dong Q, Kan XZ, et al. Immunomodulatory activity of a novel polysaccharide from Lonicera japonica immunosuppressed mice induced by cyclophosphamide [J]. PloS one,2018,13(10):1-15.
26. Bai XY, Chai YW, Shi WL, et al. Lonicera japonica poly-saccharides attenuate ovalbumin-induced allergic rhinitis by regulation of Th17 cells in BALB/c mice[J]. J Functional Foods,2020,65:103758.
27. Wang J, Hou L, Chen YQ, et al. Extraction and purification of the Lonicera japonica polysaccharide and its antiviral activity in vitro[J]. Chin J Hosp Pharm, 2018,38(8): 810-812.
28. Qu XY, Li QJ, Zhang HM, et al. Protective effects of phillyrin against influenza a virus in vivo[J]. Arch Pharm Res, 2016, 39(7):998-1005.
29. Jiang Q, Chen J, Long X, et al. Phillyrin protects mice from traumatic brain injury by inhibiting the inflammation of microglia via PPARγ signaling pathway[J]. Int Immunopharmacol, 2020,79:106083.
30. He W, Ma J, Chen Y, et al. Ephedrine hydrochloride protects mice from staphylococcus aureus-induced peritonitis[J]. Am J Transl Res, 2018,10(3):670-683.
31. Wei W, Du H, Shao C, et al. Screening of antiviral components of Ma Huang Tang and investigation on the ephedra alkaloids efficacy on influenza virus type A[J]. Front Pharmacol, 2019,10:961.

**The figure legend:**

Fig. 1. Flow diagram of study selection and identification.

Fig.2.Assessment of methodological quality.

Fig.3 Effective rate of lung CT.

Fig.4 Clinical cure rate.

Fig.5 Viral nucleic acid testing.

Fig.6 Rate of conversion to severe cases.

Fig.7. Clinical symptoms of fever, cough and fatigue.

Fig.8. Inflammatory biomarkers.

Fig.9. Adverse effects.

**The table:**

Table 1 Characteristics of included RCTs.

Figure

Click here to access/download;Figure;Fig. 1. Flow diagram of study selection and identification..pdf

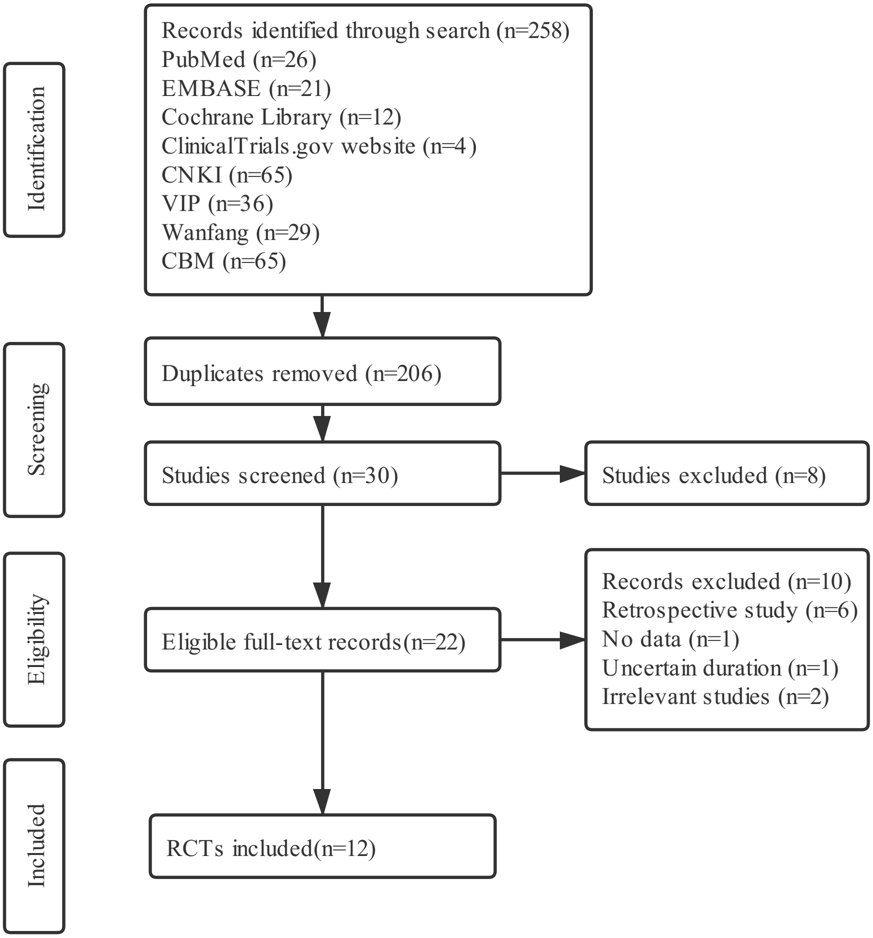


Fig. 1. Flow diagram of study selection and identification.

Figure

Click here to access/download;Figure;Fig.2.Assessment of methodological quality..pdf

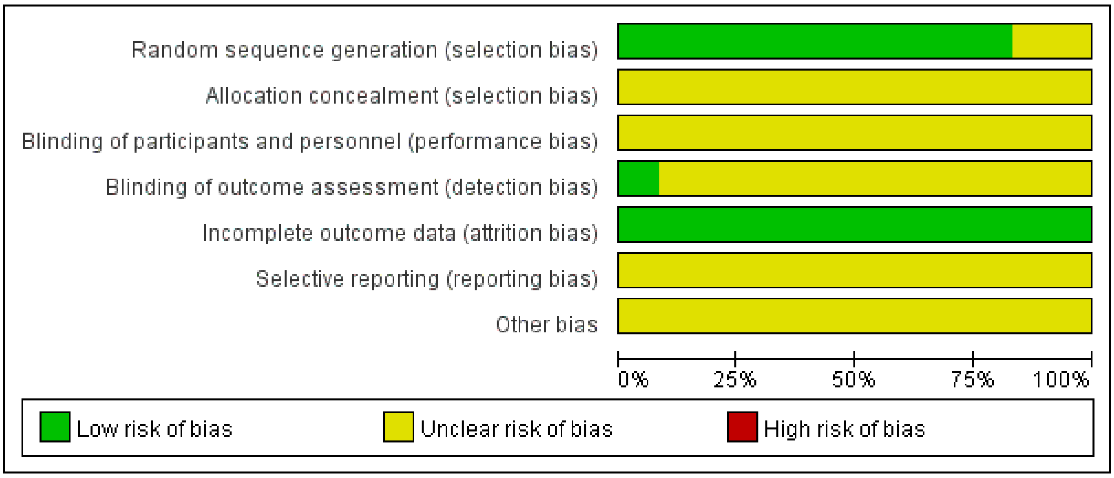


Fig.2a Risk of bias graph.

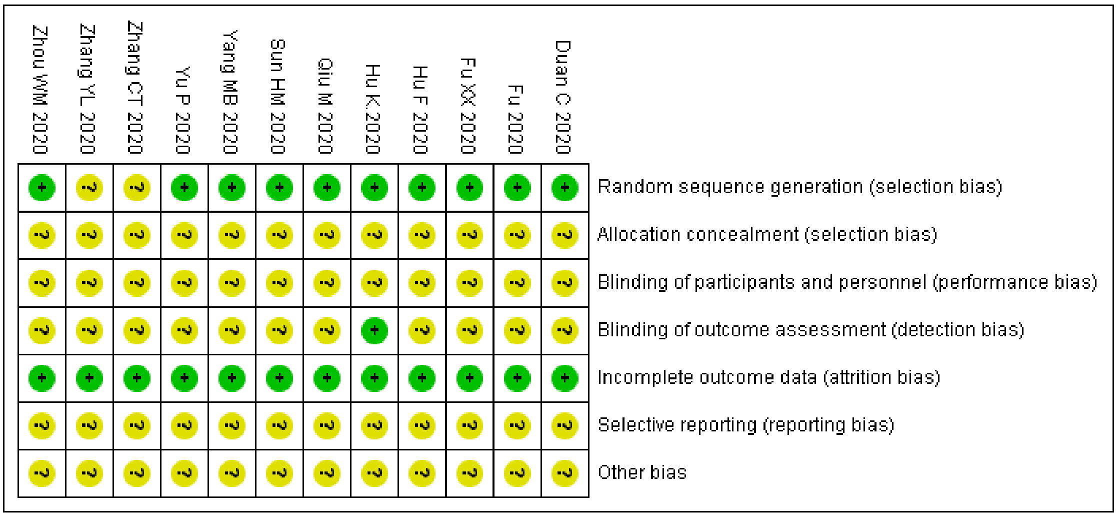


Fig.2b Risk of bias summary.

Fig.2. Assessment of methodological quality. 2a Risk of bias graph. 2b Risk of bias summary.

Figure

Click here to access/download;Figure;Fig.3. Effective rate of lung CT.pdf

| Study or Subgroup | Experimental Events | Experimental Total | Control Events | Control Total | Weight | Risk Ratio M-H, Fixed, 95% CI |
|---|---|---|---|---|---|---|
| **1.1.1 Treatment duration of 7 days** | | | | | | |
| Yang MB 2020 | 23 | 26 | 17 | 23 | 7.3% | 1.20 [0.90, 1.58] |
| Yu P 2020 | 102 | 147 | 93 | 148 | 37.3% | 1.10 [0.94, 1.30] |
| Zhang CT 2020 | 12 | 22 | 5 | 23 | 2.0% | 2.51 [1.06, 5.95] |
| **Subtotal (95% CI)** | | **195** | | **194** | **46.5%** | **1.18 [1.02, 1.36]** |
| Total events | 137 | | 115 | | | |
| Heterogeneity: Chi² = 3.55, df = 2 (P = 0.17); I² = 44% | | | | | | |
| Test for overall effect: Z = 2.22 (P = 0.03) | | | | | | |
| **1.1.2 Treatment duration of 10 to 14 days** | | | | | | |
| Fu 2020 | 26 | 32 | 20 | 33 | 7.9% | 1.34 [0.97, 1.85] |
| Hu K.2020 | 119 | 142 | 91 | 142 | 36.6% | 1.31 [1.13, 1.51] |
| Qiu M 2020 | 5 | 25 | 2 | 25 | 0.8% | 2.50 [0.53, 11.70] |
| Sun HM 2020 | 31 | 32 | 18 | 25 | 8.1% | 1.35 [1.05, 1.73] |
| **Subtotal (95% CI)** | | **231** | | **225** | **53.5%** | **1.34 [1.19, 1.50]** |
| Total events | 181 | | 131 | | | |
| Heterogeneity: Chi² = 0.72, df = 3 (P = 0.87); I² = 0% | | | | | | |
| Test for overall effect: Z = 4.78 (P < 0.00001) | | | | | | |
| **Total (95% CI)** | | **426** | | **419** | **100.0%** | **1.26 [1.15, 1.38]** |
| Total events | 318 | | 246 | | | |
| Heterogeneity: Chi² = 6.50, df = 6 (P = 0.37); I² = 8% | | | | | | |
| Test for overall effect: Z = 4.96 (P < 0.00001) | | | | | | |
| Test for subgroup differences: Chi² = 1.74, df = 1 (P = 0.19), I² = 42.4% | | | | | | |

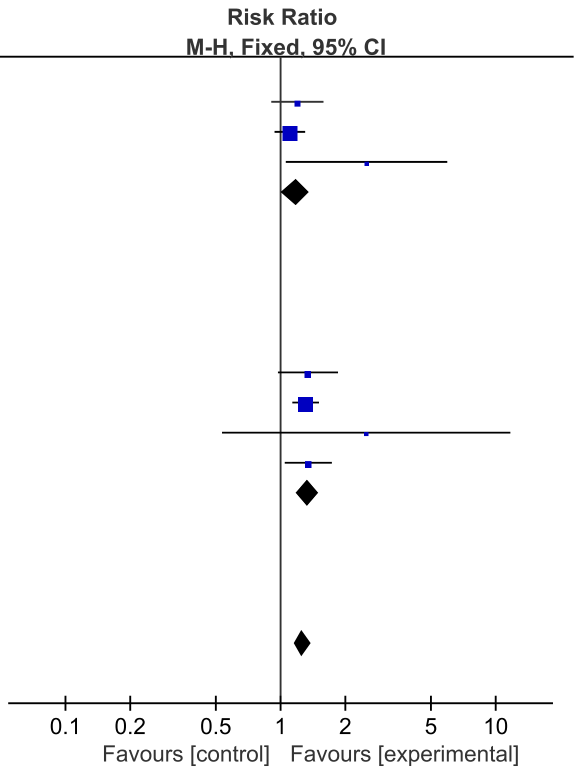


Fig.3 Effective rate of lung CT

Figure
Click here to access/download;Figure;Fig.4. Clinical cure rate.pdf

| Study or Subgroup | Experimental Events | Experimental Total | Control Events | Control Total | Weight | Risk Ratio M-H, Fixed, 95% CI |
|---|---|---|---|---|---|---|
| Fu 2020 | 30 | 32 | 23 | 33 | 8.8% | 1.35 [1.06, 1.71] |
| Fu XX 2020 | 33 | 37 | 25 | 36 | 9.8% | 1.28 [1.01, 1.64] |
| Hu K.2020 | 119 | 147 | 96 | 148 | 37.0% | 1.25 [1.08, 1.44] |
| Yu P 2020 | 112 | 142 | 94 | 142 | 36.3% | 1.19 [1.03, 1.38] |
| Zhou WM 2020 | 32 | 52 | 21 | 52 | 8.1% | 1.52 [1.03, 2.26] |
| **Total (95% CI)** | | **410** | | **411** | **100.0%** | **1.26 [1.16, 1.38]** |
| Total events | 326 | | 259 | | | |

Heterogeneity: Chi² = 1.79, df = 4 (P = 0.77); I² = 0%
Test for overall effect: Z = 5.21 (P < 0.00001)

Risk Ratio
M-H, Fixed, 95% CI

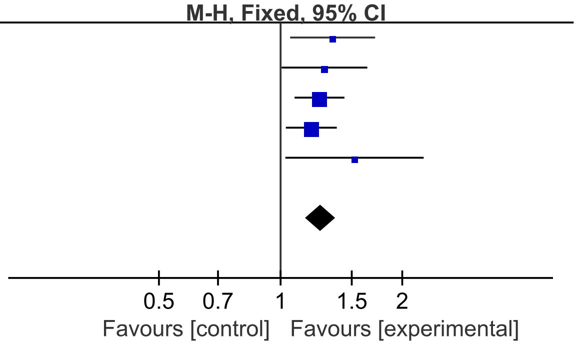


Fig.4 Clinical cure rate

Figure

Click here to access/download;Figure;Fig.5. Viral nucleic acid testing.pdf

| | Experimental | | Control | | | Risk Ratio |
|---|---|---|---|---|---|---|
| **Study or Subgroup** | **Events** | **Total** | **Events** | **Total** | **Weight** | **M-H, Random, 95% CI** |
| Hu F 2020 | 53 | 57 | 62 | 71 | 31.4% | 1.06 [0.95, 1.19] |
| Hu K.2020 | 109 | 142 | 101 | 142 | 27.0% | 1.08 [0.94, 1.24] |
| Yang MB 2020 | 25 | 26 | 14 | 23 | 8.6% | 1.58 [1.13, 2.21] |
| Zhang YL 2020 | 75 | 80 | 37 | 40 | 33.1% | 1.01 [0.91, 1.13] |
| **Total (95% CI)** | | **305** | | **276** | **100.0%** | **1.09 [0.98, 1.21]** |
| Total events | 262 | | 214 | | | |

Heterogeneity: Tau² = 0.01; Chi² = 6.90, df = 3 (P = 0.08); I² = 57%

Test for overall effect: Z = 1.51 (P = 0.13)

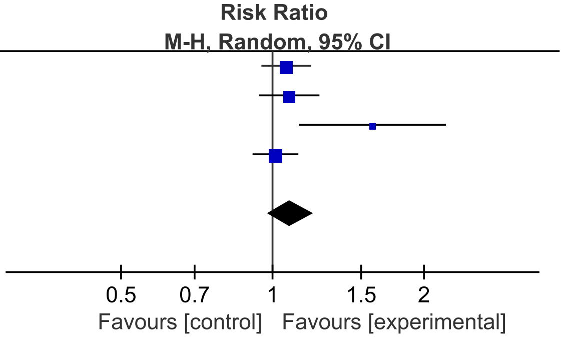


Fig. 5 Viral nucleic acid testing

Figure

Click here to access/download;Figure;Fig.6. Rate of conversion to severe cases.pdf

| | Experimental | | Control | | | Risk Ratio |
|---|---|---|---|---|---|---|
| Study or Subgroup | Events | Total | Events | Total | Weight | M-H, Fixed, 95% CI |
| Fu 2020 | 1 | 32 | 3 | 33 | 4.9% | 0.34 [0.04, 3.13] |
| Fu XX 2020 | 2 | 37 | 4 | 36 | 6.8% | 0.49 [0.09, 2.49] |
| Hu F 2020 | 0 | 57 | 3 | 71 | 5.2% | 0.18 [0.01, 3.36] |
| Hu K.2020 | 3 | 142 | 6 | 142 | 10.0% | 0.50 [0.13, 1.96] |
| Qiu M 2020 | 0 | 25 | 1 | 25 | 2.5% | 0.33 [0.01, 7.81] |
| Sun HM 2020 | 0 | 32 | 2 | 25 | 4.7% | 0.16 [0.01, 3.14] |
| Yang MB 2020 | 0 | 26 | 0 | 23 | | Not estimable |
| Yu P 2020 | 21 | 147 | 35 | 148 | 58.2% | 0.60 [0.37, 0.99] |
| Zhang YL 2020 | 0 | 80 | 3 | 40 | 7.8% | 0.07 [0.00, 1.37] |
| **Total (95% CI)** | | **578** | | **543** | **100.0%** | **0.48 [0.32, 0.73]** |
| Total events | 27 | | 57 | | | |

Heterogeneity: Chi² = 3.54, df = 7 (P = 0.83); I² = 0%

Test for overall effect: Z = 3.48 (P = 0.0005)

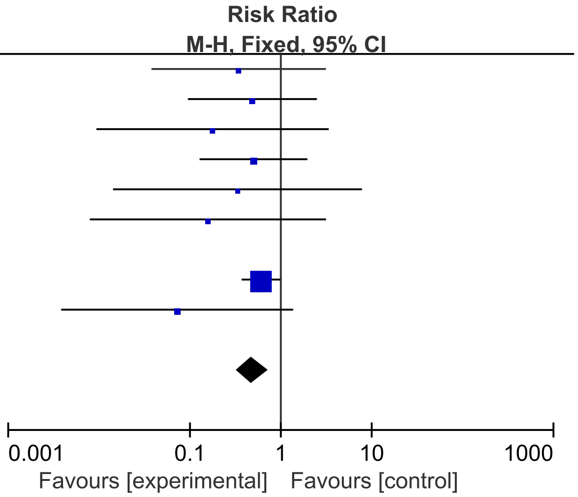


Fig. 6 Rate of conversion to severe cases

Figure
Click here to access/download;Figure;Fig.7. Clinical symptoms of fever, cough and fatigue..pdf

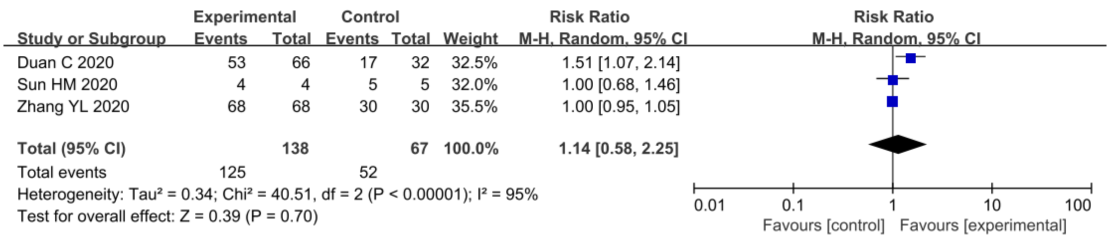


Fig.7a Number of fever reduction cases

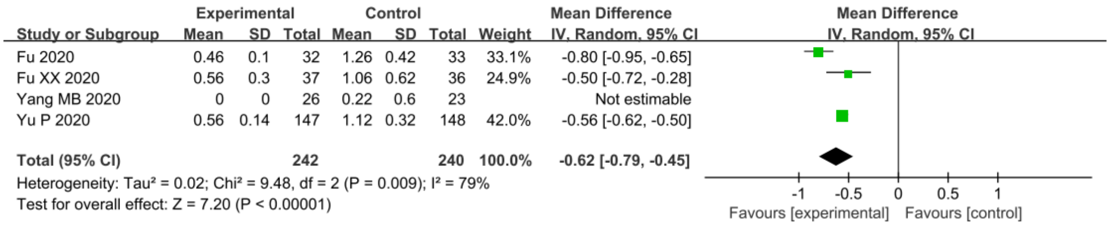


Fig.7b TCM symptom score of fever

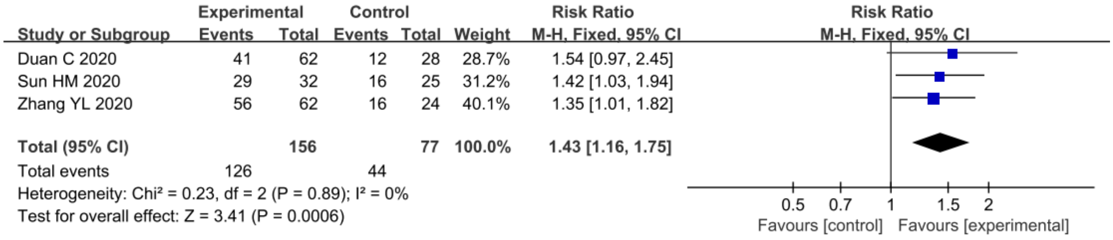


Fig.7c Number of cough reduction cases

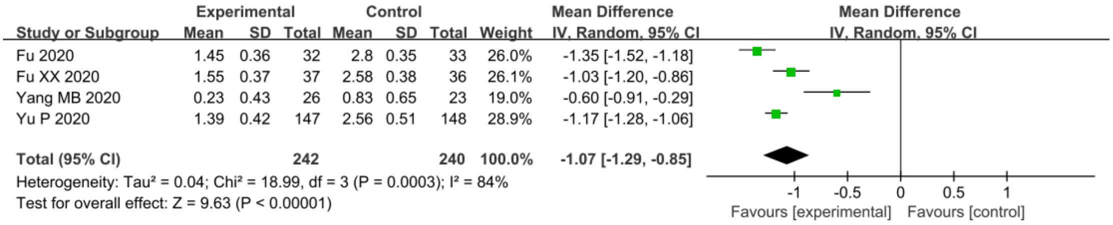


Fig.7d TCM symptom score of cough

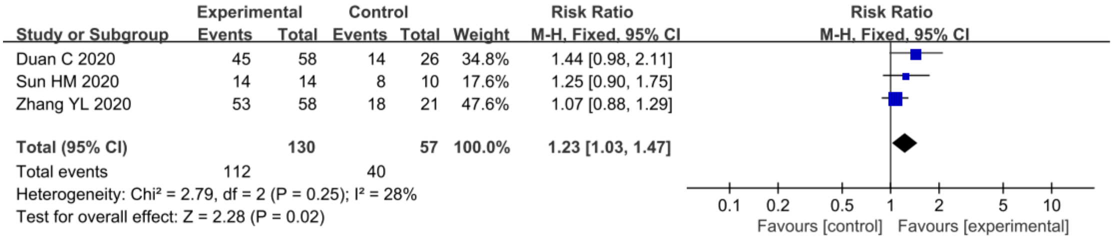


Fig.7e Number of fatigue reduction cases

| Study or Subgroup | Experimental Mean | Experimental SD | Experimental Total | Control Mean | Control SD | Control Total | Weight | Mean Difference IV, Random, 95% CI |
|---|---|---|---|---|---|---|---|---|
| Fu 2020 | 0.72 | 0.21 | 32 | 1.06 | 0.24 | 33 | 26.0% | -0.34 [-0.45, -0.23] |
| Fu XX 2020 | 2.72 | 0.25 | 37 | 3.86 | 0.33 | 36 | 25.8% | -1.14 [-1.27, -1.01] |
| Yang MB 2020 | 0.12 | 0.33 | 26 | 1 | 0.8 | 23 | 21.8% | -0.88 [-1.23, -0.53] |
| Yu P 2020 | 0.78 | 0.25 | 147 | 1.12 | 0.28 | 148 | 26.4% | -0.34 [-0.40, -0.28] |
| **Total (95% CI)** | | | **242** | | | **240** | **100.0%** | **-0.66 [-1.05, -0.28]** |

Heterogeneity: Tau² = 0.14; Chi² = 123.26, df = 3 (P < 0.00001); I² = 98%
Test for overall effect: Z = 3.40 (P = 0.0007)

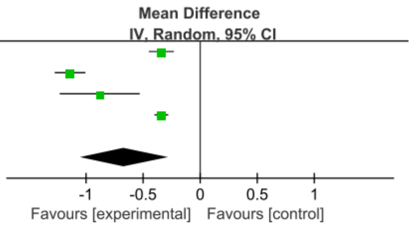


Fig.7f TCM symptom score of fatigue

Fig.7. Clinical symptoms of fever, cough and fatigue. 7a Number of fever reduction cases. 7b TCM symptom score of fever. 7c Number of cough reduction cases. 7d TCM symptom score of cough. 7e Number of fatigue reduction cases. 7f TCM symptom score of fatigue.

Figure

Click here to access/download;Figure;Fig.8. Inflammatory biomarkers..pdf

| Study or Subgroup | Experimental Mean | SD | Total | Control Mean | SD | Total | Weight | Mean Difference IV, Fixed, 95% CI |
|---|---|---|---|---|---|---|---|---|
| Fu 2020 | 5.9 | 0.32 | 32 | 5.54 | 0.34 | 33 | 17.1% | 0.36 [0.20, 0.52] |
| Fu XX 2020 | 5.9 | 0.36 | 37 | 5.64 | 0.37 | 36 | 15.7% | 0.26 [0.09, 0.43] |
| Yu P 2020 | 5.87 | 0.36 | 147 | 5.46 | 0.35 | 148 | 67.0% | 0.41 [0.33, 0.49] |
| Zhang CT 2020 | 6.77 | 2.32 | 22 | 7.02 | 3.1 | 23 | 0.2% | -0.25 [-1.85, 1.35] |
| **Total (95% CI)** | | | **238** | | | **240** | **100.0%** | **0.38 [0.31, 0.44]** |

Heterogeneity: Chi² = 3.15, df = 3 (P = 0.37); I² = 5%
Test for overall effect: Z = 11.13 (P < 0.00001)

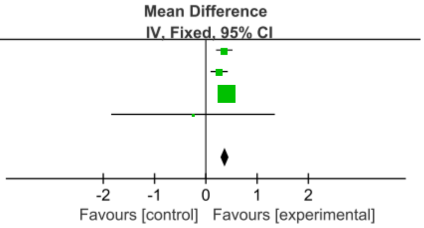


Fig.8a WBC count

| Study or Subgroup | Experimental Mean | SD | Total | Control Mean | SD | Total | Weight | Mean Difference IV, Random, 95% CI |
|---|---|---|---|---|---|---|---|---|
| Fu 2020 | 1.84 | 0.11 | 32 | 1.58 | 0.12 | 33 | 33.1% | 0.26 [0.20, 0.32] |
| Fu XX 2020 | 1.97 | 0.16 | 37 | 1.52 | 0.11 | 36 | 32.9% | 0.45 [0.39, 0.51] |
| Yang MB 2020 | 2.42 | 5.39 | 26 | 3.39 | 6.38 | 23 | 0.4% | -0.97 [-4.30, 2.36] |
| Yu P 2020 | 1.68 | 0.15 | 147 | 1.59 | 0.18 | 148 | 33.6% | 0.09 [0.05, 0.13] |
| **Total (95% CI)** | | | **242** | | | **240** | **100.0%** | **0.26 [0.05, 0.47]** |

Heterogeneity: Tau² = 0.03; Chi² = 98.13, df = 3 (P < 0.00001); I² = 97%
Test for overall effect: Z = 2.48 (P = 0.01)

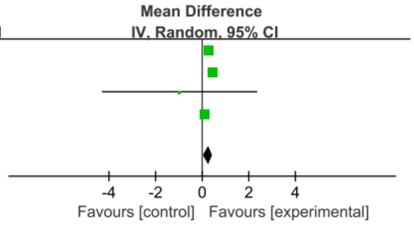


Fig.8b LYM count

| Study or Subgroup | Experimental Mean | SD | Total | Control Mean | SD | Total | Weight | Mean Difference IV, Random, 95% CI |
|---|---|---|---|---|---|---|---|---|
| Fu 2020 | 34.03 | 2 | 32 | 28.85 | 2.4 | 33 | 38.4% | 5.18 [4.11, 6.25] |
| Fu XX 2020 | 32.03 | 2 | 37 | 28.85 | 2.4 | 36 | 38.6% | 3.18 [2.17, 4.19] |
| Zhang CT 2020 | 37.2 | 8.63 | 22 | 22.3 | 6.45 | 23 | 23.0% | 14.90 [10.43, 19.37] |
| **Total (95% CI)** | | | **91** | | | **92** | **100.0%** | **6.65 [3.36, 9.94]** |

Heterogeneity: Tau² = 7.05; Chi² = 28.81, df = 2 (P < 0.00001); I² = 93%
Test for overall effect: Z = 3.96 (P < 0.0001)

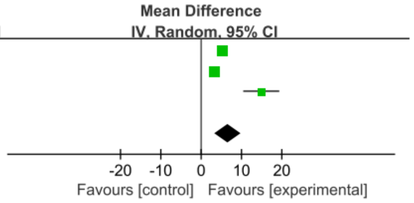


Fig.8c LYM percentage

| Study or Subgroup | Experimental Mean | SD | Total | Control Mean | SD | Total | Weight | Mean Difference IV, Fixed, 95% CI |
|---|---|---|---|---|---|---|---|---|
| Fu 2020 | 55.48 | 2.01 | 32 | 60.06 | 2.98 | 33 | 94.5% | -4.58 [-5.81, -3.35] |
| Yang MB 2020 | 61.12 | 6.61 | 26 | 65.31 | 10.86 | 23 | 5.5% | -4.19 [-9.30, 0.92] |
| **Total (95% CI)** | | | **58** | | | **56** | **100.0%** | **-4.56 [-5.76, -3.36]** |

Heterogeneity: Chi² = 0.02, df = 1 (P = 0.88); I² = 0%
Test for overall effect: Z = 7.46 (P < 0.00001)

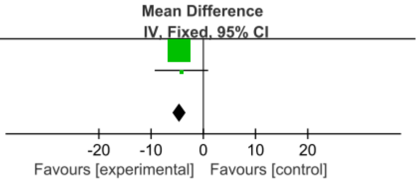


Fig.8d NEU percentage

| Study or Subgroup | Experimental Mean | SD | Total | Control Mean | SD | Total | Weight | Mean Difference IV, Random, 95% CI |
|---|---|---|---|---|---|---|---|---|
| Fu 2020 | 22.75 | 4.8 | 32 | 31.86 | 5.1 | 33 | 17.0% | -9.11 [-11.52, -6.70] |
| Fu XX 2020 | 24.75 | 4.8 | 37 | 32.86 | 5.2 | 36 | 17.2% | -8.11 [-10.41, -5.81] |
| Yang MB 2020 | 2.91 | 3.02 | 26 | 9.73 | 17.78 | 23 | 8.1% | -6.82 [-14.18, 0.54] |
| Yu P 2020 | 22.37 | 4.37 | 147 | 24.37 | 4.37 | 148 | 19.1% | -2.00 [-3.00, -1.00] |
| Zhang CT 2020 | 8.35 | 1.03 | 22 | 15.23 | 2.65 | 23 | 19.0% | -6.88 [-8.05, -5.71] |
| Zhou WM 2020 | 1.9 | 0.85 | 52 | 3.26 | 1.63 | 52 | 19.5% | -1.36 [-1.86, -0.86] |
| **Total (95% CI)** | | | **316** | | | **315** | **100.0%** | **-5.46 [-8.19, -2.72]** |

Heterogeneity: Tau² = 9.89; Chi² = 128.31, df = 5 (P < 0.00001); I² = 96%
Test for overall effect: Z = 3.91 (P < 0.0001)

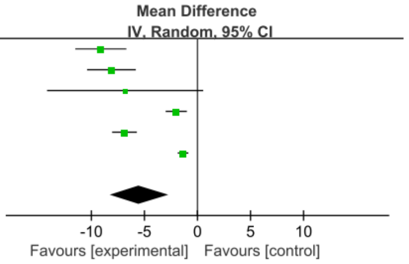


Fig.8e CRP

Fig.8. Inflammatory biomarkers. 8a WBC count. 8b LYM count. 8c LYM percentage. 8d NEU percentage. 8e CRP.

Figure
Click here to access/download;Figure;Fig.9. Adverse effects..pdf

| Study or Subgroup | Experimental Events | Experimental Total | Control Events | Control Total | Weight | Risk Ratio M-H, Random, 95% CI |
|---|---|---|---|---|---|---|
| Duan C 2020 | 27 | 82 | 0 | 41 | 8.8% | 27.83 [1.74, 445.11] |
| Hu F 2020 | 1 | 57 | 0 | 71 | 7.0% | 3.72 [0.15, 89.72] |
| Hu K.2020 | 65 | 142 | 77 | 142 | 43.3% | 0.84 [0.67, 1.07] |
| Zhang YL 2020 | 1 | 80 | 0 | 40 | 7.0% | 1.52 [0.06, 36.46] |
| Zhou WM 2020 | 8 | 52 | 15 | 52 | 34.0% | 0.53 [0.25, 1.15] |
| **Total (95% CI)** | | **413** | | **346** | **100.0%** | **1.13 [0.45, 2.83]** |
| Total events | 102 | | 92 | | | |

Heterogeneity: Tau² = 0.49; Chi² = 10.95, df = 4 (P = 0.03); I² = 63%
Test for overall effect: Z = 0.27 (P = 0.79)

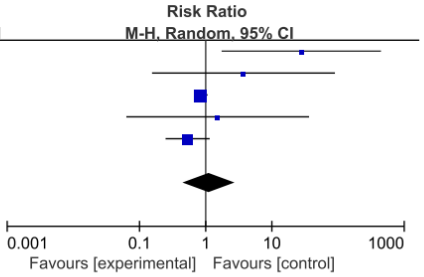


Fig.9a Number of adverse effects cases

| Study or Subgroup | Experimental Events | Experimental Total | Control Events | Control Total | Weight | Risk Ratio M-H, Fixed, 95% CI |
|---|---|---|---|---|---|---|
| Hu K.2020 | 8 | 142 | 8 | 142 | 72.7% | 1.00 [0.39, 2.59] |
| Zhou WM 2020 | 4 | 52 | 3 | 52 | 27.3% | 1.33 [0.31, 5.67] |
| **Total (95% CI)** | | **194** | | **194** | **100.0%** | **1.09 [0.49, 2.41]** |
| Total events | 12 | | 11 | | | |

Heterogeneity: Chi² = 0.11, df = 1 (P = 0.74); I² = 0%
Test for overall effect: Z = 0.21 (P = 0.83)

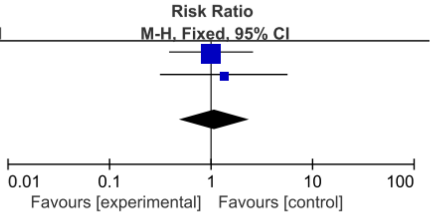


Fig.9b Number of nausea and vomiting cases

| Study or Subgroup | Experimental Events | Experimental Total | Control Events | Control Total | Weight | Risk Ratio M-H, Random, 95% CI |
|---|---|---|---|---|---|---|
| Duan C 2020 | 27 | 82 | 0 | 41 | 16.6% | 27.83 [1.74, 445.11] |
| Hu F 2020 | 1 | 57 | 0 | 71 | 14.3% | 3.72 [0.15, 89.72] |
| Hu K.2020 | 8 | 142 | 19 | 142 | 29.9% | 0.42 [0.19, 0.93] |
| Zhang CT 2020 | 1 | 80 | 0 | 40 | 14.4% | 1.52 [0.06, 36.46] |
| Zhou WM 2020 | 3 | 52 | 3 | 52 | 24.8% | 1.00 [0.21, 4.73] |
| **Total (95% CI)** | | **413** | | **346** | **100.0%** | **1.72 [0.34, 8.67]** |
| Total events | 40 | | 22 | | | |

Heterogeneity: Tau² = 2.12; Chi² = 13.44, df = 4 (P = 0.009); I² = 70%
Test for overall effect: Z = 0.65 (P = 0.51)

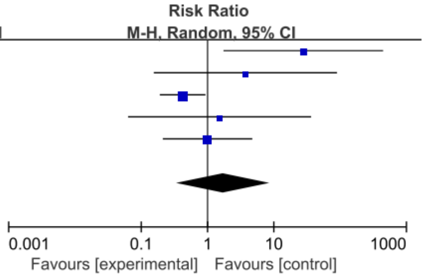


Fig.9c Number of diarrhea cases

| Study or Subgroup | Experimental Events | Experimental Total | Control Events | Control Total | Weight | Risk Ratio M-H, Random, 95% CI |
|---|---|---|---|---|---|---|
| Hu K.2020 | 32 | 142 | 32 | 142 | 59.9% | 1.00 [0.65, 1.54] |
| Zhou WM 2020 | 1 | 52 | 9 | 52 | 40.1% | 0.11 [0.01, 0.85] |
| **Total (95% CI)** | | **194** | | **194** | **100.0%** | **0.41 [0.05, 3.69]** |
| Total events | 33 | | 41 | | | |

Heterogeneity: Tau² = 2.03; Chi² = 4.63, df = 1 (P = 0.03); I² = 78%
Test for overall effect: Z = 0.79 (P = 0.43)

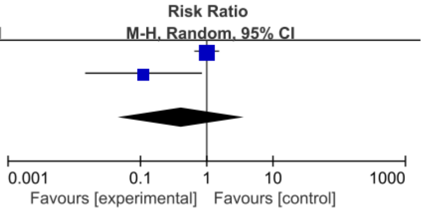


Fig.9d Number of abnormal liver function cases

Fig.9. Adverse effects. 9a Number of adverse effects cases. 9b Number of nausea and vomiting cases. 9c Number of diarrhea cases. 9d Number of abnormal liver function cases.

Other Click here to access/download;Figure;Table 1 Characteristics of included RCTs..pdf

Table 1
Characteristics of included RCTs.

| First author | Type of COVID-19 | Sample size (M/F) | Age (years) | Intervention | Control | Duration | Outcome measures |
|---|---|---|---|---|---|---|---|
| Duan C [10] | mild | T:82(39/43) C:41(23/18) | T:51.99±13.88 C:50.29±13.17 | Jinhua Qinggan granule + CWM | CWM including antiviral, anti-infection, and symptomatic therapies | 5 days | Clinical symptoms, and adverse effects |
| Fu [11] | mild/ moderate | T:32(17/15) C:33(19/14) | T:43.26±7.15 C:43.68±6.45 | Toujie Quwen granule + CWM | CWM including abidor tablets, moxifloxacin tablets, and ambroxol tablets | 10 days | Lung CT, clinical cure rate, rate of conversion to severe cases, clinical symptoms, inflammatory biomarkers, and adverse effects |
| Fu XX [12] | moderate | T:37(19/18) C:36(19/17) | T:45.26±7.25 C:44.68±7.45 | Toujie Quwen granule + CWM | CWM including abidor tablets, and ambroxol tablets | 15 days | Clinical cure rate, rate of conversion to severe cases, clinical symptoms, inflammatory biomarkers, and adverse effects |
| Hu F [13] | moderate | T:100(49/51) C:100(55/45) | T:47.00±14.06 C:49.28±11.14 | Jinyinhua oral liquid + CWM | CWM including interferon-α, lopinavir and tonavir tablets, symptomatic and supportive therapies | 10 days | Lung CT, virus nucleic acid testing, rate of conversion to severe cases, and adverse effects |
| Hu K [14] | mild/ moderate | T:142(79/63) C:142(71/71) | T:50.4±15.2 C:51.8±14.8 | Lianhua Qingwen capsule + CWM | CWM including oxygen therapy, antiviral, and symptomatic therapies | 14 days | Lung CT, clinical cure rate, virus nucleic acid testing, rate of conversion to severe cases, clinical symptoms, and adverse effects |
| Qiu M [15] | moderate | T:25(13/12) C:25(14/11) | T:53.35±18.35 C:51.32±14.62 | Maxing Xuanfei Jiedu Decoction + CWM | CWM including interferon-α, lopinavir and tonavir tablets | 10 days | Lung CT, rate of conversion to severe cases, and clinical symptoms |

| | | | | | | | |
|---|---|---|---|---|---|---|---|
| Sun HM [16] | mild/ moderate | T:32(17/15) C:25(11/14) | T:45.4±14.10 C:42.0±11.70 | Lianhua Qingke granule + CWM | CWM including interferon-α, lopinavir and tonavir tablets, symptomatic and supportive therapies | 14days | Lung CT, rate of conversion to severe cases, and clinical symptoms |
| Yang MB [17] | moderate | T:26(16/10) C:23(9/14) | T:50.35±13.37 C:47.17±16.57 | Reyanning mixture + CWM | CWM including interferon-α, lopinavir and tonavir tablets, abidor tablets, and ribavirin | 7 days | Virus nucleic acid testing, rate of conversion to severe cases, clinical symptoms, inflammatory biomarkers, and adverse effects |
| Yu P [18] | mild/ moderate | T:147(82/65) C:148(89/59) | T:48.27±9.56 C:47.25±8.67 | Lianhua Qingwen granule+ CWM | CWM including abidor tablets, moxifloxacin tablets, and ambroxol tablets | 7 days | Lung CT, clinical cure rate, rate of conversion to severe cases, clinical symptoms, inflammatory biomarkers, and adverse effects |
| Zhang CT [19] | moderate | T: 22 (9/ 13) C: 23 (10/13) | T: 53.7 ± 3.5 C: 55.6 ± 4.2 | Jiawei Dayuan Decoction + CWM | CWM including oxygen therapy, antivirus, and symptomatic therapies | 7 days | Lung CT, clinical symptoms, inflammatory biomarkers, and adverse effects |
| Zhang YL [20] | moderate | T: 80 (50/ 30) C: 40(23/17) | T: 53.4±13.70 C:52.0±14.10 | Jinyinhua oral liquid + CWM | CWM including interferon-α, lopinavir and tonavir tablets, symptomatic and supportive therapies | 10 days | Rate of conversion to severe cases, clinical symptoms, and adverse effects |
| Zhou WM [21] | moderate | T: 52 (32/ 20) C: 52(28/24) | T: 52.47±10.99 C:51.11±9.87 | diammonium glycyrrhizinate + CWM | CWM including lopinavir and tonavir tablets, symptomatic and supportive therapies | 14 days | Clinical cure rate, inflammatory biomarkers, and adverse effects |

Click here to access/download
**Supporting Information**
S1 Checklist. PRISMA 2009 checklist..doc
