# Supplementary material for: Add-on effect of Chinese herbal medicine in the treatment of mild to moderate COVID-19: A systematic review and meta-analysis
Source: PLoS One. 2021 Aug 20;16(8):e0256429. doi: 10.1371/journal.pone.0256429 (PMC8378756; doi:10.1371/journal.pone.0256429)
Supplement: Attachment — Submitted filename: Response to Reviewers.pdf [file pone.0256429.s007.pdf]

Response to Reviewers

Reviewer #1: kindly, find the primary review and comments in the attached Pdf file, in addition, please pay attention to the following points:
-The main claim of the paper is clear and significant, specially in such unprecedent situation.

-The analysis of data supports the claim of the paper, however; it would be better to connect this study with more previous published data and literatures in a way that reduce duplication and support the findings of this paper.
Response: in the discussion section, this review has linked this study with more previously published data and literature for analysis.
-a more detailed protocol of the statistical analysis is needed especially, most of the data used in the analysis has been retrieved from papers in Chinese language.
Response: in our review, a more detailed protocol of the statistical analysis was developed. Trials on Chinese herbal medicine for mild to moderate COVID-19 were conducted in mainland China. Most of the trials were published online in Chinese. Therefore, most of the data used in the analysis has been retrieved from papers in Chinese language.
-Type of samples in treatment and control groups doesn't exclude the possibility of synergistic/ combination effect between CHM and western medicine. have you had any studies that used CHM only on separate groups as a treatment? Was there any control group that didn't receive any treatment? is there any information about hospitalization or receiving any other special care(ex. ventilator) beside the treatment?
Response: trials of Chinese herbal medicine in the treatment of mild to moderate COVID-19 were included in this review. The treatment group was treated with Chinese herbal medicine combined with conventional therapy. No trials that used CHM only on separate groups as a treatment. There was no control group that did not receive any treatment. Since the participants were diagnosed as mild to moderate COVID-19, patients did not receive ventilator treatment. The specific treatment information is listed in Table 1.
i.e: we can't conclude for sure the CHM as a separate, effective, and safe treatment for mild to moderate COVID-19.

Response: the conclusion of this review is that Chinese herbal medicine combined with conventional therapy could be effective and safe in the treatment of adults with mild to moderate COVID-19.

Reviewer #2: Valuable data was provided in this manuscript, which are not easily assessible for international readers outside China. Hence, I have to stress that this manuscript presents precious and valuable data that will benefit the literature and improve understanding of the role of TCM in COVID-19. However, in general, I find that there is lack of clarity in definition of many things including outcome measures and treatment groups. Importantly, the discussion was superficial. There needs to be correlation between ROB, quality of study, heterogeneity and interpretation of results.

Please find my suggestion as below and as specify in the attachment:

1. Strongly suggest for professional language/ scientific proof-reading to correct grammar, sentence structuring, and selection of words that are preferred to represent precise scientific writing for the entire manuscript. Kindly check for the use of oxford comma and appropriate/excessive use of connective words throughout. The authors in particular like to start sentences with the word "And". Spacing between words and symbols needs to be checked and made consistent.
Response: grammar, sentence structure, comma, and connective words have been corrected.
2. The eligibility criteria can be rewritten as inclusion and exclusion criteria clearly; or rearrange with clearer subtopics differentiation. The different levels of the subtopics in the methods needs to be clear. For example (here I am using numbers to explain an example of how the different levels needs to be clarified. It is to the authors discretion on presenting this without the numbers)
Response: the eligibility criteria have been rewritten as inclusion and exclusion criteria.
3. Specific to the methods
a. Kindly check against the PRISMA checklist- Present full electronic search strategy for at least one database (please present the combination of keywords used); Describe method of data extraction from reports (e.g., piloted forms, independently, in duplicate) and any processes for obtaining and confirming data from investigators (kindly mention if attempts were made to seek for additional data)

Response: the PubMed search strategy is listed. The method of data extraction from reports, and any processes for obtaining and confirming data from investigators were described in this review.
b. Clarify inclusion criteria- oral Chinese herbal medicine only

Response: inclusion criteria have been clarified - oral Chinese herbal medicine only.
c. Outcome measures need to be well defined e.g. what is clinical cure rate, what is effective rate of lung CT
Response: Outcome measures (e.g. clinical cure rate, lung CT) have been well defined.
4. Results
a. arrange the level of subheadings accordingly as suggested for methods

Response: the level of subheadings has been arranged accordingly as suggested for methods.
b. definition of CHM and CWM needs to be clear- the naming of the groups. Although it is mentioned that CHM group received both herbal and western medicine in methods, CHM is still abbreviated as chinese herbal medicine. The results are mostly written as 'the outcomes are better with treatment by CHM', which can be confusing to interpret, and easily misunderstood as if CHM solely (without western medicine) is beneficial. Suggest to clearly describe what each group means with distinct abbreviations for groups. Perhaps it is also because of the choice of word 'by' which when read, is interpreted this way, hence consider rewriting the results section with more precise

selection of words.

Response: the naming of the groups has been rewritten.

5. Discussion

Although an interesting topic with very valuable data (I cannot emphasize this enough, this is very valuable data), the discussion is superficial and lacked depth. few suggestion of topics to discuss include

- heterogeneity of the studies and the impact on the findings.
- impact of different formulations used and how did the authors came to collectively interpreting them in the same meta-analyses (also consider that different herbs would have acted differently, and certainly herb-herb interaction should be discussed)
- risk of bias and how that affects results interpretation
- discuss on adverse events, reporting bias?
- quality of herbal intervention used
- suggest to consider consort checklist for tcm to evaluate quality of reporting which can further strengthen discussion
- how does this new information applies to the global scenario and what are the challenges of applying TCM in this scenario
- difference between TCM approach (Which is based on individualised assessment, and can be even affected by factors such as diet, body type, environment, geographical location, weather) and western medicine approach
- it is also important to point out that the concept of selecting treatment based on TCM philosophy is vastly different. My own personal experience consulting TCM experts from China , which I quote him, the treatment in China (Wuhan experiencing winter that time) may not suit for countries with different climate and weather (e.g. a Southeast Asian country with hot and humid climate, with different diet practices)
- also consider that herbs, in raw form, extracted, or in different extraction medium in phytochemistry context would yield different phytocompounds, and one of the main gap here is a lack of consistency/ documentation/ quantitation/ interpretation of what is the mechanisms and bioactive compound involved
- regulatory challenges
- contribution of confounding factors such as co-morbidities, differences in western medicine used

Response: in our review, the suggestions on the above topics have been incorporated into the discussion.

6. Conclusion

The conclusion partly answers the objective. However, critical appraisal (as mentioned in the discussion section) would help interpret the results better and make it more relevant to the global scenario. The limitations are not only to conducting high quality studies (to which quality of studies were not actually evaluated and discussed in the discussion section), but application to the world, and consideration of knowledge gap.

Response: critical appraisal has been made.

7. Is the western medicine arm treatment really identical? There is no data available on what is given as western medicine and difficult to decide if they are identical, similar, or if they actually can be a confounding factor.

Response: the western medicine arm treatment really is not identical in different trials. Specific treatment information is listed in Table 1.

8. It would be good to at least describe what are the different composition of the common TCM formulations used.

Response: the different components of TCM were described in this review.

But overall, I am very appreciative that this data will be made available and I look forward to the amended version. Again, I cannot emphasize enough how valuable these data are.

Reviewer #3: Reviewer's Comments

Chinese herbal medicine in adults with mild to moderate coronavirus disease 2019(COVID-19): A systematic review and meta-analysis with MS ID PONE-D-20-38124.

Major Comments

1. Meta-analytical studies have been carried out majorly on the basis of ref 10-20 and all of them are published in Chinese journals except ref 14 only, which indicates towards the biasness of choice of content used for carrying out the study. Authors are recommended to refer the content from other sources as well to further validate the findings.

Response: trials of Chinese herbal medicine in the treatment of mild to moderate COVID-19 were comprehensively searched in eight electronic databases. Potentially eligible data was obtained by manually searching the reference list of previously published reviews. If possible, the conference abstracts were reviewed to find unpublished trials, and the data was obtained by contacting the author.

2. COVID-19 data provided in introduction section is contradictory with WHO data. Authors are suggested to cross-check the COVID-19 count provided on WHO website.

Response: COVID-19 data was cross-checked according to WHO website.

3. Conclusion of study is not in accordance with results therefore needs to be modified accordingly.

Response: conclusion of our study was modified in accordance with results.

4. Manuscript mandatorily needs to be handled by language experts as there exists several ambiguities in its current form.

Response: our manuscript was handled by language experts.

Minor Comments

1. Abbreviations are missing throughout the manuscript.

Response: abbreviations full names were listed in the manuscript.

2. Cross-check the format of references to maintain homogeneity.

Response: the format of references was cross-checked.

Reviewer #4: This is a very important review to publish at this time. These findings are very relevant and contribute to the essential knowledge about a globally crippling disease. The review was performed with rigorous standards and therefore the results can contribute significantly to the prevention and treatment of COVID-19. Thank you for your work.
