# Supplementary material for: Add-on effect of Chinese herbal medicine in the treatment of mild to moderate COVID-19: A systematic review and meta-analysis
Source: PLoS One. 2021 Aug 20;16(8):e0256429. doi: 10.1371/journal.pone.0256429 (PMC8378756; doi:10.1371/journal.pone.0256429)
Supplement: Attachment — Submitted filename: PONE-D-20-38124_R1_reviewer - Copy - Copy.pdf [file pone.0256429.s008.pdf]

PLOS ONE

Chinese herbal medicine in adults with mild to moderate COVID-19: A systematic review and meta-analysis

--Manuscript Draft--

**Manuscript Number:**	PONE-D-20-38124R1
**Article Type:**	Research Article
**Full Title:**	Chinese herbal medicine in adults with mild to moderate COVID-19: A systematic review and meta-analysis
**Short Title:**	A systematic review and meta-analysis
**Corresponding Author:**	Lipeng Shi<br>Traditional Chinese Medicine Hospital of Dianjiang Chongqing<br>Chongqing, CHINA
**Keywords:**	Coronavirus disease 2019, Chinese herbal medicine, Systematic review, Meta-analysis, Randomized controlled trials
**Abstract:**	Introduction<br><br>Coronavirus disease 2019 (COVID-19) caused by severe acute respiratory syndrome coronavirus 2 (SARS-CoV-2) has spread all over the world, which is a serious threat to human life and health. In China's experience in fighting COVID-19, traditional Chinese medicine (TCM), especially Chinese herbal medicine (CHM), has played an important role. Human studies reported the beneficial effects of CHM in the treatment of adult patients with mild to moderate COVID-19. Presently there is no systematic evaluation of the clinical efficacy of CHM in adult patients with mild to moderate COVID-19. Therefore, this review was designed to evaluate the efficacy and safety of CHM in the treatment of adult patients with mild to moderate COVID-19.Methods<br><br>Randomized controlled trials (RCTs) on Chinese herbal medicine for mild to moderate COVID-19 were searched in the following eight electronic databases: PubMed, EMBASE, Cochrane Central Register of Controlled trials, the Clinical Trials.gov website, China National Knowledge Infrastructure (CNKI), China Science and Technology Journal Database (VIP), Wanfang Database and China Biology Medicine (CBM) from December 2019 to November 2020. Two reviewers independently searched, selected studies, and extracted data according to the eligibility criteria. Cochrane Risk of Bias (ROB) tool was used to assess the methodological quality of the included RCTs. Revman 5.3.0 software was used for statistical analysis.Results<br><br>Twelve eligible RCTs were included with a total sample size of 1393. Our meta-analyses found that lung CT [RR=1.26, 95%CI (1.15, 1.38), P < 0.00001], and clinical cure rate [RR=1.26, 95%CI (1.16, 1.38), P < 0.00001] of CHM combined with conventional therapy in the treatment of mild to moderate COVID-19 was better than that of conventional therapy. The rate of conversion to severe cases [RR=0.48, 95%CI (0.32, 0.73), P=0.0005], TCM symptom score of fever [MD=-0.62, 95%CI (-0.79, -0.45), P < 0.00001], cough cases [RR=1.43, 95%CI (1.16, 1.75), P=0.0006], TCM symptom score of cough[MD=-1.07, 95%CI (-1.29, -0.85), P < 0.00001], TCM symptom score of fatigue[MD=-0.66, 95%CI (-1.05, -0.28), P=0.0007], and CRP[MD=-5.46, 95%CI (-8.19, -2.72), P < 0.0001] of CHM combined with conventional therapy was significantly lower than that of conventional therapy. The WBC count was significantly higher than that of conventional therapy[MD=0.38, 95%CI (0.31, 0.44), P < 0.00001]. Our meta-analysis results were robust and reliable through sensitivity analysis.Conclusion<br><br>Chinese herbal medicine combined with conventional therapy could be effective and safe in the treatment of adults with mild to moderate COVID-19. More high-quality RCTs are needed in the future.
**Order of Authors:**	Xuqin Du
	Lipeng Shi
	Wenfu Cao

Powered by Editorial Manager® and ProduXion Manager® from Aries Systems Corporation

	Biao Zuo
	Aimin Zhou
**Response to Reviewers:**	Reviewer #1: kindly, find the primary review and comments in the attached Pdf file, in addition, please pay attention to the following points:<br>-The main claim of the paper is clear and significant, specially in such unprecedent situation.<br>-The analysis of data supports the claim of the paper, however; it would be better to connect this study with more previous published data and literatures in a way that reduce duplication and support the findings of this paper.<br><br>Response: in the discussion section, this review has linked this study with more previously published data and literature for analysis.<br>-a more detailed protocol of the statistical analysis is needed especially, most of the data used in the analysis has been retrieved from papers in Chinese language.<br><br>Response: in our review, a detailed protocol of the statistical analysis was developed. Trials on Chinese herbal medicine for mild to moderate COVID-19 were conducted in mainland China. Most of the trials were published online in Chinese. Therefore, most of the data used in the analysis has been retrieved from papers in Chinese language.<br>-Type of samples in treatment and control groups doesn't exclude the possibility of synergistic/ combination effect between CHM and western medicine. have you had any studies that used CHM only on separate groups as a treatment? Was there any control group that didn't receive any treatment? is there any information about hospitalization or receiving any other special care(ex. ventilator) beside the treatment?<br><br>Response: trials of Chinese herbal medicine in the treatment of mild to moderate COVID-19 were included in this review. The treatment group was treated with Chinese herbal medicine combined with conventional therapy. No trials that used CHM only on separate groups as a treatment. There was no control group that did not receive any treatment. Since the participants were diagnosed as mild to moderate COVID-19, patients did not receive ventilator treatment. The specific treatment information is listed in Table 1.<br>i.e: we can't conclude for sure the CHM as a separate, effective, and safe treatment for mild to moderate COVID-19.<br><br>Response: the conclusion of this review is that Chinese herbal medicine combined with conventional therapy could be effective and safe in the treatment of adults with mild to moderate COVID-19.<br>Reviewer #2: Valuable data was provided in this manuscript, which are not easily assessible for international readers outside China. Hence, I have to stress that this manuscript presents precious and valuable data that will benefit the literature and improve understanding of the role of TCM in COVID-19. However, in general, I find that there is lack of clarity in definition of many things including outcome measures and treatment groups. Importantly, the discussion was superficial. There needs to be correlation between ROB, quality of study, heterogeneity and interpretation of results. Please find my suggestion as below and as specify in the attachment:<br><br>1. Strongly suggest for professional language/ scientific proof-reading to correct grammar, sentence structuring, and selection of words that are preferred to represent precise scientific writing for the entire manuscript. Kindly check for the use of oxford comma and appropriate/excessive use of connective words throughout. The authors in particular like to start sentences with the word "And". Spacing between words and symbols needs to be checked and made consistent.<br><br>Response: grammar, sentence structure, comma, and connective words have been corrected.<br>2. The eligibility criteria can be rewritten as inclusion and exclusion criteria clearly; or rearrange with clearer subtopics differentiation. The different levels of the subtopics in the methods needs to be clear. For example (here I am using numbers to explain an example of how the different levels needs to be clarified. It is to the authors discretion on presenting this without the numbers)<br><br>Response: the eligibility criteria have been rewritten as inclusion and exclusion criteria.

Powered by Editorial Manager® and ProduXion Manager® from Aries Systems Corporation

	3. Specific to the methods<br>a. Kindly check against the PRISMA checklist- Present full electronic search strategy for at least one database (please present the combination of keywords used); Describe method of data extraction from reports (e.g., piloted forms, independently, in duplicate) and any processes for obtaining and confirming data from investigators (kindly mention if attempts were made to seek for additional data)<br><br>Response: the PubMed search strategy is listed. The method of data extraction from reports, and any processes for obtaining and confirming data from investigators were described in this review.<br>b. Clarify inclusion criteria- oral Chinese herbal medicine only<br><br>Response: inclusion criteria have been clarified - oral Chinese herbal medicine only.<br>c. Outcome measures need to be well defined e.g. what is clinical cure rate, what is effective rate of lung CT<br><br>Response: Outcome measures (e.g. clinical cure rate, lung CT) have been well defined.<br>4. Results<br>a. arrange the level of subheadings accordingly as suggested for methods<br><br>Response: the level of subheadings has been arranged accordingly as suggested for methods.<br>b. definition of CHM and CWM needs to be clear- the naming of the groups. Although it is mentioned that CHM group received both herbal and western medicine in methods, CHM is still abbreviated as chinese herbal medicine. The results are mostly written as 'the outcomes are better with treatment by CHM', which can be confusing to interpret, and easily misunderstood as if CHM solely (without western medicine) is beneficial. Suggest to clearly describe what each group means with distinct abbreviations for groups. Perhaps it is also because of the choice of word 'by' which when read, is interpreted this way, hence consider rewriting the results section with more precise selection of words.<br><br>Response: the naming of the groups has been rewritten.<br>5. Discussion<br>Although an interesting topic with very valuable data (I cannot emphasize this enough, this is very valuable data), the discussion is superficial and lacked depth. few suggestion of topics to discuss include<br>- heterogeneity of the studies and the impact on the findings.<br>- impact of different formulations used and how did the authors came to collectively interpreting them in the same meta-analyses (also consider that different herbs would have acted differently, and certainly herb-herb interaction should be discussed)<br>- risk of bias and how that affects results interpretation<br>- discuss on adverse events, reporting bias?<br>- quality of herbal intervention used<br>- suggest to consider consort checklist for tcm to evaluate quality of reporting which can further strengthen discussion<br>- how does this new information applies to the global scenario and what are the challenges of applying TCM in this scenario<br>- difference between TCM approach (Which is based on individualised assessment, and can be even affected by factors such as diet, body type, environment, geographical location, weather) and western medicine approach<br>- it is also important to point out that the concept of selecting treatment based on TCM philosophy is vastly different. My own personal experience consulting TCM experts from China , which I quote him, the treatment in China (Wuhan experiencing winter that time) may not suit for countries with different climate and weather (e.g. a Southeast Asian country with hot and humid climate, with different diet practices)<br>- also consider that herbs, in raw form, extracted, or in different extraction medium in phytochemistry context would yield different phytocompounds, and one of the main gap here is a lack of consistency/ documentation/ quantitation/ interpretation of what is the mechanisms and bioactive compound involved<br>- regulatory challenges<br>- contribution of confounding factors such as co-morbidities, differences in western medicine used

Response: in our review, the suggestions on the above topics have been incorporated into the discussion.
6. Conclusion
The conclusion partly answers the objective. However, critical appraisal (as mentioned in the discussion section) would help interpret the results better and make it more relevant to the global scenario. The limitations are not only to conducting high quality studies (to which quality of studies were not actually evaluated and discussed in the discussion section), but application to the world, and consideration of knowledge gap.

Response: critical appraisal has been made.
7. Is the western medicine arm treatment really identical? There is no data available on what is given as western medicine and difficult to decide if they are identical, similar, or if they actually can be a confounding factor.

Response: the western medicine arm treatment really is not identical in different trials. Specific treatment information is listed in Table 1.
8. It would be good to at least describe what are the different composition of the common TCM formulations used.

Response: the different components of TCM were described in this review.
But overall, I am very appreciative that this data will be made available and I look forward to the amended version. Again, I cannot emphasize enough how valuable these data are.
Reviewer #3: Reviewer’s Comments
Chinese herbal medicine in adults with mild to moderate coronavirus disease 2019(COVID-19): A systematic review and meta-analysis with MS ID PONE-D-20-38124.
Major Comments
1. Meta-analytical studies have been carried out majorly on the basis of ref 10-20 and all of them are published in Chinese journals except ref 14 only, which indicates towards the biasness of choice of content used for carrying out the study. Authors are recommended to refer the content from other sources as well to further validate the findings.

Response: trials of Chinese herbal medicine in the treatment of mild to moderate COVID-19 were comprehensively searched in eight electronic databases. Potentially eligible data was obtained by manually searching the reference list of previously published reviews. If possible, the conference abstracts were reviewed to find unpublished trials, and the data was obtained by contacting the author.
2. COVID-19 data provided in introduction section is contradictory with WHO data. Authors are suggested to cross-check the COVID-19 count provided on WHO website.

Response: COVID-19 data was cross-checked according to WHO website.
3. Conclusion of study is not in accordance with results therefore needs to be modified accordingly.

Response: conclusion of our study was modified in accordance with results.
4. Manuscript mandatorily needs to be handled by language experts as there exists several ambiguities in its current form.

Response: our manuscript was handled by language experts.
Minor Comments
1. Abbreviations are missing throughout the manuscript.

Response: abbreviations full names were listed in the manuscript.
2. Cross-check the format of references to maintain homogeneity.

Response: the format of references was cross-checked.
Reviewer #4: This is a very important review to publish at this time. These findings are very relevant and contribute to the essential knowledge about a globally crippling disease. The review was performed with rigorous standards and therefore the results can contribute significantly to the prevention and treatment of COVID-19. Thank you for your work.

Additional Information:	
**Question**	**Response**
**Financial Disclosure**<br><br>Enter a financial disclosure statement that describes the sources of funding for the work included in this submission. Review the submission guidelines for detailed requirements. View published research articles from *PLOS ONE* for specific examples.<br><br>This statement is required for submission and **will appear in the published article** if the submission is accepted. Please make sure it is accurate.<br><br>**Unfunded studies**<br>Enter: *The author(s) received no specific funding for this work.*<br><br>**Funded studies**<br>Enter a statement with the following details:<br>• Initials of the authors who received each award<br>• Grant numbers awarded to each author<br>• The full name of each funder<br>• URL of each funder website<br>• Did the sponsors or funders play any role in the study design, data collection and analysis, decision to publish, or preparation of the manuscript?<br>• **NO** - Include this sentence at the end of your statement: *The funders had no role in study design, data collection and analysis, decision to publish, or preparation of the manuscript.*<br>• **YES** - Specify the role(s) played.<br><br>* typeset	This study was funded by the National Natural Science Foundation of China (No.81573860) and the postdoctoral research project of Chongqing Medical University (No. R11004). The funders had no role in study design, data collection and analysis, decision to publish, or preparation of the manuscript.
**Competing Interests**<br><br>Use the instructions below to enter a competing interest statement for this submission. On behalf of all authors, disclose any competing interests that could be perceived to bias this work—acknowledging all financial support and any other relevant financial or non-financial competing interests.	The authors declare that they have no competing interests.

Powered by Editorial Manager® and ProduXion Manager® from Aries Systems Corporation

This statement **will appear in the published article** if the submission is accepted. Please make sure it is accurate. View published research articles from *PLOS ONE* for specific examples.<br><br>**NO authors have competing interests**<br><br>Enter: *The authors have declared that no competing interests exist.*<br><br>**Authors with competing interests**<br><br>Enter competing interest details beginning with this statement:<br><br>*I have read the journal's policy and the authors of this manuscript have the following competing interests: [insert competing interests here]*<br><br>* typeset	
**Ethics Statement**<br><br>Enter an ethics statement for this submission. This statement is required if the study involved:<br><br>• Human participants<br>• Human specimens or tissue<br>• Vertebrate animals or cephalopods<br>• Vertebrate embryos or tissues<br>• Field research<br><br>Write "N/A" if the submission does not require an ethics statement.<br><br>General guidance is provided below. Consult the submission guidelines for detailed instructions. **Make sure that all information entered here is included in the Methods section of the manuscript.**	Ethical approval and patient consent are not required since this is an overview based on published studies.

**Format for specific study types**<br><br>**Human Subject Research (involving human participants and/or tissue)**<br>• Give the name of the institutional review board or ethics committee that approved the study<br>• Include the approval number and/or a statement indicating approval of this research<br>• Indicate the form of consent obtained (written/oral) or the reason that consent was not obtained (e.g. the data were analyzed anonymously)<br><br>**Animal Research (involving vertebrate animals, embryos or tissues)**<br>• Provide the name of the Institutional Animal Care and Use Committee (IACUC) or other relevant ethics board that reviewed the study protocol, and indicate whether they approved this research or granted a formal waiver of ethical approval<br>• Include an approval number if one was obtained<br>• If the study involved *non-human primates*, add *additional details* about animal welfare and steps taken to ameliorate suffering<br>• If anesthesia, euthanasia, or any kind of animal sacrifice is part of the study, include briefly which substances and/or methods were applied<br><br>**Field Research**<br><br>Include the following details if this study involves the collection of plant, animal, or other materials from a natural setting:<br>• Field permit number<br>• Name of the institution or relevant body that granted permission	
**Data Availability**<br><br>Authors are required to make all data underlying the findings described fully available, without restriction, and from the time of publication. PLOS allows rare exceptions to address legal and ethical concerns. See the PLOS Data Policy and FAQ for detailed information.	No - some restrictions will apply

A Data Availability Statement describing where the data can be found is required at submission. Your answers to this question constitute the Data Availability Statement and **will be published in the article**, if accepted.<br><br>**Important:** Stating 'data available on request from the author' is not sufficient. If your data are only available upon request, select 'No' for the first question and explain your exceptional situation in the text box.<br><br>Do the authors confirm that all data underlying the findings described in their manuscript are fully available without restriction?	
**Describe where the data may be found in full sentences. If you are copying our sample text, replace any instances of XXX with the appropriate details.**<br><br>• If the data are **held or will be held in a public repository**, include URLs, accession numbers or DOIs. If this information will only be available after acceptance, indicate this by ticking the box below. For example: *All XXX files are available from the XXX database (accession number(s) XXX, XXX.*).<br>• If the data are all contained **within the manuscript and/or Supporting Information files**, enter the following: *All relevant data are within the manuscript and its Supporting Information files.*<br>• If neither of these applies but you are able to provide **details of access elsewhere**, with or without limitations, please do so. For example:<br><br>*Data cannot be shared publicly because of [XXX]. Data are available from the XXX Institutional Data Access / Ethics Committee (contact via XXX) for researchers who meet the criteria for access to confidential data.*<br><br>*The data underlying the results presented in the study are available from (include the name of the third party*	The data underlying the results presented in the study are available from Supporting Information files.

and contact information or URL).<br>• This text is appropriate if the data are owned by a third party and authors do not have permission to share the data.<br><br>* typeset	
Additional data availability information:	

Manuscript Click here to access/download;Manuscript;Manuscript.doc

Chinese herbal medicine in adults with mild to moderate COVID-19: A systematic review and meta-analysis

Xuqin Du[1,2], Lipeng Shi [3,*], Wenfu Cao [1,2,4,*] , Biao Zuo[1,2], Aimin Zhou[3]

[1] College of Traditional Chinese Medicine, Chongqing Medical University, Chongqing 400016, People's Republic of China

[2] Chongqing Key Laboratory of Traditional Chinese Medicine for Prevention and Cure of Metabolic Diseases, Chongqing 400016, People's Republic of China

[3] Department of Cardiovascular Unit, Traditional Chinese medicine hospital Dianjiang Chongqing, Chongqing 408300, People's Republic of China

[4] Department of Chinese Traditional Medicine, The First Affiliated Hospital of Chongqing Medical University, Chongqing 400016, People's Republic of China

* Corresponding author

E-mail: shilipeng0206@163.com(LPS); caowenfu9316@163.com(WFC)

Abstract

Introduction

Coronavirus disease 2019 (COVID-19) caused by severe acute respiratory syndrome coronavirus 2 (SARS-CoV-2) has spread all over the world, which is a serious threat to human life and health. In China's experience in fighting COVID-19, traditional Chinese medicine (TCM), especially Chinese herbal medicine (CHM), has played an important role. Human studies reported the beneficial effects of CHM in the treatment of adult patients with mild to moderate COVID-19. Presently there is no systematic evaluation of the clinical efficacy of CHM in adult patients with mild to moderate COVID-19. Therefore, this review was designed to evaluate the efficacy and safety of CHM in the treatment of adult patients with mild to moderate COVID-19.

Methods

Randomized controlled trials (RCTs) on Chinese herbal medicine for mild to moderate COVID-19 were searched in the following eight electronic databases: PubMed, EMBASE, Cochrane Central Register of Controlled trials, the Clinical Trials.gov website, China National Knowledge Infrastructure (CNKI), China Science and Technology Journal Database (VIP), Wanfang Database and China Biology Medicine (CBM) from December 2019 to November 2020. Two reviewers independently searched, selected studies, and extracted data according to the eligibility criteria. Cochrane Risk of Bias (ROB) tool was used to assess the methodological quality of the included RCTs. Revman 5.3.0 software was used for statistical analysis.

Results

Twelve eligible RCTs were included with a total sample size of 1393. Our meta-analyses found that lung CT [RR=1.26, 95%CI (1.15, 1.38), $P<0.00001$], and clinical cure rate [RR=1.26, 95%CI (1.16, 1.38), $P<0.00001$] of CHM combined with conventional therapy in the treatment of mild to moderate COVID-19 was better than that of conventional therapy. The rate of conversion to severe cases [RR=0.48, 95%CI (0.32, 0.73), P=0.0005], TCM symptom score of fever [MD=-0.62, 95%CI (-0.79, -0.45), $P<0.00001$], cough cases [RR=1.43, 95%CI (1.16, 1.75), P=0.0006], TCM symptom score of cough[MD=-1.07, 95%CI (-1.29, -0.85), $P<0.00001$], TCM symptom score of fatigue[MD=-0.66, 95%CI (-1.05, -0.28), P=0.0007], and CRP[MD=-5.46, 95%CI (-8.19, -2.72), $P<0.0001$] of CHM combined with conventional therapy was significantly lower than that of conventional therapy. The WBC count was significantly higher than that of conventional therapy[MD=0.38, 95%CI (0.31, 0.44), $P<0.00001$]. Our meta-analysis results were robust and reliable through sensitivity analysis.

Conclusion

Chinese herbal medicine combined with conventional therapy could be effective and safe in the treatment of adults with mild to moderate COVID-19. More high-quality RCTs are needed in the future.

Introduction

Coronavirus disease 2019 (COVID-19) is caused by severe acute respiratory syndrome coronavirus 2 (SARS-CoV-2) [1]. It has the main symptoms of fever, cough, and fatigue [2]. COVID-19 has emerged as a global pandemic since its outbreak in Wuhan, China, in December 2019 [1]. As of March 25, 2021, more than 124.21 million confirmed cases and more than 2.73 million deaths had been reported globally [3]. COVID-19 has developed into a global public health emergency. Therefore, it is an urgent task to control COVID-19 effectively.

In China's experience fighting COVID-19, traditional Chinese medicine (TCM), especially Chinese herbal medicine (CHM), has played an important role [4]. CHM is a special medicine used in the prevention and treatment of diseases in TCM, which is composed of plant medicine, animal medicine, and mineral medicine [5]. A large number of epidemiological investigations showed that mild to moderate COVID-19 accounted for the largest proportion of cases [6]. The current conventional therapy recommendations for mild to moderate COVID-19 are mainly antiviral and symptomatic support treatment [7]. The recommended antiviral drugs are interferon, ribavirin, lopinavir-ritonavir, and chloroquine phosphate, which have been proved beneficial for COVID-19 [7-8]. Many trials have shown that, compared with conventional therapy, CHM has better effects for COVID-19 [9-10].

In our review, randomized controlled trials (RCTs) on CHM in the treatment of adult patients with mild to moderate COVID-19 were searched. The efficacy and safety of CHM in adults with mild to moderate COVID-19 were objectively evaluated by systematic evaluation and meta-analysis.

Methods

This review was conducted based on the Preferred Reporting Items for Systematic Reviews and Meta-Analyses (PRISMA) [11]. The protocol for our review has been registered on the International Prospective Register of Systematic Reviews (PROSPERO) with the registration number CRD42020213528.

Eligibility criteria

Inclusion and exclusion criteria. The diagnostic criteria of mild to moderate COVID-19 refer to " Diagnosis and Treatment Guideline for COVID-19 (Trial 8th Edition) " [7]. Mild COVID-19 is defined as mild clinical symptoms (such as low fever, mild fatigue, impairment of smell and taste, etc.) with no radiographic evidence of pneumonia [7]. Moderate COVID-19 is defined as having fever, respiratory symptoms, and imaging manifestations of pneumonia [7].

Inclusion criteria: (1) Types of studies: randomized controlled trials (RCTs). (2) Types of participants: adult patients (aged≥18 years) diagnosed as mild to moderate COVID-19. (3) Types of interventions: the treatment group was treated with a combination of CHM and conventional therapy. The administration of CHM was limited to oral administration. Patients in the control group were treated with conventional therapy. (4) Types of outcome measures: a. clinical efficacy (e.g. lung computed tomography (CT), clinical cure rate, rate of conversion to severe cases, viral nucleic acid testing), b. clinical symptoms (e.g. fever, cough, fatigue), c. inflammatory biomarkers (e.g. white blood cell (WBC) count, lymphocyte (LYM) count, LYM percentage, neutrophils (NEU) percentage, C-reactive protein (CRP)), d. adverse drug events (e.g. nausea and vomit, diarrhea, liver damage).

Exclusion criteria: (1) Patients with suspected diagnosis of COVID-19; (2) Retrospective studies, observational studies, repeated data studies, and cross-over studies.

Search strategy

RCTs assessing the efficacy and adverse events of CHM for adults with mild to moderate COVID-19 were searched in the following eight electronic databases: PubMed, EMBASE, Cochrane Central Register of Controlled Trials, the Clinical Trials.gov website, China National Knowledge Infrastructure (CNKI), China Science and Technology Journal Database (VIP), Wanfang Database and China Biology Medicine (CBM) from December 2019 to March 2021. There was no language restriction in our review. The search terms included “coronavirus disease 2019”, “COVID-19”, “novel coronavirus pneumonia”, “SARS-CoV-2”, “2019-nCoV”, “traditional Chinese medicine”, “Chinese herbal medicine”, “Chinese herb”, “Chinese herb therapy”, “Chinese herbal formulas”, “clinical trial”, “randomized controlled trial”, “randomised controlled trial”, and “lin chuang yan jiu”. Potential eligible trials were obtained by manually searching the reference list of previously published reviews. If possible, the conference abstracts were reviewed to find unpublished trials, and the data was obtained by contacting the author.

The PubMed search strategy is as follows. Search: ((((((coronavirus disease 2019) OR (COVID-19)) OR (novel coronavirus pneumonia)) OR (SARS-CoV-2)) OR (2019-nCoV)) AND (((((traditional Chinese medicine) OR (Chinese herbal medicine)) OR (Chinese herb)) OR (Chinese herb therapy)) OR (Chinese herbal formulas))) AND ((((clinical trial) OR (randomized controlled trial)) OR (randomised controlled trial)) OR (lin chuang yan jiu))

Study selection and data extraction

In the process of study selection, one reviewer (XQD) independently screened the literature from eight databases according to the eligibility criteria. Duplicate publications were removed. Through reading the title, abstract and full text, the reviewer (XQD) excluded non randomized controlled trials (non-RCTs) and irrelevant trials. The data were extracted independently by two reviewers (XQD and LPS) using

a pre-designed test form in duplicate. The following information was extracted from the included RCTs: basic characteristics (e.g. the title, first authors' name, publication date), participant characteristics (e.g. age, gender, sample size), intervention details (e.g. description of interventions, description of controls, dose, route of oral administration, duration of treatment), and outcome measures, as well as any adverse events. Reviewers (XQD and LPS) cross-checked the data. Any differences of opinion among the primary reviewers were resolved by a third reviewer (WFC). All reviewers were unbiased and had no conflicting interests.

Assessment of methodological quality

The methodological quality of the included RCTs was independently assessed by two reviewers (XQD and LPS) using the Cochrane Collaboration's tool [12]. Seven items of risk of bias (ROB) including adequate sequence generation, concealment of allocation, blinding (patient, investigator and assessor), incomplete outcome data addressed, free of selective reporting, and other biases were evaluated. Each item of ROB was assessed to be low ROB, high ROB, or unclear ROB. Additionally, any disagreements of ROB were resolved by consultation with the third reviewer (WFC).

Meta-analyses

Revman 5.3.0 software (The Cochrane Collaboration, Copenhagen, Denmark) was used for quantitative analysis. The relative risk (RR) was adopted for dichotomous variables. Mean difference (MD) or standard mean difference (SMD) were adopted for continuous variables. Confidence intervals (CIs) were set as 95% with $P < 0.05$ considered as statistically significant difference. Heterogeneity was assessed with the $\chi 2$ test and the I^2 statistical value. When the $P \geq 0.10$ or $I^2 \leq 50\%$, a fixed-effect model was adopted. Otherwise, a random-effect model was applied. We conducted a subgroup analysis of lung CT after 7 days of treatment duration. Sensitivity analysis was performed by leave-one-out method [13]. Funnel plot analysis was performed to evaluate the reporting bias for outcome measures with more than 10 RCTs [14].

Results

Eligible studies

The flow diagram of study selection and identification is showed in (Fig 1). The characteristics of included RCTs are listed in (Table 1). In this review, a total of twelve eligible RCTs were included [15-26]. Among the twelve RCTs [15-26], three were multi-centered trials [18,19,22] and the remaining nine were single-centered trials. All twelve RCTs were conducted in mainland China in 2020. One RCT was published online in English [19], and the rest were reported online in Chinese. The sample size of the included RCTs ranged from 45 to 295 (total 1393). The treatment duration varied from 5 to 15 days. Seven RCTs [16,19-24] described the lung CT. Five RCTs [16-17,19,22,26] described the clinical cure rate. Nine RCTs [16-23,25] described the rate of conversion to severe cases. Four RCTs [18-19,22,25] described the viral nucleic acid testing. Clinical symptoms of fever, cough and fatigue were described in seven RCTs [15-17,21-23,25], of which three RCTs [15,21,25] described fever/cough/fatigue reduction cases, and four RCTs [16-17,23-24] described TCM symptom score of fever/cough/fatigue. Inflammatory biomarkers were described in six RCTs [16-17,22-24,26], of which four RCTs [16-17,23-24] described WBC count, four RCTs [16-17,22-23] described LYM count, three RCTs [16-17,24] described LYM percentage, two RCTs [16,22] described NEU percentage, and six RCTs [16-17,22-24,26] described CRP. Adverse drug events were described in ten RCTs [15-19,22-26].

Table 1. The characteristics of included RCTs.

First author	Type of COVID-19	Sample size (M/F)	Age (yrs)	Intervention
Duan C[15]	mild	T: 82 (39/ 43) C: 41(23/18)	T: 51.99±13.88 C: 50.29±13.17	Jinhua Qinggan granule therapy

Fu[16]	mild/moderat e	T: 32 (17/ 15) C: 33(19/14)	T: 43.26±7.15 C:43.68± 6.45	Toujie Quwen granule + therapy
Fu XX[17]	moderate	T: 37 (19/18) C: 36(19/17)	T: 45.26 ± 7.25 C:44.68 ± 7.45	Toujie Quwen granule + therapy
Hu F[18]	moderate	T: 100 (49/ 51) C: 100(55/45)	T: 47.00±14.06 C: 49.28±11.14	Jinyinhua oral liquid + c
Hu K[19]	mild/moderat e	T: 142 ((79/63) C: 142(71/71)	T:50.4 ± 15.2 C:51.8 ± 14.8	Lianhua Qingwen capsu therapy
Qiu M[20]	moderate	T: 25 (13/ 12) C: 25(14/11)	T: 53.35±18.35 C:51.32 ±14.62	Maxing Xuanfei Jiedu D conventional therapy
Sun HM[21]	mild/moderat e	T: 32 (17/ 15) C: 25(11/14)	T: 45.4±14.10 C:42.0± 11.70	Lianhua Qingke granule therapy
Yang MB[22]	moderate	T: 26(16/ 10) C: 23(9/14)	T: 50.35±13.37 C:47.17 ±16.57	Reyanning mixture + co
Yu P[23]	mild/moderat e	T: 147 (82/65) C: 148(89/59)	T: 48.27±9.56 C:47.25± 8.67	Lianhua Qingwen granu therapy
Zhang CT[24]	moderate	T: 22 (9/ 13) C: 23 (10/13)	T: 53.7 ± 3.5 C: 55.6 ± 4.2	Jiawei Dayuan Decoctio therapy
Zhang YL[25]	moderate	T: 80 (50/ 30) C: 40(23/17)	T: 53.4±13.70 C:52.0± 14.10	Jinyinhua oral liquid + c
Zhou WM[26]	moderate	T: 52 (32/ 20) C: 52(28/24)	T: 52.47±10.99 C:51.11 ±9.87	diammonium glycyrrhizi therapy

Assessment of methodological quality

The methodological quality of the included RCTs was assessed according to the Cochrane Collaboration's tool [12]. As shown in (Fig 2a) and (Fig 2b), green and "+" indicate "Low risk"; yellow and "?" indicate "Unclear". Detailed information on sequence generation of randomization was described in ten trials (10/12, 83.33%) [15-23,26]. Detailed information on allocation concealment was unclear. One RCT reported blinding of the assessor [19]. Detailed information on blinding of patient, investigator, and assessor was not described in the rest eleven RCTs. Attrition bias was scored as 100% low risk. Detailed information on selective reporting was unclear.

Description of CHM

The components of CHM are listed in (Table 2). Nine oral CHM were used in this review, including Jinhua Qinggan granule [15], Toujie Quwen granule [16-17], Jinyinhua oral liquid [18,25], Lianhua Qingwen capsule (granule) [19,23], Maxing Xuanfei Jiedu Decoction [20], Lianhua Qingke granule [21], Reyanning mixture [22], Jiawei Dayuan Decoction [24], diammonium glycyrrhizinate [26]. Among the nine oral CHM, the most frequently used Chinese medicine was honeysuckle, which was used in seven trials (58.33%) [15-19,23,25], followed by forsythia (50.00%) [15-17,19,21,23], and ephedra (50.00%) [15,19-21,23-24].

Four dosage formulations of oral CHM were included in this review, including granule [15-17,21,23-24], oral liquid [18,22,25], capsule [19,26], and decoction [20]. Among the four dosage formulations of oral CHM, the most frequently used Chinese medicine was granule, which was used in six trials (50.00%) [15-17,21,23-24].

Table 2. The components of CHM.

References	CHM	Components

Duan C[15]	Jinhua Qinggan granule	Jinyinhua 10g, Shigao 10g, Mahuang(processed with honey) 10g, Kuxingren(stir-frying) 10g, Huangqin 10g, Lianqiao 10g, Zhebeimu 10g, Zhimu 10g, Niubangzi 10g, Qinghao 10g, Bohe 10g, Gancao10g
Fu[16]	Toujie Quwen granule	Lianqiao 30 g，Shancigu 20 g，Jinyinhua 15 g，Huangqin 10 g， Daqingye 10 g ,Chaihu 5 g ,Qinghao 10 g ,Chantui 10 g，Qianhu 5 g，Chuanbeimu 10 g，Zhebeimu 10 g，Wumei 30 g ,Xuanshen 10 g， Huangqi 45 g ,Fuling 30 g，Taizishen 15 g
Fu XX[17]	Toujie Quwen granule	Lianqiao 30 g，Shancigu 20 g，Jinyinhua 15 g，Huangqin 10 g， Daqingye 10 g ,Chaihu 5 g ,Qinghao 10 g ,Chantui 10 g，Qianhu 5 g，Chuanbeimu 10 g，Zhebeimu 10 g，Wumei 30 g ,Xuanshen 10 g， Huangqi 45 g ,Fuling 30 g，Taizishen 15 g
Hu F[18]	Jinyinhua oral liquid	Jinyinhua 5.4g
Hu K[19]	Lianhua Qingwen capsule	Lianqiao, Jinyinhua, Mahuang(stir-frying), Kuxingren(stir-frying), Shigao, Banlangen, Guanzhong, Yuxingcao, Huoxiang, Dahuang, Hongjingtian, Bohe, Gancao
Qiu M[20]	Maxing Xuanfei	Mahuang 9 g ,Kuxingren 12 g ,Shigao 15~30 g ,Zhebeimu 12 g， Chantui 10 g，Jiangchan 15 g，Jianghuang 12 g，

	Jiedu Decoction	Jiegeng 12 g，Zhiqiao 12 g，Caoguo 9 g，Caodoukou 12 g
Sun HM[21]	Lianhua Qingke granule	Mahuang, Sangbaipi, Kuxingren(stir-frying), Lianqiao, mountain honeysuckle, Dahuang
Yang MB[22]	Reyanning mixture	Pugongying, Huzhang, Baijiang Herba cum Radice, Banzhilian
Yu P[23]	Lianhua Qingwen granule	Lianqiao, Jinyinhua, Mahuang(stir-frying), Kuxingren(stir-frying), Shigao, Banlangen, Guanzhong, Yuxingcao, Huoxiang, Dahuang, Hongjingtian, Bohe, Gancao
Zhang CT[24]	Jiawei Dayuan Decoction	Mahuang(stir-frying) 10 g, Xingren 15 g, crude gypsum 20 g, trichosanthes bark 20 g, Dahuang(Stir-fry with yellow rice wine) 6 g, Tinglizi 10g, Taoren 10 g, Caoguo 6 g, Binglang 10 g, Cangzhu 10 g
Zhang YL[25]	Jinyinhua oral liquid	Jinyinhua 5.4g
Zhou WM[26]	diamine glycyrrhizinate	diamine glycyrrhizinate

Efficacy and safety assessment

Clinical efficacy

Clinical efficacy was reported in eleven RCTs [16-26], of which seven RCTs [16,19-24] reported lung CT, five RCTs [16-17,19,22,26] reported clinical cure rate, nine RCTs [16-23,25] reported rate of conversion to severe cases, and four RCTs [18-19,22,25] reported viral nucleic acid testing. Evaluation criteria for lung CT refer to COVID-19 Guidelines for Imaging Assisted Diagnosis [27]. Clinical cure standards refer to Guiding Principles for Clinical Research of New Chinese Materia Medica [28]. The therapeutic effects are classified as effective, improved, and ineffective. Clinical cure rate = (effective cases + improved cases) / total cases × 100%.

In the field of lung CT, 426 patients were in the treatment group and 419 in the control group. In the field of clinical cure rate, 410 patients were in the treatment group and 411 in the control group. In the field of rate of conversion to severe cases, 578 patients were in the treatment group and 543 in the control group. In the field of viral nucleic acid testing, 305 patients were in the treatment group and 276 in the control group.

According to lung CT, meta-analysis and subgroup analysis results are shown in (Fig 3a). In terms of lung CT, Chi^2 test shows that I^2=8% (＜50%), P=0.37. In terms of lung CT after 7 days of treatment duration, Chi^2 test shows that I^2=44% (＜50%), P=0.17. In terms of lung CT of 10 to 14 days treatment duration, Chi^2 test shows that I^2=0% (＜50%), P=0.87. It shows that there is no heterogeneity between the included trials, so we choose to use fixed-effect model for meta-analysis and subgroup analyses. It can be seen from the forest plot that compared with conventional therapy, CHM combined with conventional therapy exhibited a significant improvement on lung CT [7 trials, n=845, RR=1.26, 95%CI (1.15, 1.38), P＜0.00001] (Fig 3a). Subgroup analysis revealed an improvement on lung CT after 7 days of treatment duration by CHM combined with conventional therapy [n=845, RR=1.18, 95%CI (1.02, 1.36), P=0.03] (Fig 3a); a significant improvement on lung CT of 10 to 14 days treatment

duration by CHM combined with conventional therapy [n=845, RR=1.34, 95%CI (1.19, 1.50), $P<0.00001$] (Fig 3a).

According to clinical cure rate and rate of conversion to severe cases, meta-analysis results are shown in (Fig 3b) and (Fig 3c). In terms of clinical cure rate, Chi^2 test shows that $I^2=0\%$ ($<50\%$), $P=0.77$. In terms of rate of conversion to severe cases, Chi^2 test shows that $I^2=0\%$ ($<50\%$), $P=0.83$. It shows that there is no heterogeneity between the included trials, so we choose to use fixed-effect model for analyses. It can be seen from the forest plot that compared with conventional therapy, CHM combined with conventional therapy exhibited a significant improvement on clinical cure rate [5 trials, n=821, RR=1.26, 95%CI (1.16, 1.38), $P<0.00001$] (Fig 3b); a significant reduction in rate of conversion to severe cases [9 trials, n=1121, RR=0.48, 95%CI (0.32, 0.73), $P=0.0005$] (Fig 3c).

According to viral nucleic acid testing, meta-analysis result is shown in (Fig 3d). Chi^2 test shows that $I^2=57\%$ ($>50\%$), $P=0.08$. It shows that there is heterogeneity between the included trials, so we choose to use random-effects model for analysis. It can be seen from the forest plot that no statistical difference in viral nucleic acid testing was identified between the treatment group and the control group [4 trials, n=581, RR=1.09, 95%CI (0.98, 1.21), $P=0.13$] (Fig 3d).

Clinical symptoms

Clinical symptoms of fever, cough and fatigue was reported in seven RCTs [15-17,21-23,25]. Among them, three RCTs [15,21,25] reported number of fever/cough/fatigue reduction cases, and four RCTs [16-17,22-23] reported TCM symptom score of fever/cough/fatigue.

In the field of fever reduction cases, 138 patients were in the treatment group and 67 in the control group. In the field of cough reduction cases, 156 patients were in the treatment group and 77 in the control group. In the field of fatigue reduction cases, 130 patients were in the treatment group and 57 in the control group. In the field of

TCM symptom score of fever/cough/fatigue, there were 242 patients in the treatment group and 240 in the control group.

According to fever reduction cases, cough reduction cases, and fatigue reduction cases, meta-analysis results are shown in (Fig 4a), (Fig 4b), and (Fig 4c). In terms of fever reduction cases, Chi^2 test shows that I^2=95% (>50%), P<0.00001. It shows that there is heterogeneity between the included trials, so we choose to use random-effects model for analysis. It can be seen from the forest plot that no statistical difference in number of fever reduction cases was identified between the treatment group and the control group [3 trials, n=205, RR=1.14, 95%CI (0.58, 2.25), P=0.70] (Fig 4a). In terms of cough reduction cases, Chi^2 test shows that I^2=0% (< 50%), P=0.89. It shows that there is no heterogeneity between the included trials, so we choose to use fixed-effect model for analysis. It can be seen from the forest plot that compared with conventional therapy, CHM combined with conventional therapy revealed a significant reduction in cough cases [3 trials, n=205, RR=1.43, 95%CI (1.16, 1.75), P=0.0006] (Fig 4b). In terms of fatigue reduction cases, Chi^2 test shows that I^2=28% (<50%), P=0.25. It shows that there is no heterogeneity between the included trials, so we choose to use fixed-effect model for analysis. It can be seen from the forest plot that CHM combined with conventional therapy revealed a significant reduction in fatigue cases [3 trials, n=205, RR=1.23, 95%CI (1.03, 1.47), I^2=28%, P=0.02] (Fig 4c).

According to TCM symptom score of fever, cough, and fatigue, meta-analysis results are shown in (Fig 4d), (Fig 4e), and (Fig 4f). In terms of TCM symptom score of fever, Chi^2 test shows that I^2=79% (>50%), P=0.009. In terms of TCM symptom score of cough, Chi^2 test shows that I^2=84% (>50%), P=0.0003. In terms of TCM symptom score of fatigue, Chi^2 test shows that I^2=98% (>50%), P<0.00001. It shows that there is heterogeneity between the included trials, so we choose to use random-effects model for analyses. It can be seen from the forest plot that compared with conventional therapy, CHM combined with conventional therapy revealed a significant reduction in TCM symptom score of fever [4 trials, n=482, MD=-0.62,

95%CI (-0.79, -0.45), $P < 0.00001$] (Fig 4d); a significant reduction in TCM symptom score of cough [4 trials, n=482, MD=-1.07, 95%CI (-1.29, -0.85), $P < 0.00001$] (Fig 4e); a significant reduction in TCM symptom score of fatigue [4 trials, n=482, MD=-0.66, 95%CI (-1.05, -0.28), P=0.0007] (Fig 4f).

Inflammatory biomarkers

Inflammatory biomarkers were reported in six RCTs [16-17,22-24,26], of which four RCTs [16-17,23-24] reported WBC count, three RCTs [16-17,24] reported LYM percentage, four RCTs [16-17,22-23] reported LYM count, two RCTs [16,22] reported NEU percentage, and six RCTs [16-17,22-24,26] reported CRP.

In the field of WBC count, 238 patients were in the treatment group and 240 in the control group. In the field of LYM count, 242 patients were in the treatment group and 240 in the control group. In the field of LYM percentage, 91 patients were in the treatment group and 92 in the control group. In the field of NEU percentage, 58 patients were in the treatment group and 56 in the control group. In the field of CRP, 316 patients were in the treatment group and 315 in the control group.

According to WBC count and NEU percentage, meta-analysis results are shown in (Fig 5a) and (Fig 5b). In terms of WBC count, Chi^2 test shows that I^2=5% (<50%), P=0.37. In terms of NEU percentage, Chi^2 test shows that I^2=0% (<50%), P=0.88. It shows that there is no heterogeneity between the included trials, so we choose to use fixed-effect model for analyses. It can be seen from the forest plot that compared with conventional therapy, CHM combined with conventional therapy revealed a significant improvement on WBC count [4 trials, n=478, MD=0.38, 95%CI (0.31, 0.44), I^2=5%, $P < 0.00001$] (Fig 5a); a significant reduction in NEU percentage [2 trials, n=114, MD=-4.56, 95%CI (-5.76, -3.36), I^2=0%, $P < 0.00001$] (Fig 5b).

According to LYM count, LYM percentage, and CRP, meta-analysis results are shown in (Fig 5c), (Fig 5d), and (Fig 5e). In terms of LYM count, Chi^2 test shows that I^2=97% (>50%), $P < 0.00001$. In terms of LYM percentage, Chi^2 test shows that I^2=93% (>50%), $P < 0.00001$. In terms of CRP, Chi^2 test shows that I^2=96% (>

50%), $P < 0.00001$. It shows that there is heterogeneity between the included trials, so we choose to use random-effects model for analyses. It can be seen from the forest plot that compared with conventional therapy, CHM combined with conventional therapy revealed a significant improvement on LYM count [4 trials, n=482, MD=0.26, 95%CI (0.05, 0.47), P=0.01] (Fig 5c); a significant improvement on LYM percentage [3 trials, n=183, MD=6.65, 95%CI (3.36, 9.94), $P < 0.0001$] (Fig 5d); a significant reduction in CRP [6 trials, n=631, MD=-5.46, 95%CI (-8.19, -2.72), $P < 0.0001$] (Fig 5e).

Adverse drug events

In this review, adverse drug events were reported in ten RCTs [15-19,22-26], while the remaining two RCTs [20-21] did not. Among ten RCTs [15-19,22-26], no adverse event was identified in either treatment or control groups [16-17,22-24]. Adverse drug events in the remaining five RCTs included gastrointestinal reactions (diarrhea, poor appetite, nausea, vomiting), headache, renal dysfunction, and abnormal liver function [15,18-19,25-26]. All reported adverse drug events were mild in the treatment and control groups, and were tolerable or alleviated after withdrawal.

In the field of total number of adverse drug events cases, 413 patients were in the treatment group and 346 in the control group. In the field of nausea and vomiting, 194 patients were in the treatment group and 194 in the control group. In the field of diarrhea, 413 patients were in the treatment group and 346 in the control group. In the field of abnormal liver function, 194 patients were in the treatment group and 194 in the control group.

According to total number of adverse drug events cases, meta-analysis result is shown in (Fig 6a). In terms of total number of adverse drug events cases, Chi^2 test shows that I^2=63% ($>$50%), P=0.03. It shows that there is heterogeneity between the included trials, so we choose to use random-effects model for analysis. It can be seen from the forest plot that no statistical difference in total number of adverse drug events cases was identified between the treatment group and the control group [5

trials, n=759, RR=1.13, 95%CI (0.45, 2.83), *P*=0.79] (Fig 6a). According to nausea and vomiting, meta-analysis result is shown in (Fig 6b). In terms of nausea and vomiting, Chi^2 test shows that I^2=0% (＜50%), *P*=0.74. It shows that there is no heterogeneity between the included trials, so we choose to use fixed-effect model for analysis. It can be seen from the forest plot that subgroup analysis revealed no statistical difference in nausea and vomiting [2 trials, n=388, RR=1.09, 95%CI (0.49, 2.41), *P*=0.83] (Fig 6b).

According to diarrhea and abnormal liver function, meta-analysis results are shown in (Fig 6c) and (Fig 6d). In terms of diarrhea, Chi^2 test shows that I^2=70% (＞50%), *P*=0.009. In terms of abnormal liver function, Chi^2 test shows that I^2=78% (＞50%), *P*=0.03. It shows that there is heterogeneity between the included trials, so we choose to use random-effects model for analyses. It can be seen from the forest plot that subgroup analysis revealed statistical difference in diarrhea [5 trials, n=759, RR=1.72, 95%CI (0.34, 8.67), *P*=0.51] (Fig 6c); and abnormal liver function [2 trials, n=388, RR=0.41, 95%CI (0.05, 3.69), *P*=0.43] (Fig 6d). Poor appetite, headache, and renal dysfunction were reported in one RCT [19], with no statistical difference identified between the treatment group and the control group.

Sensitivity analysis

Sensitivity analysis revealed that there was a small change in the effect amount, and was a significant difference in lung CT, clinical cure rate, rate of conversion to severe cases, TCM symptom score of fever, number of cough reduction cases, TCM symptom score of cough, TCM symptom score of fatigue, WBC count, and CRP, which indicated the above meta-analysis results to be robust and reliable.

Publication bias

In our study, ten trials [15-19,22-26] reported adverse drug events. Among ten trials [15-19,22-26], five trials [16-17,22-24] reported no adverse event identified in either treatment or control groups. The funnel plot was used to analyze the reported

adverse events trials to explore the bias (Fig 7). The funnel plot is symmetrical, indicating no obvious deviation.

Discussion

The clinical classification of COVID-19 is mild, moderate, severe, and critical [7]. Severe COVID-19 is more likely to have serious complications, such as shock, acute respiratory distress syndrome (ARDS), arrhythmia, and acute heart injury [29-30], all of which significantly increase the difficulty and cost of treatment. Therefore, it is of great significance to prevent COVID-19 from developing from mild or moderate to severe. In our study, it was found that compared with conventional therapy, mild to moderate COVID-19 patients treated with both CHM and conventional therapy had more benefit. Similar studies have shown that CHM has positive effects in COVID-19 patients [31-33]. Facing such a severe COVID-19 epidemic, Western countries should pay attention to the therapeutic effect of CHM for COVID-19.

According to the theory of TCM, epidemic disease refers to an acute infectious disease characterized by sudden onset, rapid transmission, dangerous condition, and strong infectivity after feeling pestilence evil [34]. COVID-19 belongs to the "epidemic disease" of TCM, in the light of its incidence mode and epidemic trend [7]. The pathogenesis of mild to moderate COVID-19 is dampness-heat or cold-dampness obstructing the lung [7]. Therefore, CHM with the effect of heat-clearing, eliminating dampness, resolving phlegm, and dispersing cold is widely used [7]. In the included studies, nine different oral CHM were used, including Lianhua Qingwen capsule (granule), Toujie Quwen granule, Jinyinhua oral liquid, diammonium glycyrrhizinate, etc. Lianhua Qingwen capsule is originated from classical Chinese herbal formulas, which can decrease influenza A virus (H1N1) replication, lung lesions, and inflammation [35]. Also, Lianhua Qingwen capsule may reduce lung injury and help eliminate SARS- CoV- 2 infection by regulating Akt1 [36]. One study has shown that Toujie Quwen granule may have therapeutic effects on COVID-19 by regulating

SARS- CoV- 2 infection, immune and inflammation-related targets, and pathways [37]. Diammonium glycyrrhizinate is used as a hepatic protector, which is the main component of licorice root extracts [38]. Diammonium glycyrrhizinate can decrease the serum ALT and AST levels and improve the histological damage, down-regulated the inflammatory cytokines, inhibited the apoptosis of T lymphocytes in the thymus [38].

Among the nine oral CHM, the most frequently used Chinese medicine was honeysuckle, followed by forsythia, and ephedra. Honeysuckle and forsythia have the function of clearing heat-toxicity and dispersing wind-heat in the theory of TCM [5]. Honeysuckle polysaccharide is an active component of honeysuckle, which can regulate non-specific immunity [39], inhibit the expression of inflammatory factors TNF-α and IL-1β [40], and inhibit a variety of viruses [41]. Phillyrin is an active component of forsythia, which has antiviral and anti-inflammatory activities [42-43]. Ephedra has the function of dissipating cold and diffusing the lung to calm panting in TCM theory [5]. Ephedrine is an active component of ephedra, which can increase the production of anti-inflammatory cytokines IL-10, reduce the production of pro-inflammatory cytokines TNF-α and IL-12 [44], and play an antiviral role by inhibiting viral replication [45].

Mild to moderate COVID-19 patients treated with both CHM and conventional therapy had better outcomes in the parameters including clinical efficacy, clinical symptoms, and inflammatory response. Our study found that compared with conventional therapy, CHM combined with conventional therapy can improve the scores of symptoms such as fever, cough, and fatigue, and reduce cough cases. This is related to CHM can affect the production of inflammatory cytokines [35,38]. Cytokine storm is perhaps one of the critical hallmarks of COVID-19 severity [46]. Cytokine storm is a hyperproduction of proinflammatory cytokines, which leads to ARDS aggravation and widespread tissue damage resulting in multi-organ failure [46-47]. In our study, we found that CHM combined with conventional therapy can increase WBC count, and reduce CRP. CHM combined with conventional therapy

had a better effect on improving lung CT, promoting clinical cure rate, and reducing rate of conversion to severe cases.

Due to different formulations and unclear composition, CHM has many unknown factors to be solved. In our study, we found that CHM formulations used in the treatment group are different, and quality of herbal intervention is unclear. CHM is likely to require a standard treatment. Besides, quality of herbal formula should be monitored through standardized. In this way, the best evidence can be systematically summarized to better provide an evidence-based basis for TCM decision-making. CHM treatment, which is based on individualized assessment, can be affected by different diet practices, and weather, resulting in its difficulty of using in western countries. Therefore, we think it is necessary for Western countries to hire TCM experts to participate in the treatment of COVID-19. Safety issues should be a concern when CHM is used for COVID-19. In our study, we found that most of the included trials reported adverse drug events. CHM combined with conventional therapy did not increase adverse drug events. The funnel plot of adverse drug events indicated no obvious deviation.

However, it was a common problem that most of the included trials had poor methodological design and that the merger statistical analysis of some outcomes had unexplained heterogeneity. More high-quality trials are needed in the future. Despite the poor methodology and the unexplained heterogeneity, our finding is very valuable and timely in view of no specific drugs approved for COVID-19.

Limitations

Despite the usefulness of our finding, this review also has several limitations that could be improved upon in future studies. First of all, most of the included trials had deficiencies in methodology design, including hidden allocation and inadequate reporting of blind methods. Secondly, the composition, dosage, and frequency of CHM were different in the treatment groups. Thirdly, the multicenter trials were

lacking. In addition, the duration of the included trials ranged from 5 to 15 days. Therefore, it is necessary to design more high-quality trials with a multicenter, large sample, and longer follow-up to better observe the efficacy and possible adverse events of CHM combined with conventional therapy in the treatment of adults with mild to moderate COVID-19.

Conclusion

Chinese herbal medicine combined with conventional therapy could be effective and safe in the treatment of adults with mild to moderate COVID-19. It can improve the clinical cure rate, main clinical symptoms, imaging and laboratory indexes, and reduce the rate of conversion to severe cases. However, due to the fact that COVID-19 is a sudden disease, it is difficult to carry out double-blind clinical trials. This leads to insufficient methodology of the existing-related trials. Therefore, more high-quality trials are needed to evaluate the efficacy and safety of Chinese herbal medicine combined with conventional therapy in the treatment of adults with mild to moderate COVID-19 in the future.

Author Contributions

Conceptualization: Lipeng Shi, Wenfu Cao.

Data curation: Xuqin Du, Lipeng Shi.

Formal analysis: Xuqin Du.

Funding acquisition: Xuqin Du.

Investigation: Xuqin Du, Lipeng Shi.

Methodology: Xuqin Du, Lipeng Shi.

Software: Xuqin Du.

Supervision: Wenfu Cao.

Validation: Biao Zuo, Aimin Zhou.

Writing – original draft: Xuqin Du.

Writing – review & editing: Xuqin Du, Lipeng Shi, Wenfu Cao, Biao Zuo, Aimin Zhou.

References

1. Sattler A, Angermair S, Stockmann H, et al. SARS-CoV-2-specific T cell responses and correlations with COVID-19 patient predisposition[J]. J Clin Invest, 2020,130(12):6477-6489.
2. Chen N, Zhou M, Dong X, et al. Epidemiological and clinical characteristics of 99 cases of 2019 novel coronavirus pneumonia in Wuhan, China: a descriptive study [J]. Lancet, 2020,395(10223):507-513.
3. World Health Organization. WHO Coronavirus (COVID-19) Dashboard.2021 [EB/OL]. https://covid19.who.int/(accessed Mar 26, 2021)
4. Ren JL, Zhang AH, Wang XJ. Traditional Chinese medicine for COVID-19 treatment[J]. Pharmacol Res, 2020, 155:104743.
5. Gao XM. Traditional Chinese Pharmacy (advanced series of traditional Chinese medicine) [M]. Beijing: People's Health Publishing House, 2000.
6. Wu ZY, McGoogan JM. Characteristics of and important lessons from the coronavirus disease 2019(COVID-19) outbreak in China[J]. J Am Med Assoc, 2020, 323(13): 1239-1242.
7. Diagnosis and Treatment Guideline for COVID-19 (Trial 8th Edition) [J]. Chin J Viral Dis, 2020,10(05):321-328.
8. Hung IF, Lung KC, Tso EY, et al. Triple combination of interferon beta-1b, lopinavir-ritonavir, and ribavirin in the treatment of patients admitted to hospital with COVID-19: an open-label, randomised, phase 2 trial[J]. Lancet, 2020,395(10238):1695-1704.
9. Shi N, Guo L, Liu B, et al. Efficacy and safety of Chinese herbal medicine versus Lopinavir-Ritonavir in adult patients with coronavirus disease 2019: A

non-randomized controlled trial[J]. Phytomedicine, 2021, 81:153367.

10. Xiao M, Tian J, Zhou Y, et al. Efficacy of Huoxiang Zhengqi dropping pills and Lianhua Qingwen granules in treatment of COVID-19: A randomized controlled trial[J]. Pharmacol Res, 2020, 161:105126.
11. Moher D, Liberati A, Tetzlaff J, et al. Preferred reporting items for systematic reviews and meta-analyses: the PRISMA statement[J]. Ann Intern Med, 2009,151(4):264-W64.
12. Higgins JPT, Green S. Cochrane Reviewers' Handbook 5.3.0 [updated March 2014], Review Manager (RevMan) [Computer program]. Version 5.3.0. Available from www.cochrane-handbook.org
13. Panahi Y, Hosseini MS, Khalili N, et al. Antioxidant and anti-inflammatory effects of curcuminoid-piperine combination in subjects with metabolic syndrome: A randomized controlled trial and an updated meta-analysis[J]. Clin Nutr, 2015,34(6):1101-1108.
14. Liu M, Gao Y, Yuan Y, et al. Efficacy and safety of integrated traditional Chinese and western medicine for corona virus disease 2019 (COVID-19): a systematic review and meta-analysis[J]. Pharmacol Res, 2020, 158:104896.
15. Duan C, Xia GW, Zheng CJ, et al. Clinical observation on Jinhua Qinggan granule combined with conventional western medicine therapy in treating mild cases of coronavirus disease 2019 [J]. J Tradit Chin Med,2020,61(17):1473-1477.
16. Fu XX, Lin LP, Tan XX. Clinical observation on effect of Toujie Quwen granules in treatment of COVID-19 [J]. Chin J Exp Tradit Med Formul,2020,26(12):44-48.
17. Fu XX, Lin LP, Tan XX. Clinical study on 37 case of COVID- 19 treated with integrated traditional Chinese and western medicine [J]. Tradit Chin Drug Res Clin Pharmacol,2020,31(05):600-604.
18. Hu F, Guo AH, Huang L, et al. Multi-center clinical observation of Jinyinhua oral liquid combined with western medicine in treatment of moderate COVID-19 [J/OL]. J Tradit Chin Med:1-6[2020-11-4]. http://kns.cnki.net/kcms/detail/11.2166.R.20200819.0837.002.html.
19. Hu K, Guan WJ, Bi Y, et al. Efficacy and safety of Lianhuaqingwen capsules, a

repurposed Chinese herb, in patients with coronavirus disease 2019: A multicenter, prospective, randomized controlled trial [J/OL]. Phytomedicine: 153242[2020-05-16]. https://pubmed.ncbi.nlm.nih.gov/32425361/

20. Qiu M, Li Q T, Zhu DP, et al. Efficacy observation of Maxing Xuanfei Jiedu decoction on moderate COVID-19 patients[J]. J Emerg Tradit Chin Med,2020,29(07):1129-1130+1132.
21. Sun HM, Xu F, Zhang L, et al. Study on clinical efficacy of Lianhua Qingke granule in treatment of mild and ordinary COVID-19 [J]. Chin J Exp Tradit Med Formul,2020,26(14):29-34.
22. Yang MB, Dang SS, Haung S, et al. Multi-center clinical observation of Reyanning mixture in treatment of COVID-19 [J]. Chin J Exp Tradit Med Formul,2020,26(14):7-12.
23. Yu P, Li YZ, Wan SB, et al. Clinical efficacy of Lianhua Qingwen granule combined with abidol in treating coronavirus disease 2019 [J]. Chin Pharm J,2020,55(12):1042-1045.
24. Zhang CT, Yang Y, You FM, et al. Clinical study on COVID-19 from the perspective of "Yidujiashi" theory [J]. Pharmacol Clin Chin Materia Medica,2020,36(02):43-45.
25. Zhang YL, Lei L, Xu Y, et al. Clinical efficacy of Jinyinhua oral liquid in the treatment of 80 patients with coronavirus disease 2019 [J]. Chin Pharm,2020,29(09):23-26.
26. Zhou WM, Zhao FM, Li BL, et al. Clinical value of diammonium glycyrrhizinate in treatment of COVID-19 [J]. Chin J Virol,2020,36(02):160-164.
27. Professional Committee of infection and inflammation radiology of Chinese Research Hospital Association. Guidelines for Imaging Assisted Diagnosis of COVID-19 [J]. Chin J Med Imaging Technol,2020, 36(3):1-11.
28. Ministry of Health of the People's Republic of China. Guiding Principles for Clinical Research of New Chinese Materia Medica [M]. Beijing: China Medical Science and Technology Press, 2002:186.
29. Chen N, Zhou M, Dong X, et al. Epidemiological and clinical characteristics of

99 cases of 2019 novel coronavirus pneumonia in Wuhan, China: a descriptive study [J]. Lancet, 2020,395(10223):507-513.

30. Wang DW, Hu B, Hu C, et al. Clinical characteristics of 138 hospitalized patients with 2019 novel coronavirus-infected pneumonia in Wuhan, China [J]. JAMA, 2020, 323(11): 1061-1069.
31. Xiong X, Wang P, Su K, et al. Chinese herbal medicine for coronavirus disease 2019: A systematic review and meta-analysis[J]. Pharmacol Res, 2020, 160:105056.
32. Zeng M, Li L, Wu Z. Traditional Chinese medicine Lianhua Qingwen treating corona virus disease 2019(COVID-19): Meta-analysis of randomized controlled trials[J]. PLoS One, 2020, 15(9): e0238828.
33. Ang L, Song E, Lee HW, et al. Herbal medicine for the treatment of coronavirus disease 2019 (COVID-19): A systematic review and meta-analysis of randomized controlled trials[J]. J Clin Med, 2020,9(5):1583.
34. Yang J. Warm Diseases [M]. Beijing: People's Health Publishing House, 2006.
35. Gao D, Niu M, Wei SZ, et al. Identification of a pharmacological biomarker for the bioassay-based quality control of a thirteen-component TCM formula (Lianhua Qingwen) used in treating influenza A virus (H1N1) infection[J]. Front Pharmacol, 2020,11:746.
36. Xia QD, Xun Y, Lu JL, et al. Network pharmacology and molecular docking analyses on Lianhua Qingwen capsule indicate Akt1 is a potential target to treat and prevent COVID-19[J]. Cell Prolif, 2020,53(12):e12949.
37. Ye M, Luo G, Ye D, et al. Network pharmacology, molecular docking integrated surface plasmon resonance technology reveals the mechanism of Toujie Quwen Granules against coronavirus disease 2019 pneumonia [J/OL]. Phytomedicine: 153401 [2020-10-28]. https://pubmed.ncbi.nlm.nih.gov/33191068/
38. Gao M, Li X, He L, et al. Diammonium glycyrrhizinate mitigates liver injury via inhibiting proliferation of NKT cells and promoting proliferation of tregs[J]. Drug Des Devel Ther,2019,13:3579-3589.
39. Zhou XN, Dong Q, Kan XZ, et al. Immunomodulatory activity of a novel

polysaccharide from Lonicera japonica immunosuppressed mice induced by cyclophosphamide [J]. PloS one,2018,13(10):1-15.

40. Bai XY, Chai YW, Shi WL, et al. Lonicera japonica poly-saccharides attenuate ovalbumin-induced allergic rhinitis by regulation of Th17 cells in BALB/c mice[J]. J Functional Foods,2020,65:103758.
41. Wang J, Hou L, Chen YQ, et al. Extraction and purification of the Lonicera japonica polysaccharide and its antiviral activity in vitro[J]. Chin J Hosp Pharm, 2018,38(8): 810-812.
42. Qu XY, Li QJ, Zhang HM, et al. Protective effects of phillyrin against influenza a virus in vivo[J]. Arch Pharm Res, 2016, 39(7):998-1005.29.
43. Jiang Q, Chen J, Long X, et al. Phillyrin protects mice from traumatic brain injury by inhibiting the inflammation of microglia via PPARγ signaling pathway[J]. Int Immunopharmacol, 2020,79:106083.
44. He W, Ma J, Chen Y, et al. Ephedrine hydrochloride protects mice from staphylococcus aureus-induced peritonitis[J]. Am J Transl Res, 2018,10(3):670-683.
45. Wei W, Du H, Shao C, et al. Screening of antiviral components of Ma Huang Tang and investigation on the ephedra alkaloids efficacy on influenza virus type A[J]. Front Pharmacol, 2019,10:961.
46. Fara A, Mitrev Z, Rosalia RA, et al. Cytokine storm and COVID-19: a chronicle of pro-inflammatory cytokines[J]. Open Biol,2020,10(9):200160.
47. Ragab D, Salah Eldin H, Taeimah M, et al. The COVID-19 cytokine storm; what we know so far[J]. Front Immunol, 2020,11:1446.

Supporting information

S1 Checklist. PRISMA 2009 checklist.

Figure Click here to access/download;Figure;Fig 1.tif

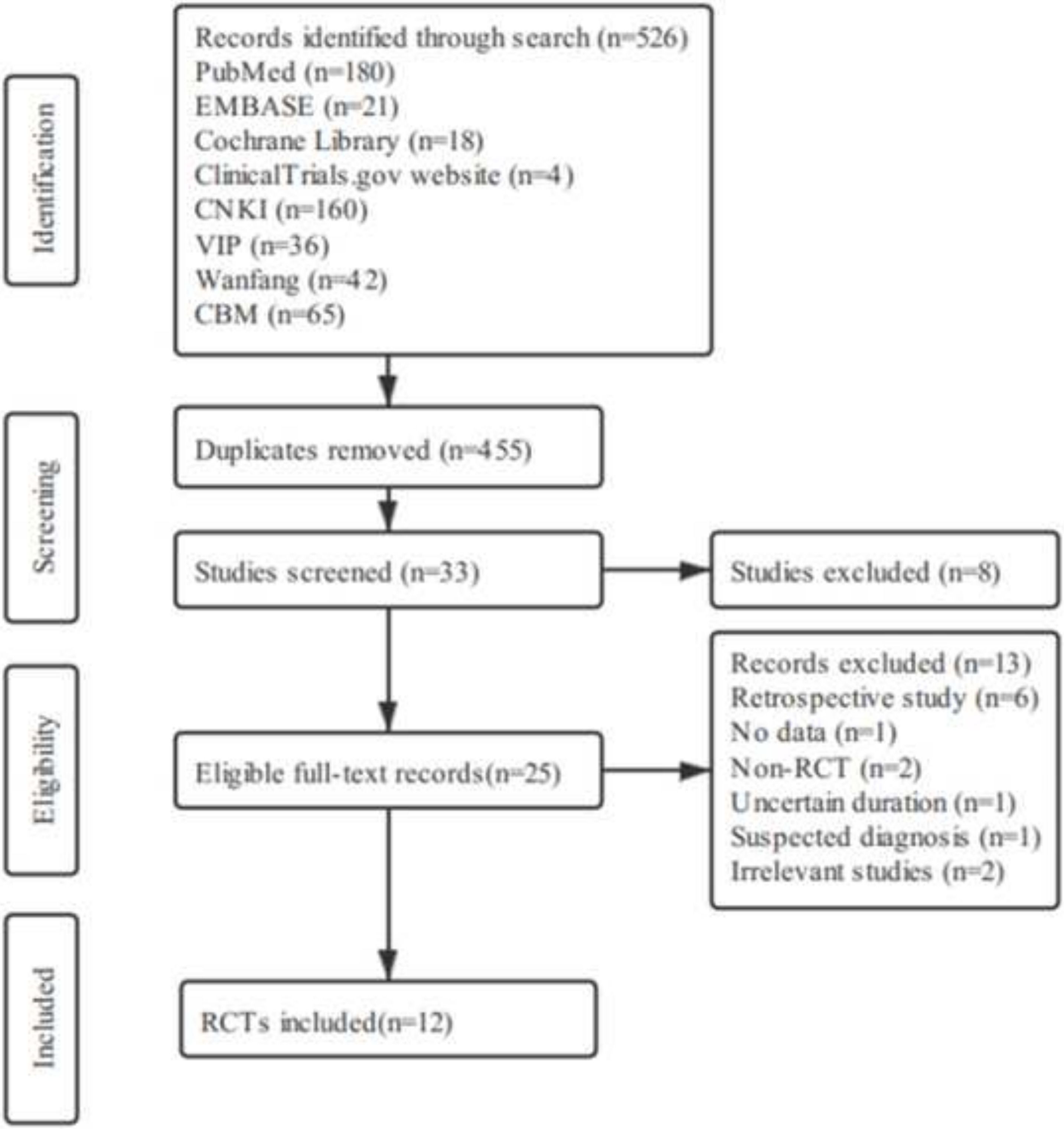

Fig 1. The flow diagram of study selection and identification.

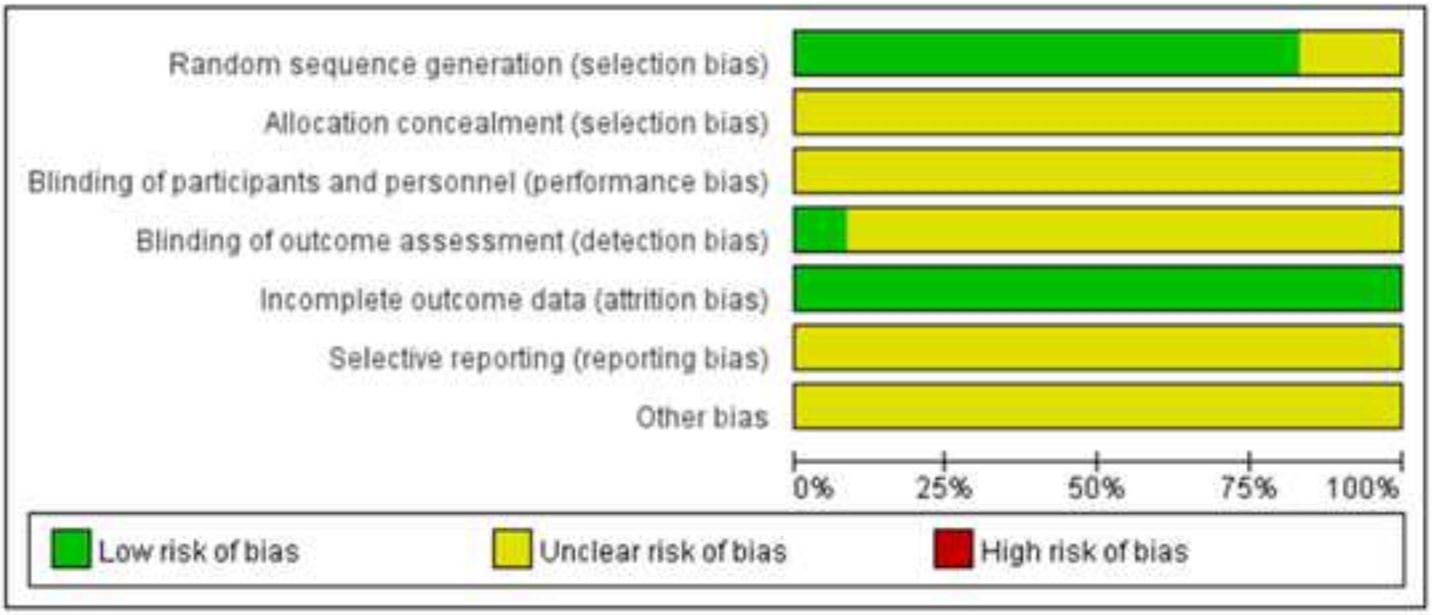

Fig 2a. Risk of bias graph.

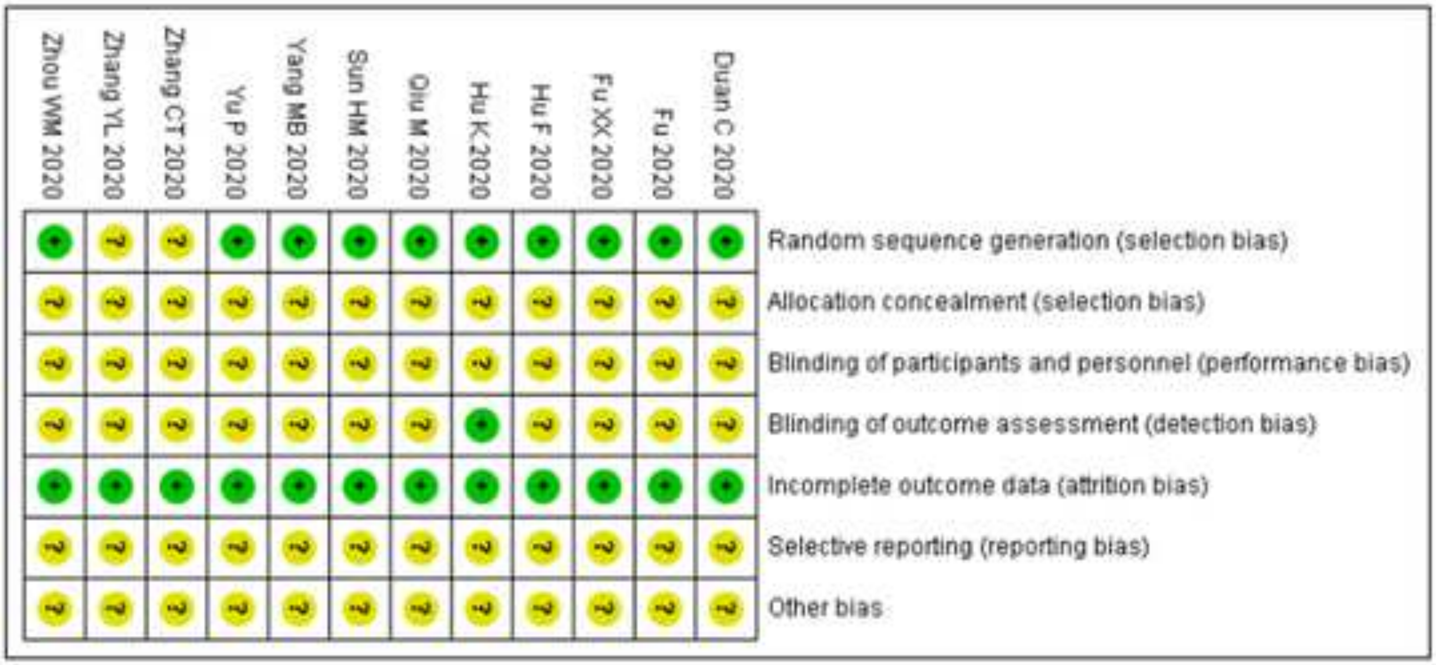

Fig 2b. Risk of bias summary.

Fig 2. Assessment of methodological quality. Fig 2a. Risk of bias graph. Fig 2b. Risk of bias summary.

Figure

Click here to access/download;Figure;Fig 3.tif

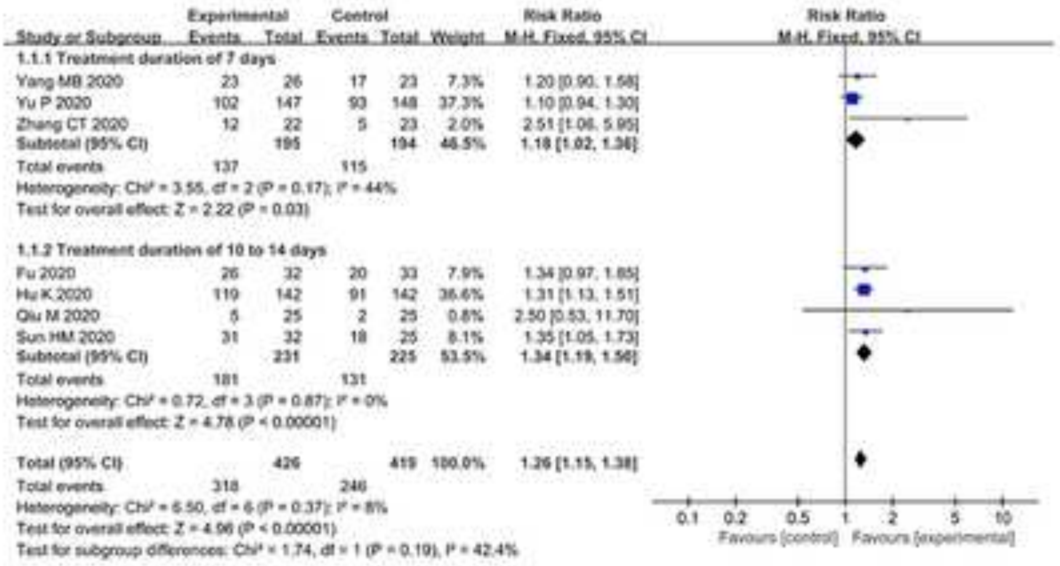

Fig 3a. Lung CT.

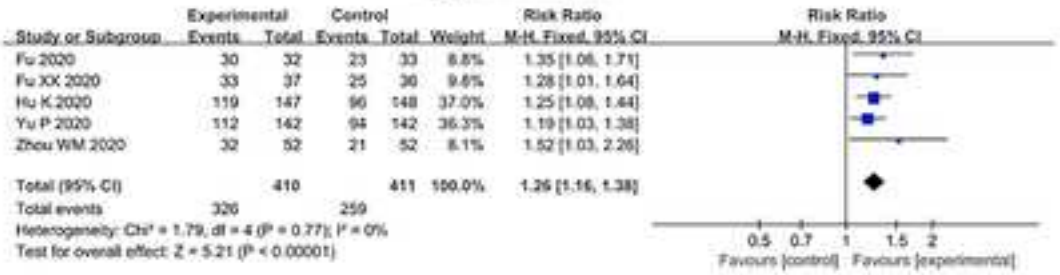

Fig 3b. Clinical cure rate.

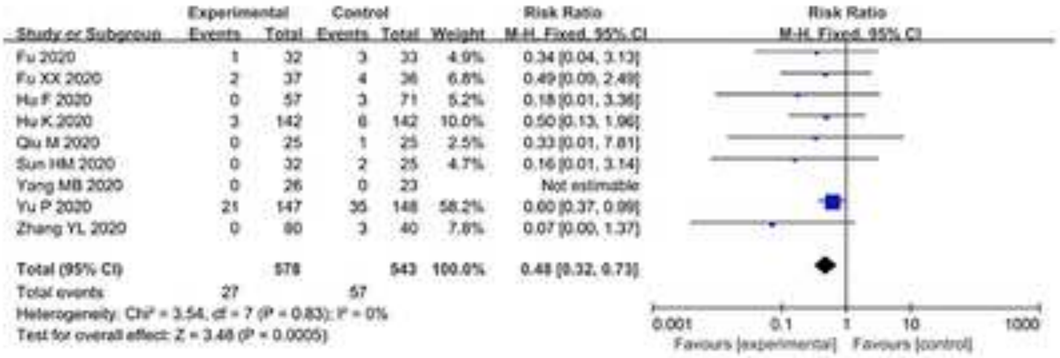

Fig 3c. Rate of conversion to severe cases.

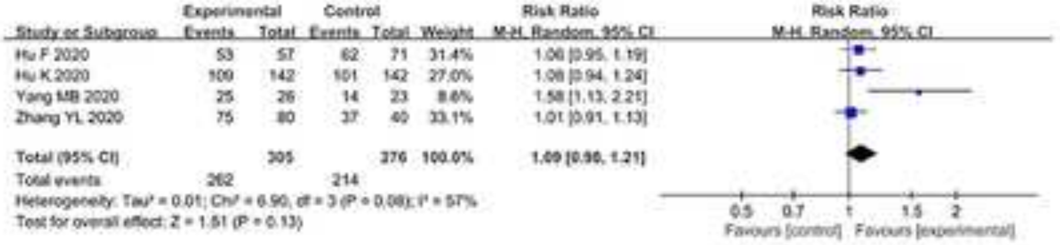

Fig 3d. Viral nucleic acid testing.

Fig 3. Clinical efficacy. Fig 3a. Lung CT. Fig 3b. Clinical cure rate. Fig 3c. Rate of conversion to severe cases. Fig 3d. Viral nucleic acid testing.

Figure Click here to access/download;Figure;Fig 4.tif

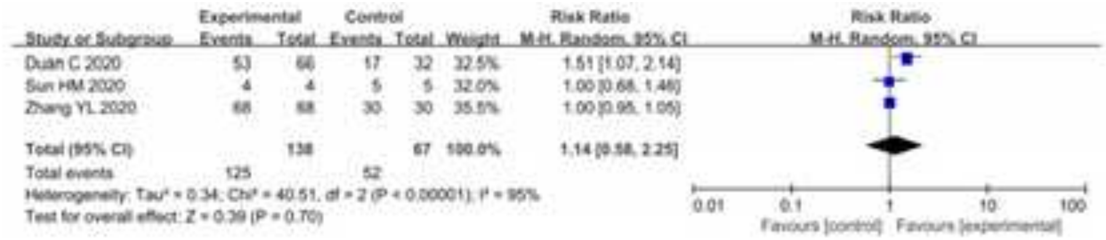

Fig 4a. Fever reduction cases.

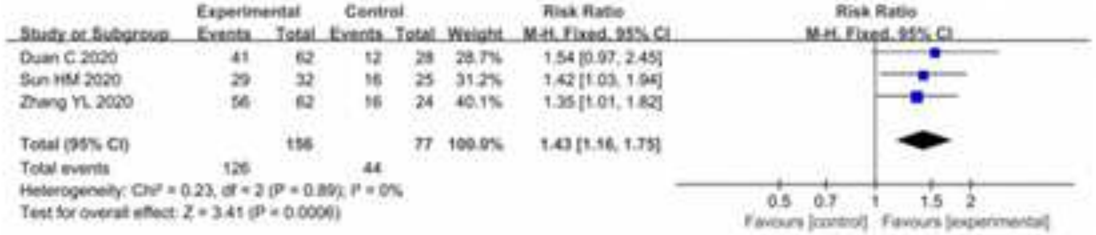

Fig 4b. Cough reduction cases.

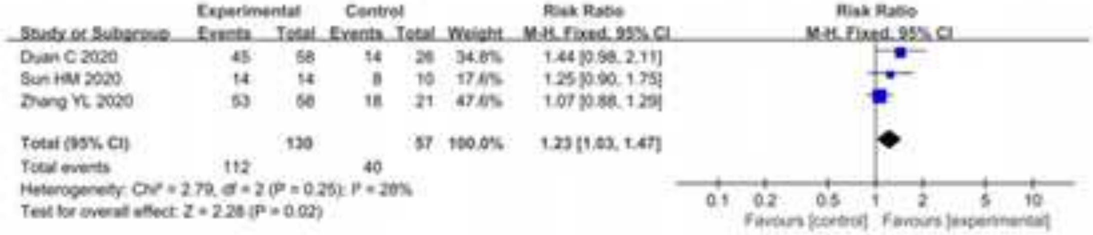

Fig 4c. Fatigue reduction cases.

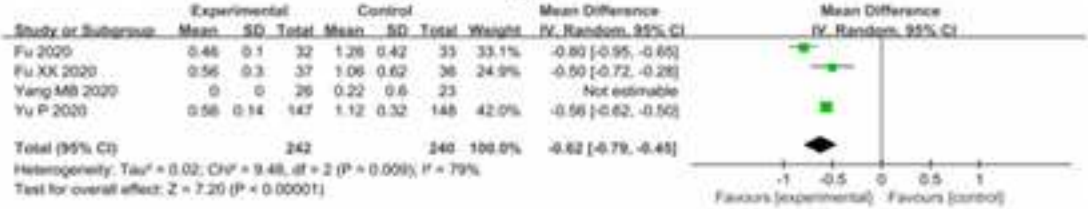

Fig 4d. TCM symptom score of fever.

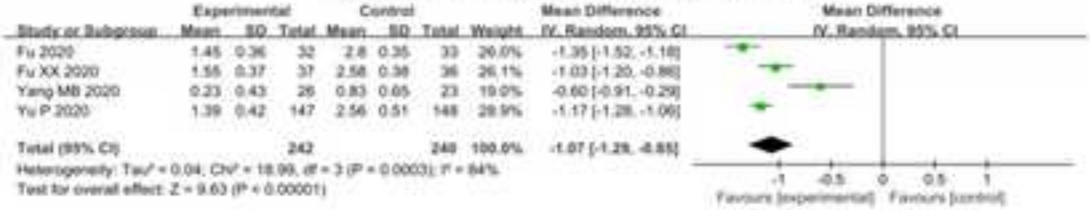

Fig 4e. TCM symptom score of cough.

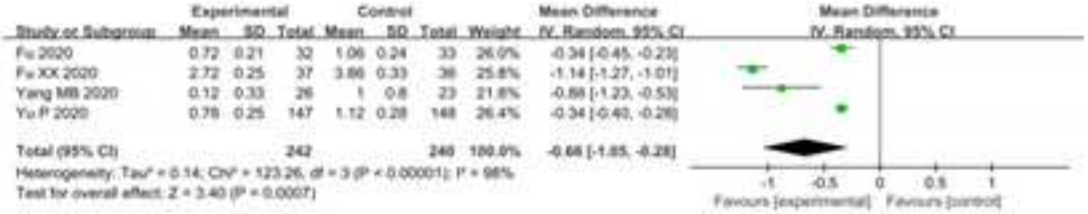

Fig 4f. TCM symptom score of fatigue.

Fig 4. Clinical symptoms. Fig 4a. Fever reduction cases. Fig 4b. Cough reduction cases. Fig 4c. Fatigue reduction cases. Fig 4d. TCM symptom score of fever. Fig 4e. TCM symptom score of cough. Fig 4f. TCM symptom score of fatigue.

Figure Click here to access/download;Figure;Fig 5.tif

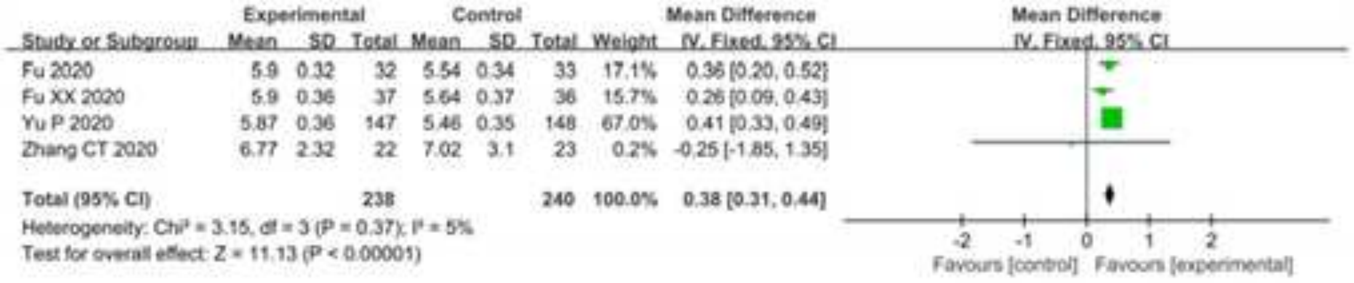

Fig 5a. WBC count.

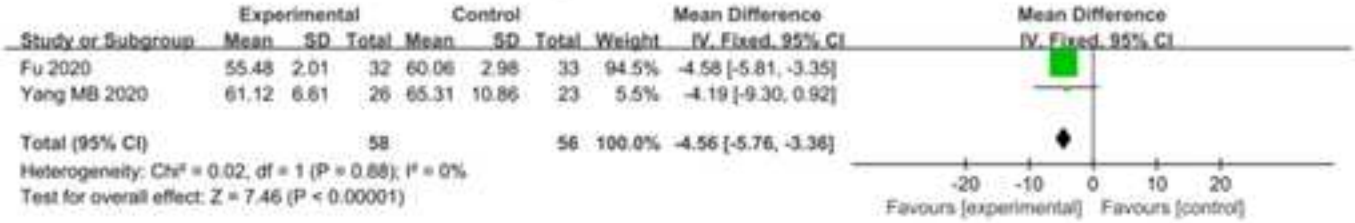

Fig 5b. NEU percentage.

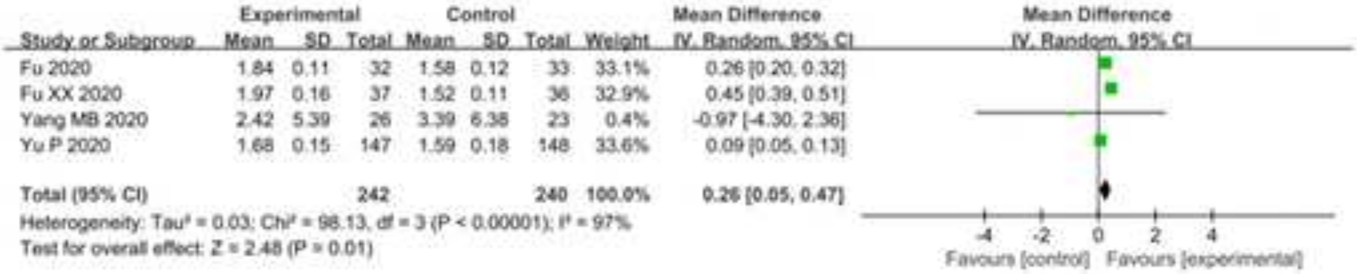

Fig 5c. LYM count.

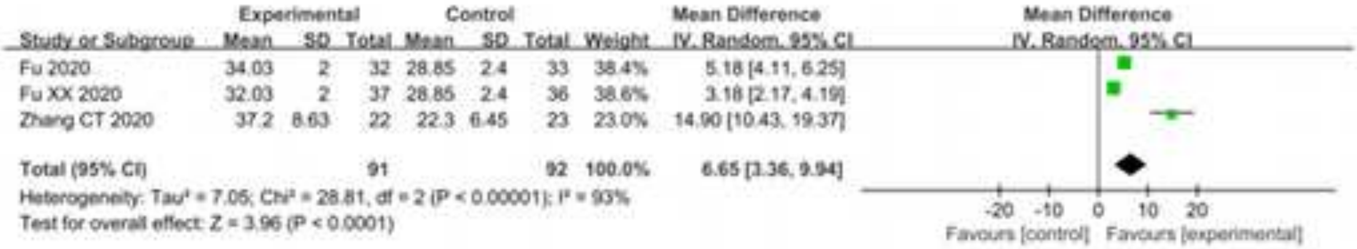

Fig 5d. LYM percentage.

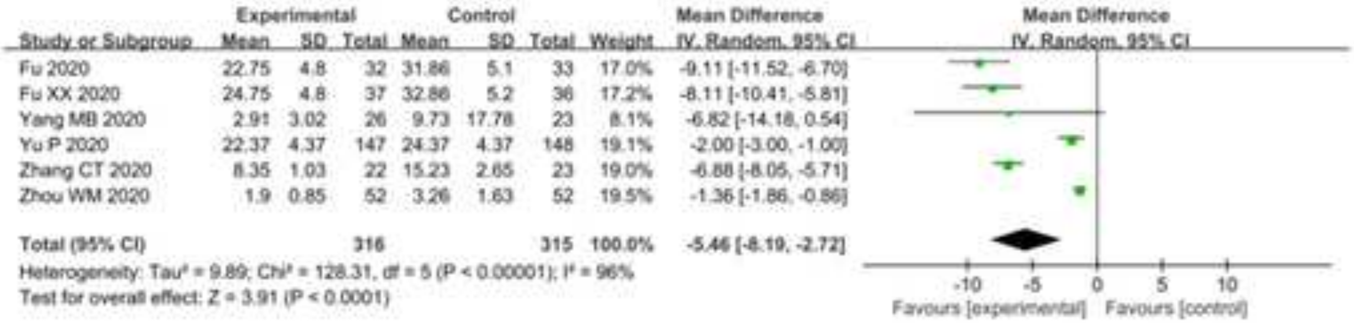

Fig 5e. CRP.

Fig 5. Inflammatory biomarkers. Fig 5a. WBC count. Fig 5b. NEU percentage. Fig 5c. LYM count. Fig 5d. LYM percentage. Fig 5e. CRP.

Figure
Click here to access/download;Figure;Fig 6.tif

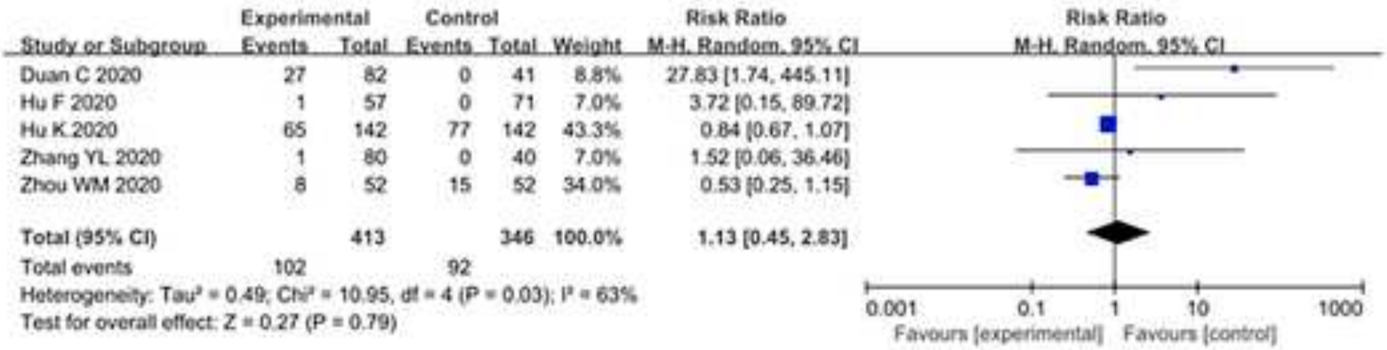

Fig 6a. Total number of adverse events cases.

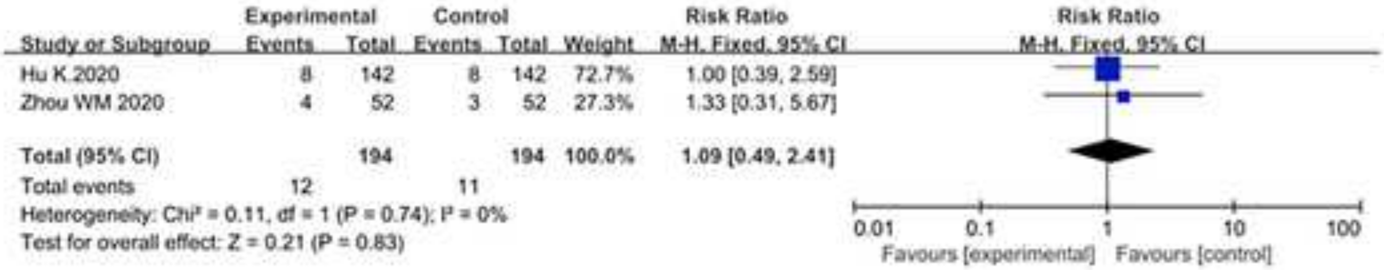

Fig 6b. Nausea and vomiting.

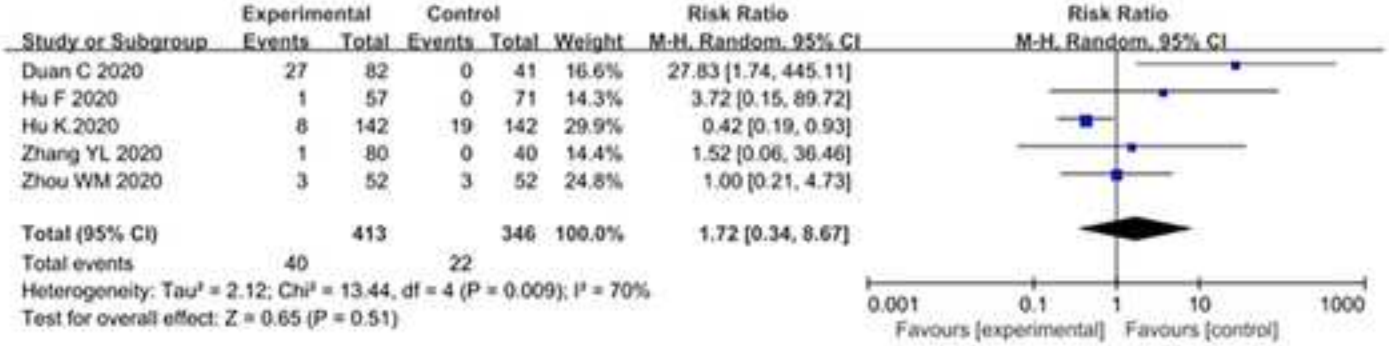

Fig 6c. Diarrhea.

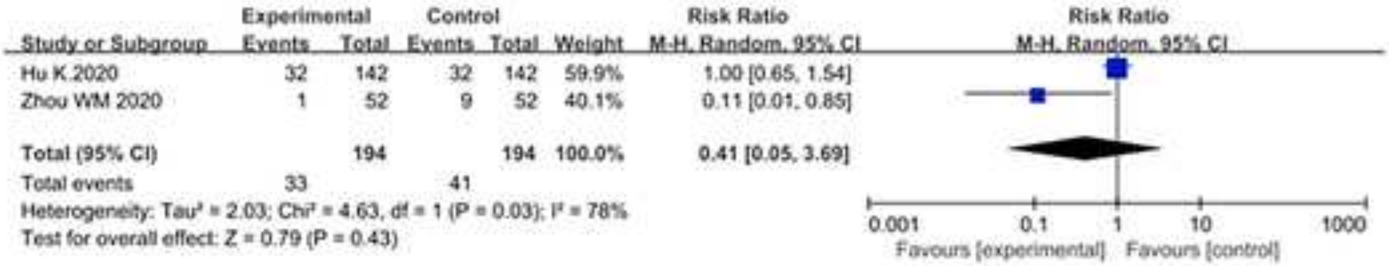

Fig 6d. Abnormal liver function.

Fig 6. Adverse events. Fig 6a. Total number of adverse events cases. Fig 6b. Nausea and vomiting. Fig 6c. Diarrhea. Fig 6d. Abnormal liver function.

 Click here to access/download;Figure;Fig 7.tif

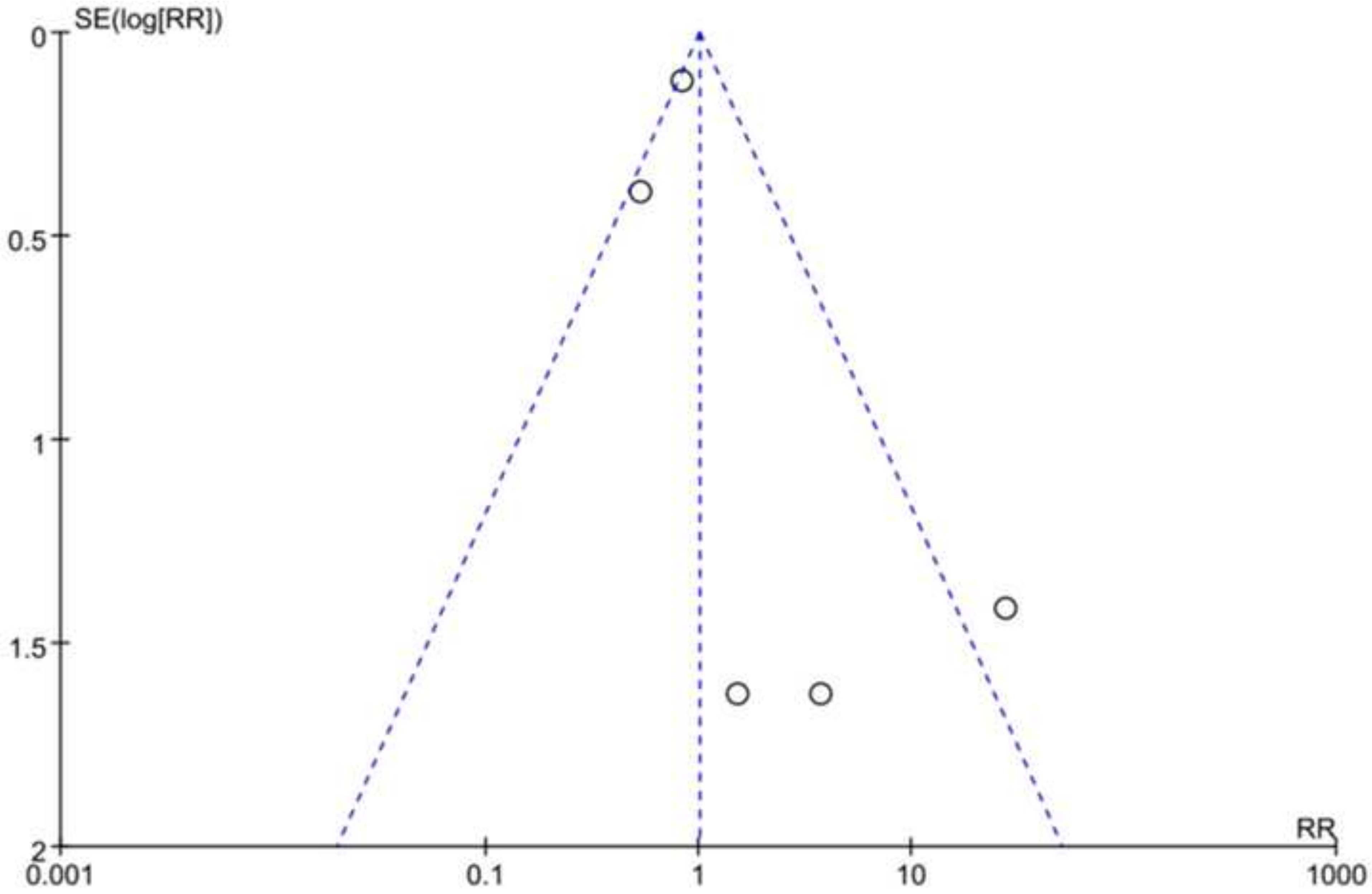

Fig 7. Adverse drug events trials.

Click here to access/download
Supporting Information
data set.xlsx

Click here to access/download
Supporting Information
S1_File.pdf

Chinese herbal medicine in adults with mild to moderate COVID-19: A systematic review and meta-analysis

Xuqin Du[1,2], Lipeng Shi [3,*], Wenfu Cao [1,2,4,*] , Biao Zuo[1,2], Aimin Zhou[3]

[1] College of Traditional Chinese Medicine, Chongqing Medical University, Chongqing 400016, People's Republic of China

[2] Chongqing Key Laboratory of Traditional Chinese Medicine for Prevention and Cure of Metabolic Diseases, Chongqing 400016, People's Republic of China

[3] Department of Cardiovascular Unit, Traditional Chinese medicine hospital Dianjiang Chongqing, Chongqing 408300, People's Republic of China

[4] Department of Chinese Traditional Medicine, The First Affiliated Hospital of Chongqing Medical University, Chongqing 400016, People's Republic of China

* Corresponding author

E-mail: shilipeng0206@163.com(LPS); caowenfu9316@163.com(WFC)

Abstract

Introduction

Coronavirus disease 2019 (COVID-19) caused by severe acute respiratory syndrome coronavirus 2 (SARS-CoV-2) has spread all over the world, which is a serious threat to human life and health. In China's experience in fighting COVID-19, traditional Chinese medicine (TCM), especially Chinese herbal medicine (CHM), has played an important role. Human studies reported the beneficial effects of CHM in the treatment of adult patients with mild to moderate COVID-19. Presently there is no systematic evaluation of the clinical efficacy of CHM in adult patients with mild to moderate COVID-19. Therefore, this review was designed to evaluate the efficacy and safety of CHM in the treatment of adult patients with mild to moderate COVID-19.

Methods

Randomized controlled trials (RCTs) on Chinese herbal medicine for mild to moderate COVID-19 were searched in the following eight electronic databases: PubMed, EMBASE, Cochrane Central Register of Controlled trials, the Clinical Trials.gov website, China National Knowledge Infrastructure (CNKI), China Science and Technology Journal Database (VIP), Wanfang Database and China Biology Medicine (CBM) from December 2019 to November 2020. Two reviewers independently searched, selected studies, and extracted data according to the eligibility criteria. Cochrane Risk of Bias (ROB) tool was used to assess the methodological quality of the included RCTs. Revman 5.3.0 software was used for statistical analysis.

Results

Twelve eligible RCTs were included with a total sample size of 1393. Our meta-analyses found that lung CT [RR=1.26, 95%CI (1.15, 1.38), $P < 0.00001$], and clinical cure rate [RR=1.26, 95%CI (1.16, 1.38), $P < 0.00001$] of CHM combined with conventional therapy in the treatment of mild to moderate COVID-19 was better than that of conventional therapy. The rate of conversion to severe cases [RR=0.48, 95%CI (0.32, 0.73), P=0.0005], TCM symptom score of fever [MD=-0.62, 95%CI (-0.79, -0.45), $P < 0.00001$], cough cases [RR=1.43, 95%CI (1.16, 1.75), P=0.0006], TCM symptom score of cough[MD=-1.07, 95%CI (-1.29, -0.85), $P < 0.00001$], TCM symptom score of fatigue[MD=-0.66, 95%CI (-1.05, -0.28), P=0.0007], and CRP[MD=-5.46, 95%CI (-8.19, -2.72), $P < 0.0001$] of CHM combined with conventional therapy was significantly lower than that of conventional therapy. The WBC count was significantly higher than that of conventional therapy[MD=0.38, 95%CI (0.31, 0.44), $P < 0.00001$]. Our meta-analysis results were robust and reliable through sensitivity analysis.

Conclusion

Chinese herbal medicine combined with conventional therapy could be effective and safe in the treatment of adults with mild to moderate COVID-19. More high-quality RCTs are needed in the future.

Introduction

Coronavirus disease 2019 (COVID-19) is caused by severe acute respiratory syndrome coronavirus 2 (SARS-CoV-2) [1]. It has the main symptoms of fever, cough, and fatigue [2]. COVID-19 has emerged as a global pandemic since its outbreak in Wuhan, China, in December 2019 [1]. As of March 25, 2021, more than 124.21 million confirmed cases and more than 2.73 million deaths had been reported globally [3]. COVID-19 has developed into a global public health emergency. Therefore, it is an urgent task to control COVID-19 effectively.

In China's experience fighting COVID-19, traditional Chinese medicine (TCM), especially Chinese herbal medicine (CHM), has played an important role [4]. CHM is a special medicine used in the prevention and treatment of diseases in TCM, which is composed of plant medicine, animal medicine, and mineral medicine [5]. A large number of epidemiological investigations showed that mild to moderate COVID-19 accounted for the largest proportion of cases [6]. The current conventional therapy recommendations for mild to moderate COVID-19 are mainly antiviral and symptomatic support treatment [7]. The recommended antiviral drugs are interferon, ribavirin, lopinavir-ritonavir, and chloroquine phosphate, which have been proved beneficial for COVID-19 [7-8]. Many trials have shown that, compared with conventional therapy, CHM has better effects for COVID-19 [9-10].

In our review, randomized controlled trials (RCTs) on CHM in the treatment of adult patients with mild to moderate COVID-19 were searched. The efficacy and safety of CHM in adults with mild to moderate COVID-19 were objectively evaluated by systematic evaluation and meta-analysis.

Methods

This review was conducted based on the Preferred Reporting Items for Systematic Reviews and Meta-Analyses (PRISMA) [11]. The protocol for our review has been registered on the International Prospective Register of Systematic Reviews (PROSPERO) with the registration number CRD42020213528.

Eligibility criteria

Inclusion and exclusion criteria. The diagnostic criteria of mild to moderate COVID-19 refer to " Diagnosis and Treatment Guideline for COVID-19 (Trial 8th Edition) " [7]. Mild COVID-19 is defined as mild clinical symptoms (such as low fever, mild fatigue, impairment of smell and taste, etc.) with no radiographic evidence of pneumonia [7]. Moderate COVID-19 is defined as having fever, respiratory symptoms, and imaging manifestations of pneumonia [7].

Inclusion criteria: (1) Types of studies: randomized controlled trials (RCTs). (2) Types of participants: adult patients (aged≥18 years) diagnosed as mild to moderate COVID-19. (3) Types of interventions: the treatment group was treated with a combination of CHM and conventional therapy. The administration of CHM was limited to oral administration. Patients in the control group were treated with conventional therapy. (4) Types of outcome measures: a. clinical efficacy (e.g. lung computed tomography (CT), clinical cure rate, rate of conversion to severe cases, viral nucleic acid testing), b. clinical symptoms (e.g. fever, cough, fatigue), c. inflammatory biomarkers (e.g. white blood cell (WBC) count, lymphocyte (LYM) count, LYM percentage, neutrophils (NEU) percentage, C-reactive protein (CRP)), d. adverse drug events (e.g. nausea and vomit, diarrhea, liver damage).

Exclusion criteria: (1) Patients with suspected diagnosis of COVID-19; (2) Retrospective studies, observational studies, repeated data studies, and cross-over studies.

Search strategy

RCTs assessing the efficacy and adverse events of CHM for adults with mild to moderate COVID-19 were searched in the following eight electronic databases: PubMed, EMBASE, Cochrane Central Register of Controlled Trials, the Clinical Trials.gov website, China National Knowledge Infrastructure (CNKI), China Science and Technology Journal Database (VIP), Wanfang Database and China Biology Medicine (CBM) from December 2019 to March 2021. There was no language restriction in our review. The search terms included "coronavirus disease 2019", "COVID-19", "novel coronavirus pneumonia", "SARS-CoV-2", "2019-nCoV", "traditional Chinese medicine", "Chinese herbal medicine", "Chinese herb", "Chinese herb therapy", "Chinese herbal formulas", "clinical trial", "randomized controlled trial", "randomised controlled trial", and "lin chuang yan jiu". Potential eligible trials were obtained by manually searching the reference list of previously published reviews. If possible, the conference abstracts were reviewed to find unpublished trials, and the data was obtained by contacting the author.

The PubMed search strategy is as follows. Search: ((((((coronavirus disease 2019) OR (COVID-19)) OR (novel coronavirus pneumonia)) OR (SARS-CoV-2)) OR (2019-nCoV)) AND (((((traditional Chinese medicine) OR (Chinese herbal medicine)) OR (Chinese herb)) OR (Chinese herb therapy)) OR (Chinese herbal formulas))) AND ((((clinical trial) OR (randomized controlled trial)) OR (randomised controlled trial)) OR (lin chuang yan jiu))

Study selection and data extraction

In the process of study selection, one reviewer (XQD) independently screened the literature from eight databases according to the eligibility criteria. Duplicate publications were removed. Through reading the title, abstract and full text, the reviewer (XQD) excluded non randomized controlled trials (non-RCTs) and irrelevant trials. The data were extracted independently by two reviewers (XQD and LPS) using

a pre-designed test form in duplicate. The following information was extracted from the included RCTs: basic characteristics (e.g. the title, first authors' name, publication date), participant characteristics (e.g. age, gender, sample size), intervention details (e.g. description of interventions, description of controls, dose, route of oral administration, duration of treatment), and outcome measures, as well as any adverse events. Reviewers (XQD and LPS) cross-checked the data. Any differences of opinion among the primary reviewers were resolved by a third reviewer (WFC). All reviewers were unbiased and had no conflicting interests.

Assessment of methodological quality

The methodological quality of the included RCTs was independently assessed by two reviewers (XQD and LPS) using the Cochrane Collaboration's tool [12]. Seven items of risk of bias (ROB) including adequate sequence generation, concealment of allocation, blinding (patient, investigator and assessor), incomplete outcome data addressed, free of selective reporting, and other biases were evaluated. Each item of ROB was assessed to be low ROB, high ROB, or unclear ROB. Additionally, any disagreements of ROB were resolved by consultation with the third reviewer (WFC).

Meta-analyses

Revman 5.3.0 software (The Cochrane Collaboration, Copenhagen, Denmark) was used for quantitative analysis. The relative risk (RR) was adopted for dichotomous variables. Mean difference (MD) or standard mean difference (SMD) were adopted for continuous variables. Confidence intervals (CIs) were set as 95% with $P < 0.05$ considered as statistically significant difference. Heterogeneity was assessed with the $\chi 2$ test and the I^2 statistical value. When the $P \geq 0.10$ or $I^2 \leq 50\%$, a fixed-effect model was adopted. Otherwise, a random-effect model was applied. We conducted a subgroup analysis of lung CT after 7 days of treatment duration. Sensitivity analysis was performed by leave-one-out method [13]. Funnel plot analysis was performed to evaluate the reporting bias for outcome measures with more than 10 RCTs [14].

Results

Eligible studies

The flow diagram of study selection and identification is showed in (Fig 1). The characteristics of included RCTs are listed in (Table 1). In this review, a total of twelve eligible RCTs were included [15-26]. Among the twelve RCTs [15-26], three were multi-centered trials [18,19,22] and the remaining nine were single-centered trials. All twelve RCTs were conducted in mainland China in 2020. One RCT was published online in English [19], and the rest were reported online in Chinese. The sample size of the included RCTs ranged from 45 to 295 (total 1393). The treatment duration varied from 5 to 15 days. Seven RCTs [16,19-24] described the lung CT. Five RCTs [16-17,19,22,26] described the clinical cure rate. Nine RCTs [16-23,25] described the rate of conversion to severe cases. Four RCTs [18-19,22,25] described the viral nucleic acid testing. Clinical symptoms of fever, cough and fatigue were described in seven RCTs [15-17,21-23,25], of which three RCTs [15,21,25] described fever/cough/fatigue reduction cases, and four RCTs [16-17,23-24] described TCM symptom score of fever/cough/fatigue. Inflammatory biomarkers were described in six RCTs [16-17,22-24,26], of which four RCTs [16-17,23-24] described WBC count, four RCTs [16-17,22-23] described LYM count, three RCTs [16-17,24] described LYM percentage, two RCTs [16,22] described NEU percentage, and six RCTs [16-17,22-24,26] described CRP. Adverse drug events were described in ten RCTs [15-19,22-26].

Table 1. The characteristics of included RCTs.

First author	Type of COVID-19	Sample size (M/F)	Age (yrs)	Intervention
Duan C[15]	mild	T: 82 (39/ 43) C: 41(23/18)	T: 51.99±13.88 C: 50.29±13.17	Jinhua Qinggan granule therapy

Fu[16]	mild/moderate	T: 32 (17/ 15) C: 33(19/14)	T: 43.26±7.15 C:43.68±6.45	Toujie Quwen granule + therapy
Fu XX[17]	moderate	T: 37 (19/18) C: 36(19/17)	T: 45.26 ± 7.25 C:44.68 ± 7.45	Toujie Quwen granule + therapy
Hu F[18]	moderate	T: 100 (49/ 51) C: 100(55/45)	T: 47.00±14.06 C: 49.28±11.14	Jinyinhua oral liquid + c
Hu K[19]	mild/moderate	T: 142 ((79/63) C: 142(71/71)	T:50.4 ± 15.2 C:51.8 ± 14.8	Lianhua Qingwen capsu therapy
Qiu M[20]	moderate	T: 25 (13/ 12) C: 25(14/11)	T: 53.35±18.35 C:51.32 ±14.62	Maxing Xuanfei Jiedu D conventional therapy
Sun HM[21]	mild/moderate	T: 32 (17/ 15) C: 25(11/14)	T: 45.4±14.10 C:42.0±11.70	Lianhua Qingke granule therapy
Yang MB[22]	moderate	T: 26(16/ 10) C: 23(9/14)	T: 50.35±13.37 C:47.17 ±16.57	Reyanning mixture + co
Yu P[23]	mild/moderate	T: 147 (82/65) C: 148(89/59)	T: 48.27±9.56 C:47.25±8.67	Lianhua Qingwen granu therapy
Zhang CT[24]	moderate	T: 22 (9/ 13) C: 23 (10/13)	T: 53.7 ± 3.5 C: 55.6 ± 4.2	Jiawei Dayuan Decoctio therapy
Zhang YL[25]	moderate	T: 80 (50/ 30) C: 40(23/17)	T: 53.4±13.70 C:52.0±14.10	Jinyinhua oral liquid + c
Zhou WM[26]	moderate	T: 52 (32/ 20) C: 52(28/24)	T: 52.47±10.99 C:51.11 ±9.87	diammonium glycyrrhizi therapy

Assessment of methodological quality

The methodological quality of the included RCTs was assessed according to the Cochrane Collaboration's tool [12]. As shown in (Fig 2a) and (Fig 2b), green and "+" indicate "Low risk"; yellow and "?" indicate "Unclear". Detailed information on sequence generation of randomization was described in ten trials (10/12, 83.33%) [15-23,26]. Detailed information on allocation concealment was unclear. One RCT reported blinding of the assessor [19]. Detailed information on blinding of patient, investigator, and assessor was not described in the rest eleven RCTs. Attrition bias was scored as 100% low risk. Detailed information on selective reporting was unclear.

Description of CHM

The components of CHM are listed in (Table 2). Nine oral CHM were used in this review, including Jinhua Qinggan granule [15], Toujie Quwen granule [16-17], Jinyinhua oral liquid [18,25], Lianhua Qingwen capsule (granule) [19,23], Maxing Xuanfei Jiedu Decoction [20], Lianhua Qingke granule [21], Reyanning mixture [22], Jiawei Dayuan Decoction [24], diammonium glycyrrhizinate [26]. Among the nine oral CHM, the most frequently used Chinese medicine was honeysuckle, which was used in seven trials (58.33%) [15-19,23,25], followed by forsythia (50.00%) [15-17,19,21,23], and ephedra (50.00%) [15,19-21,23-24].

Four dosage formulations of oral CHM were included in this review, including granule [15-17,21,23-24], oral liquid [18,22,25], capsule [19,26], and decoction [20]. Among the four dosage formulations of oral CHM, the most frequently used Chinese medicine was granule, which was used in six trials (50.00%) [15-17,21,23-24].

Table 2. The components of CHM.

References	CHM	Components

Duan C[15]	Jinhua Qinggan granule	Jinyinhua 10g, Shigao 10g, Mahuang(processed with honey) 10g, Kuxingren(stir-frying) 10g, Huangqin 10g, Lianqiao 10g, Zhebeimu 10g, Zhimu 10g, Niubangzi 10g, Qinghao 10g, Bohe 10g, Gancao10g
Fu[16]	Toujie Quwen granule	Lianqiao 30 g，Shancigu 20 g，Jinyinhua 15 g，Huangqin 10 g， Daqingye 10 g ,Chaihu 5 g ,Qinghao 10 g ,Chantui 10 g，Qianhu 5 g，Chuanbeimu 10 g，Zhebeimu 10 g，Wumei 30 g ,Xuanshen 10 g， Huangqi 45 g ,Fuling 30 g，Taizishen 15 g
Fu XX[17]	Toujie Quwen granule	Lianqiao 30 g，Shancigu 20 g，Jinyinhua 15 g，Huangqin 10 g， Daqingye 10 g ,Chaihu 5 g ,Qinghao 10 g ,Chantui 10 g，Qianhu 5 g，Chuanbeimu 10 g，Zhebeimu 10 g，Wumei 30 g ,Xuanshen 10 g， Huangqi 45 g ,Fuling 30 g，Taizishen 15 g
Hu F[18]	Jinyinhua oral liquid	Jinyinhua 5.4g
Hu K[19]	Lianhua Qingwen capsule	Lianqiao, Jinyinhua, Mahuang(stir-frying), Kuxingren(stir-frying), Shigao, Banlangen, Guanzhong, Yuxingcao, Huoxiang, Dahuang, Hongjingtian, Bohe, Gancao
Qiu M[20]	Maxing Xuanfei	Mahuang 9 g ,Kuxingren 12 g ,Shigao 15~30 g ,Zhebeimu 12 g， Chantui 10 g，Jiangchan 15 g，Jianghuang 12 g，

	Jiedu Decoction	Jiegeng 12 g，Zhiqiao 12 g，Caoguo 9 g，Caodoukou 12 g
Sun HM[21]	Lianhua Qingke granule	Mahuang, Sangbaipi, Kuxingren(stir-frying), Lianqiao, mountain honeysuckle, Dahuang
Yang MB[22]	Reyanning mixture	Pugongying, Huzhang, Baijiang Herba cum Radice, Banzhilian
Yu P[23]	Lianhua Qingwen granule	Lianqiao, Jinyinhua, Mahuang(stir-frying), Kuxingren(stir-frying), Shigao, Banlangen, Guanzhong, Yuxingcao, Huoxiang, Dahuang, Hongjingtian, Bohe, Gancao
Zhang CT[24]	Jiawei Dayuan Decoction	Mahuang(stir-frying) 10 g, Xingren 15 g, crude gypsum 20 g, trichosanthes bark 20 g, Dahuang(Stir-fry with yellow rice wine) 6 g, Tinglizi 10g, Taoren 10 g, Caoguo 6 g, Binglang 10 g, Cangzhu 10 g
Zhang YL[25]	Jinyinhua oral liquid	Jinyinhua 5.4g
Zhou WM[26]	diamine glycyrrhizinate	diamine glycyrrhizinate

Efficacy and safety assessment

Clinical efficacy

Clinical efficacy was reported in eleven RCTs [16-26], of which seven RCTs [16,19-24] reported lung CT, five RCTs [16-17,19,22,26] reported clinical cure rate, nine RCTs [16-23,25] reported rate of conversion to severe cases, and four RCTs [18-19,22,25] reported viral nucleic acid testing. Evaluation criteria for lung CT refer to COVID-19 Guidelines for Imaging Assisted Diagnosis [27]. Clinical cure standards refer to Guiding Principles for Clinical Research of New Chinese Materia Medica [28]. The therapeutic effects are classified as effective, improved, and ineffective. Clinical cure rate = (effective cases + improved cases) / total cases × 100%.

In the field of lung CT, 426 patients were in the treatment group and 419 in the control group. In the field of clinical cure rate, 410 patients were in the treatment group and 411 in the control group. In the field of rate of conversion to severe cases, 578 patients were in the treatment group and 543 in the control group. In the field of viral nucleic acid testing, 305 patients were in the treatment group and 276 in the control group.

According to lung CT, meta-analysis and subgroup analysis results are shown in (Fig 3a). In terms of lung CT, Chi^2 test shows that I^2=8% ($<$50%), P=0.37. In terms of lung CT after 7 days of treatment duration, Chi^2 test shows that I^2=44% ($<$50%), P=0.17. In terms of lung CT of 10 to 14 days treatment duration, Chi^2 test shows that I^2=0% ($<$50%), P=0.87. It shows that there is no heterogeneity between the included trials, so we choose to use fixed-effect model for meta-analysis and subgroup analyses. It can be seen from the forest plot that compared with conventional therapy, CHM combined with conventional therapy exhibited a significant improvement on lung CT [7 trials, n=845, RR=1.26, 95%CI (1.15, 1.38), $P<$0.00001] (Fig 3a). Subgroup analysis revealed an improvement on lung CT after 7 days of treatment duration by CHM combined with conventional therapy [n=845, RR=1.18, 95%CI (1.02, 1.36), P=0.03] (Fig 3a); a significant improvement on lung CT of 10 to 14 days treatment

duration by CHM combined with conventional therapy [n=845, RR=1.34, 95%CI (1.19, 1.50), $P<0.00001$] (Fig 3a).

According to clinical cure rate and rate of conversion to severe cases, meta-analysis results are shown in (Fig 3b) and (Fig 3c). In terms of clinical cure rate, Chi^2 test shows that $I^2=0\%$ ($<50\%$), $P=0.77$. In terms of rate of conversion to severe cases, Chi^2 test shows that $I^2=0\%$ ($<50\%$), $P=0.83$. It shows that there is no heterogeneity between the included trials, so we choose to use fixed-effect model for analyses. It can be seen from the forest plot that compared with conventional therapy, CHM combined with conventional therapy exhibited a significant improvement on clinical cure rate [5 trials, n=821, RR=1.26, 95%CI (1.16, 1.38), $P<0.00001$] (Fig 3b); a significant reduction in rate of conversion to severe cases [9 trials, n=1121, RR=0.48, 95%CI (0.32, 0.73), $P=0.0005$] (Fig 3c).

According to viral nucleic acid testing, meta-analysis result is shown in (Fig 3d). Chi^2 test shows that $I^2=57\%$ ($>50\%$), $P=0.08$. It shows that there is heterogeneity between the included trials, so we choose to use random-effects model for analysis. It can be seen from the forest plot that no statistical difference in viral nucleic acid testing was identified between the treatment group and the control group [4 trials, n=581, RR=1.09, 95%CI (0.98, 1.21), $P=0.13$] (Fig 3d).

Clinical symptoms

Clinical symptoms of fever, cough and fatigue was reported in seven RCTs [15-17,21-23,25]. Among them, three RCTs [15,21,25] reported number of fever/cough/fatigue reduction cases, and four RCTs [16-17,22-23] reported TCM symptom score of fever/cough/fatigue.

In the field of fever reduction cases, 138 patients were in the treatment group and 67 in the control group. In the field of cough reduction cases, 156 patients were in the treatment group and 77 in the control group. In the field of fatigue reduction cases, 130 patients were in the treatment group and 57 in the control group. In the field of

TCM symptom score of fever/cough/fatigue, there were 242 patients in the treatment group and 240 in the control group.

According to fever reduction cases, cough reduction cases, and fatigue reduction cases, meta-analysis results are shown in (Fig 4a), (Fig 4b), and (Fig 4c). In terms of fever reduction cases, Chi^2 test shows that I^2=95% (>50%), P<0.00001. It shows that there is heterogeneity between the included trials, so we choose to use random-effects model for analysis. It can be seen from the forest plot that no statistical difference in number of fever reduction cases was identified between the treatment group and the control group [3 trials, n=205, RR=1.14, 95%CI (0.58, 2.25), P=0.70] (Fig 4a). In terms of cough reduction cases, Chi^2 test shows that I^2=0% (< 50%), P=0.89. It shows that there is no heterogeneity between the included trials, so we choose to use fixed-effect model for analysis. It can be seen from the forest plot that compared with conventional therapy, CHM combined with conventional therapy revealed a significant reduction in cough cases [3 trials, n=205, RR=1.43, 95%CI (1.16, 1.75), P=0.0006] (Fig 4b). In terms of fatigue reduction cases, Chi^2 test shows that I^2=28% (<50%), P=0.25. It shows that there is no heterogeneity between the included trials, so we choose to use fixed-effect model for analysis. It can be seen from the forest plot that CHM combined with conventional therapy revealed a significant reduction in fatigue cases [3 trials, n=205, RR=1.23, 95%CI (1.03, 1.47), I^2=28%, P=0.02] (Fig 4c).

According to TCM symptom score of fever, cough, and fatigue, meta-analysis results are shown in (Fig 4d), (Fig 4e), and (Fig 4f). In terms of TCM symptom score of fever, Chi^2 test shows that I^2=79% (>50%), P=0.009. In terms of TCM symptom score of cough, Chi^2 test shows that I^2=84% (>50%), P=0.0003. In terms of TCM symptom score of fatigue, Chi^2 test shows that I^2=98% (>50%), P<0.00001. It shows that there is heterogeneity between the included trials, so we choose to use random-effects model for analyses. It can be seen from the forest plot that compared with conventional therapy, CHM combined with conventional therapy revealed a significant reduction in TCM symptom score of fever [4 trials, n=482, MD=-0.62,

95%CI (-0.79, -0.45), $P < 0.00001$] (Fig 4d); a significant reduction in TCM symptom score of cough [4 trials, n=482, MD=-1.07, 95%CI (-1.29, -0.85), $P < 0.00001$] (Fig 4e); a significant reduction in TCM symptom score of fatigue [4 trials, n=482, MD=-0.66, 95%CI (-1.05, -0.28), P=0.0007] (Fig 4f).

Inflammatory biomarkers

Inflammatory biomarkers were reported in six RCTs [16-17,22-24,26], of which four RCTs [16-17,23-24] reported WBC count, three RCTs [16-17,24] reported LYM percentage, four RCTs [16-17,22-23] reported LYM count, two RCTs [16,22] reported NEU percentage, and six RCTs [16-17,22-24,26] reported CRP.

In the field of WBC count, 238 patients were in the treatment group and 240 in the control group. In the field of LYM count, 242 patients were in the treatment group and 240 in the control group. In the field of LYM percentage, 91 patients were in the treatment group and 92 in the control group. In the field of NEU percentage, 58 patients were in the treatment group and 56 in the control group. In the field of CRP, 316 patients were in the treatment group and 315 in the control group.

According to WBC count and NEU percentage, meta-analysis results are shown in (Fig 5a) and (Fig 5b). In terms of WBC count, Chi^2 test shows that I^2=5% (<50%), P=0.37. In terms of NEU percentage, Chi^2 test shows that I^2=0% (<50%), P=0.88. It shows that there is no heterogeneity between the included trials, so we choose to use fixed-effect model for analyses. It can be seen from the forest plot that compared with conventional therapy, CHM combined with conventional therapy revealed a significant improvement on WBC count [4 trials, n=478, MD=0.38, 95%CI (0.31, 0.44), I^2=5%, $P < 0.00001$] (Fig 5a); a significant reduction in NEU percentage [2 trials, n=114, MD=-4.56, 95%CI (-5.76, -3.36), I^2=0%, $P < 0.00001$] (Fig 5b).

According to LYM count, LYM percentage, and CRP, meta-analysis results are shown in (Fig 5c), (Fig 5d), and (Fig 5e). In terms of LYM count, Chi^2 test shows that I^2=97% (>50%), $P < 0.00001$. In terms of LYM percentage, Chi^2 test shows that I^2=93% (>50%), $P < 0.00001$. In terms of CRP, Chi^2 test shows that I^2=96% (>

50%), $P<0.00001$. It shows that there is heterogeneity between the included trials, so we choose to use random-effects model for analyses. It can be seen from the forest plot that compared with conventional therapy, CHM combined with conventional therapy revealed a significant improvement on LYM count [4 trials, n=482, MD=0.26, 95%CI (0.05, 0.47), $P=0.01$] (Fig 5c); a significant improvement on LYM percentage [3 trials, n=183, MD=6.65, 95%CI (3.36, 9.94), $P<0.0001$] (Fig 5d); a significant reduction in CRP [6 trials, n=631, MD=-5.46, 95%CI (-8.19, -2.72), $P<0.0001$] (Fig 5e).

Adverse drug events

In this review, adverse drug events were reported in ten RCTs [15-19,22-26], while the remaining two RCTs [20-21] did not. Among ten RCTs [15-19,22-26], no adverse event was identified in either treatment or control groups [16-17,22-24]. Adverse drug events in the remaining five RCTs included gastrointestinal reactions (diarrhea, poor appetite, nausea, vomiting), headache, renal dysfunction, and abnormal liver function [15,18-19,25-26]. All reported adverse drug events were mild in the treatment and control groups, and were tolerable or alleviated after withdrawal.

In the field of total number of adverse drug events cases, 413 patients were in the treatment group and 346 in the control group. In the field of nausea and vomiting, 194 patients were in the treatment group and 194 in the control group. In the field of diarrhea, 413 patients were in the treatment group and 346 in the control group. In the field of abnormal liver function, 194 patients were in the treatment group and 194 in the control group.

According to total number of adverse drug events cases, meta-analysis result is shown in (Fig 6a). In terms of total number of adverse drug events cases, Chi^2 test shows that $I^2=63\%$ ($>50\%$), $P=0.03$. It shows that there is heterogeneity between the included trials, so we choose to use random-effects model for analysis. It can be seen from the forest plot that no statistical difference in total number of adverse drug events cases was identified between the treatment group and the control group [5

trials, n=759, RR=1.13, 95%CI (0.45, 2.83), *P*=0.79] (Fig 6a). According to nausea and vomiting, meta-analysis result is shown in (Fig 6b). In terms of nausea and vomiting, Chi^2 test shows that I^2=0% (<50%), *P*=0.74. It shows that there is no heterogeneity between the included trials, so we choose to use fixed-effect model for analysis. It can be seen from the forest plot that subgroup analysis revealed no statistical difference in nausea and vomiting [2 trials, n=388, RR=1.09, 95%CI (0.49, 2.41), *P*=0.83] (Fig 6b).

According to diarrhea and abnormal liver function, meta-analysis results are shown in (Fig 6c) and (Fig 6d). In terms of diarrhea, Chi^2 test shows that I^2=70% (>50%), *P*=0.009. In terms of abnormal liver function, Chi^2 test shows that I^2=78% (>50%), *P*=0.03. It shows that there is heterogeneity between the included trials, so we choose to use random-effects model for analyses. It can be seen from the forest plot that subgroup analysis revealed statistical difference in diarrhea [5 trials, n=759, RR=1.72, 95%CI (0.34, 8.67), *P*=0.51] (Fig 6c); and abnormal liver function [2 trials, n=388, RR=0.41, 95%CI (0.05, 3.69), *P*=0.43] (Fig 6d). Poor appetite, headache, and renal dysfunction were reported in one RCT [19], with no statistical difference identified between the treatment group and the control group.

Sensitivity analysis

Sensitivity analysis revealed that there was a small change in the effect amount, and was a significant difference in lung CT, clinical cure rate, rate of conversion to severe cases, TCM symptom score of fever, number of cough reduction cases, TCM symptom score of cough, TCM symptom score of fatigue, WBC count, and CRP, which indicated the above meta-analysis results to be robust and reliable.

Publication bias

In our study, ten trials [15-19,22-26] reported adverse drug events. Among ten trials [15-19,22-26], five trials [16-17,22-24] reported no adverse event identified in either treatment or control groups. The funnel plot was used to analyze the reported

adverse events trials to explore the bias (Fig 7). The funnel plot is symmetrical, indicating no obvious deviation.

Discussion

The clinical classification of COVID-19 is mild, moderate, severe, and critical [7]. Severe COVID-19 is more likely to have serious complications, such as shock, acute respiratory distress syndrome (ARDS), arrhythmia, and acute heart injury [29-30], all of which significantly increase the difficulty and cost of treatment. Therefore, it is of great significance to prevent COVID-19 from developing from mild or moderate to severe. In our study, it was found that compared with conventional therapy, mild to moderate COVID-19 patients treated with both CHM and conventional therapy had more benefit. Similar studies have shown that CHM has positive effects in COVID-19 patients [31-33]. Facing such a severe COVID-19 epidemic, Western countries should pay attention to the therapeutic effect of CHM for COVID-19.

According to the theory of TCM, epidemic disease refers to an acute infectious disease characterized by sudden onset, rapid transmission, dangerous condition, and strong infectivity after feeling pestilence evil [34]. COVID-19 belongs to the "epidemic disease" of TCM, in the light of its incidence mode and epidemic trend [7]. The pathogenesis of mild to moderate COVID-19 is dampness-heat or cold-dampness obstructing the lung [7]. Therefore, CHM with the effect of heat-clearing, eliminating dampness, resolving phlegm, and dispersing cold is widely used [7]. In the included studies, nine different oral CHM were used, including Lianhua Qingwen capsule (granule), Toujie Quwen granule, Jinyinhua oral liquid, diammonium glycyrrhizinate, etc. Lianhua Qingwen capsule is originated from classical Chinese herbal formulas, which can decrease influenza A virus (H1N1) replication, lung lesions, and inflammation [35]. Also, Lianhua Qingwen capsule may reduce lung injury and help eliminate SARS- CoV- 2 infection by regulating Akt1 [36]. One study has shown that Toujie Quwen granule may have therapeutic effects on COVID-19 by regulating

SARS- CoV- 2 infection, immune and inflammation-related targets, and pathways [37]. Diammonium glycyrrhizinate is used as a hepatic protector, which is the main component of licorice root extracts [38]. Diammonium glycyrrhizinate can decrease the serum ALT and AST levels and improve the histological damage, down-regulated the inflammatory cytokines, inhibited the apoptosis of T lymphocytes in the thymus [38].

Among the nine oral CHM, the most frequently used Chinese medicine was honeysuckle, followed by forsythia, and ephedra. Honeysuckle and forsythia have the function of clearing heat-toxicity and dispersing wind-heat in the theory of TCM [5]. Honeysuckle polysaccharide is an active component of honeysuckle, which can regulate non-specific immunity [39], inhibit the expression of inflammatory factors TNF-α and IL-1β [40], and inhibit a variety of viruses [41]. Phillyrin is an active component of forsythia, which has antiviral and anti-inflammatory activities [42-43]. Ephedra has the function of dissipating cold and diffusing the lung to calm panting in TCM theory [5]. Ephedrine is an active component of ephedra, which can increase the production of anti-inflammatory cytokines IL-10, reduce the production of pro-inflammatory cytokines TNF-α and IL-12 [44], and play an antiviral role by inhibiting viral replication [45].

Mild to moderate COVID-19 patients treated with both CHM and conventional therapy had better outcomes in the parameters including clinical efficacy, clinical symptoms, and inflammatory response. Our study found that compared with conventional therapy, CHM combined with conventional therapy can improve the scores of symptoms such as fever, cough, and fatigue, and reduce cough cases. This is related to CHM can affect the production of inflammatory cytokines [35,38]. Cytokine storm is perhaps one of the critical hallmarks of COVID-19 severity [46]. Cytokine storm is a hyperproduction of proinflammatory cytokines, which leads to ARDS aggravation and widespread tissue damage resulting in multi-organ failure [46-47]. In our study, we found that CHM combined with conventional therapy can increase WBC count, and reduce CRP. CHM combined with conventional therapy

had a better effect on improving lung CT, promoting clinical cure rate, and reducing rate of conversion to severe cases.

Due to different formulations and unclear composition, CHM has many unknown factors to be solved. In our study, we found that CHM formulations used in the treatment group are different, and quality of herbal intervention is unclear. CHM is likely to require a standard treatment. Besides, quality of herbal formula should be monitored through standardized. In this way, the best evidence can be systematically summarized to better provide an evidence-based basis for TCM decision-making. CHM treatment, which is based on individualized assessment, can be affected by different diet practices, and weather, resulting in its difficulty of using in western countries. Therefore, we think it is necessary for Western countries to hire TCM experts to participate in the treatment of COVID-19. Safety issues should be a concern when CHM is used for COVID-19. In our study, we found that most of the included trials reported adverse drug events. CHM combined with conventional therapy did not increase adverse drug events. The funnel plot of adverse drug events indicated no obvious deviation.

However, it was a common problem that most of the included trials had poor methodological design and that the merger statistical analysis of some outcomes had unexplained heterogeneity. More high-quality trials are needed in the future. Despite the poor methodology and the unexplained heterogeneity, our finding is very valuable and timely in view of no specific drugs approved for COVID-19.

Limitations

Despite the usefulness of our finding, this review also has several limitations that could be improved upon in future studies. First of all, most of the included trials had deficiencies in methodology design, including hidden allocation and inadequate reporting of blind methods. Secondly, the composition, dosage, and frequency of CHM were different in the treatment groups. Thirdly, the multicenter trials were

lacking. In addition, the duration of the included trials ranged from 5 to 15 days. Therefore, it is necessary to design more high-quality trials with a multicenter, large sample, and longer follow-up to better observe the efficacy and possible adverse events of CHM combined with conventional therapy in the treatment of adults with mild to moderate COVID-19.

Conclusion

Chinese herbal medicine combined with conventional therapy could be effective and safe in the treatment of adults with mild to moderate COVID-19. It can improve the clinical cure rate, main clinical symptoms, imaging and laboratory indexes, and reduce the rate of conversion to severe cases. However, due to the fact that COVID-19 is a sudden disease, it is difficult to carry out double-blind clinical trials. This leads to insufficient methodology of the existing-related trials. Therefore, more high-quality trials are needed to evaluate the efficacy and safety of Chinese herbal medicine combined with conventional therapy in the treatment of adults with mild to moderate COVID-19 in the future.

Author Contributions

Conceptualization: Lipeng Shi, Wenfu Cao.

Data curation: Xuqin Du, Lipeng Shi.

Formal analysis: Xuqin Du.

Funding acquisition: Xuqin Du.

Investigation: Xuqin Du, Lipeng Shi.

Methodology: Xuqin Du, Lipeng Shi.

Software: Xuqin Du.

Supervision: Wenfu Cao.

Validation: Biao Zuo, Aimin Zhou.

Writing – original draft: Xuqin Du.

Writing – review & editing: Xuqin Du, Lipeng Shi, Wenfu Cao, Biao Zuo, Aimin Zhou.

References

1. Sattler A, Angermair S, Stockmann H, et al. SARS-CoV-2-specific T cell responses and correlations with COVID-19 patient predisposition[J]. J Clin Invest, 2020,130(12):6477-6489.
2. Chen N, Zhou M, Dong X, et al. Epidemiological and clinical characteristics of 99 cases of 2019 novel coronavirus pneumonia in Wuhan, China: a descriptive study [J]. Lancet, 2020,395(10223):507-513.
3. World Health Organization. WHO Coronavirus (COVID-19) Dashboard.2021 [EB/OL]. https://covid19.who.int/(accessed Mar 26, 2021)
4. Ren JL, Zhang AH, Wang XJ. Traditional Chinese medicine for COVID-19 treatment[J]. Pharmacol Res, 2020, 155:104743.
5. Gao XM. Traditional Chinese Pharmacy (advanced series of traditional Chinese medicine) [M]. Beijing: People's Health Publishing House, 2000.
6. Wu ZY, McGoogan JM. Characteristics of and important lessons from the coronavirus disease 2019(COVID-19) outbreak in China[J]. J Am Med Assoc, 2020, 323(13): 1239-1242.
7. Diagnosis and Treatment Guideline for COVID-19 (Trial 8th Edition) [J]. Chin J Viral Dis, 2020,10(05):321-328.
8. Hung IF, Lung KC, Tso EY, et al. Triple combination of interferon beta-1b, lopinavir-ritonavir, and ribavirin in the treatment of patients admitted to hospital with COVID-19: an open-label, randomised, phase 2 trial[J]. Lancet, 2020,395(10238):1695-1704.
9. Shi N, Guo L, Liu B, et al. Efficacy and safety of Chinese herbal medicine versus Lopinavir-Ritonavir in adult patients with coronavirus disease 2019: A

non-randomized controlled trial[J]. Phytomedicine, 2021, 81:153367.

10. Xiao M, Tian J, Zhou Y, et al. Efficacy of Huoxiang Zhengqi dropping pills and Lianhua Qingwen granules in treatment of COVID-19: A randomized controlled trial[J]. Pharmacol Res, 2020, 161:105126.
11. Moher D, Liberati A, Tetzlaff J, et al. Preferred reporting items for systematic reviews and meta-analyses: the PRISMA statement[J]. Ann Intern Med, 2009,151(4):264-W64.
12. Higgins JPT, Green S. Cochrane Reviewers' Handbook 5.3.0 [updated March 2014], Review Manager (RevMan) [Computer program]. Version 5.3.0. Available from www.cochrane-handbook.org
13. Panahi Y, Hosseini MS, Khalili N, et al. Antioxidant and anti-inflammatory effects of curcuminoid-piperine combination in subjects with metabolic syndrome: A randomized controlled trial and an updated meta-analysis[J]. Clin Nutr, 2015,34(6):1101-1108.
14. Liu M, Gao Y, Yuan Y, et al. Efficacy and safety of integrated traditional Chinese and western medicine for corona virus disease 2019 (COVID-19): a systematic review and meta-analysis[J]. Pharmacol Res, 2020, 158:104896.
15. Duan C, Xia GW, Zheng CJ, et al. Clinical observation on Jinhua Qinggan granule combined with conventional western medicine therapy in treating mild cases of coronavirus disease 2019 [J]. J Tradit Chin Med,2020,61(17):1473-1477.
16. Fu XX, Lin LP, Tan XX. Clinical observation on effect of Toujie Quwen granules in treatment of COVID-19 [J]. Chin J Exp Tradit Med Formul,2020,26(12):44-48.
17. Fu XX, Lin LP, Tan XX. Clinical study on 37 case of COVID- 19 treated with integrated traditional Chinese and western medicine [J]. Tradit Chin Drug Res Clin Pharmacol,2020,31(05):600-604.
18. Hu F, Guo AH, Huang L, et al. Multi-center clinical observation of Jinyinhua oral liquid combined with western medicine in treatment of moderate COVID-19 [J/OL]. J Tradit Chin Med:1-6[2020-11-4]. http://kns.cnki.net/kcms/detail/11.2166.R.20200819.0837.002.html.
19. Hu K, Guan WJ, Bi Y, et al. Efficacy and safety of Lianhuaqingwen capsules, a

repurposed Chinese herb, in patients with coronavirus disease 2019: A multicenter, prospective, randomized controlled trial [J/OL]. Phytomedicine: 153242[2020-05-16]. https://pubmed.ncbi.nlm.nih.gov/32425361/
20. Qiu M, Li Q T, Zhu DP, et al. Efficacy observation of Maxing Xuanfei Jiedu decoction on moderate COVID-19 patients[J]. J Emerg Tradit Chin Med,2020,29(07):1129-1130+1132.
21. Sun HM, Xu F, Zhang L, et al. Study on clinical efficacy of Lianhua Qingke granule in treatment of mild and ordinary COVID-19 [J]. Chin J Exp Tradit Med Formul,2020,26(14):29-34.
22. Yang MB, Dang SS, Haung S, et al. Multi-center clinical observation of Reyanning mixture in treatment of COVID-19 [J]. Chin J Exp Tradit Med Formul,2020,26(14):7-12.
23. Yu P, Li YZ, Wan SB, et al. Clinical efficacy of Lianhua Qingwen granule combined with abidol in treating coronavirus disease 2019 [J]. Chin Pharm J,2020,55(12):1042-1045.
24. Zhang CT, Yang Y, You FM, et al. Clinical study on COVID-19 from the perspective of "Yidujiashi" theory [J]. Pharmacol Clin Chin Materia Medica,2020,36(02):43-45.
25. Zhang YL, Lei L, Xu Y, et al. Clinical efficacy of Jinyinhua oral liquid in the treatment of 80 patients with coronavirus disease 2019 [J]. Chin Pharm,2020,29(09):23-26.
26. Zhou WM, Zhao FM, Li BL, et al. Clinical value of diammonium glycyrrhizinate in treatment of COVID-19 [J]. Chin J Virol,2020,36(02):160-164.
27. Professional Committee of infection and inflammation radiology of Chinese Research Hospital Association. Guidelines for Imaging Assisted Diagnosis of COVID-19 [J]. Chin J Med Imaging Technol,2020, 36(3):1-11.
28. Ministry of Health of the People's Republic of China. Guiding Principles for Clinical Research of New Chinese Materia Medica [M]. Beijing: China Medical Science and Technology Press, 2002:186.
29. Chen N, Zhou M, Dong X, et al. Epidemiological and clinical characteristics of

99 cases of 2019 novel coronavirus pneumonia in Wuhan, China: a descriptive study [J]. Lancet, 2020,395(10223):507-513.

30. Wang DW, Hu B, Hu C, et al. Clinical characteristics of 138 hospitalized patients with 2019 novel coronavirus-infected pneumonia in Wuhan, China [J]. JAMA, 2020, 323(11): 1061-1069.
31. Xiong X, Wang P, Su K, et al. Chinese herbal medicine for coronavirus disease 2019: A systematic review and meta-analysis[J]. Pharmacol Res, 2020, 160:105056.
32. Zeng M, Li L, Wu Z. Traditional Chinese medicine Lianhua Qingwen treating corona virus disease 2019(COVID-19): Meta-analysis of randomized controlled trials[J]. PLoS One, 2020, 15(9): e0238828.
33. Ang L, Song E, Lee HW, et al. Herbal medicine for the treatment of coronavirus disease 2019 (COVID-19): A systematic review and meta-analysis of randomized controlled trials[J]. J Clin Med, 2020,9(5):1583.
34. Yang J. Warm Diseases [M]. Beijing: People's Health Publishing House, 2006.
35. Gao D, Niu M, Wei SZ, et al. Identification of a pharmacological biomarker for the bioassay-based quality control of a thirteen-component TCM formula (Lianhua Qingwen) used in treating influenza A virus (H1N1) infection[J]. Front Pharmacol, 2020,11:746.
36. Xia QD, Xun Y, Lu JL, et al. Network pharmacology and molecular docking analyses on Lianhua Qingwen capsule indicate Akt1 is a potential target to treat and prevent COVID-19[J]. Cell Prolif, 2020,53(12):e12949.
37. Ye M, Luo G, Ye D, et al. Network pharmacology, molecular docking integrated surface plasmon resonance technology reveals the mechanism of Toujie Quwen Granules against coronavirus disease 2019 pneumonia [J/OL]. Phytomedicine: 153401 [2020-10-28]. https://pubmed.ncbi.nlm.nih.gov/33191068/
38. Gao M, Li X, He L, et al. Diammonium glycyrrhizinate mitigates liver injury via inhibiting proliferation of NKT cells and promoting proliferation of tregs[J]. Drug Des Devel Ther,2019,13:3579-3589.
39. Zhou XN, Dong Q, Kan XZ, et al. Immunomodulatory activity of a novel

polysaccharide from Lonicera japonica immunosuppressed mice induced by cyclophosphamide [J]. PloS one,2018,13(10):1-15.

40. Bai XY, Chai YW, Shi WL, et al. Lonicera japonica poly-saccharides attenuate ovalbumin-induced allergic rhinitis by regulation of Th17 cells in BALB/c mice[J]. J Functional Foods,2020,65:103758.
41. Wang J, Hou L, Chen YQ, et al. Extraction and purification of the Lonicera japonica polysaccharide and its antiviral activity in vitro[J]. Chin J Hosp Pharm, 2018,38(8): 810-812.
42. Qu XY, Li QJ, Zhang HM, et al. Protective effects of phillyrin against influenza a virus in vivo[J]. Arch Pharm Res, 2016, 39(7):998-1005.29.
43. Jiang Q, Chen J, Long X, et al. Phillyrin protects mice from traumatic brain injury by inhibiting the inflammation of microglia via PPARγ signaling pathway[J]. Int Immunopharmacol, 2020,79:106083.
44. He W, Ma J, Chen Y, et al. Ephedrine hydrochloride protects mice from staphylococcus aureus-induced peritonitis[J]. Am J Transl Res, 2018,10(3):670-683.
45. Wei W, Du H, Shao C, et al. Screening of antiviral components of Ma Huang Tang and investigation on the ephedra alkaloids efficacy on influenza virus type A[J]. Front Pharmacol, 2019,10:961.
46. Fara A, Mitrev Z, Rosalia RA, et al. Cytokine storm and COVID-19: a chronicle of pro-inflammatory cytokines[J]. Open Biol,2020,10(9):200160.
47. Ragab D, Salah Eldin H, Taeimah M, et al. The COVID-19 cytokine storm; what we know so far[J]. Front Immunol, 2020,11:1446.

Supporting information

S1 Checklist. PRISMA 2009 checklist.

Response to Reviewers

Reviewer #1: kindly, find the primary review and comments in the attached Pdf file, in addition, please pay attention to the following points:
-The main claim of the paper is clear and significant, specially in such unprecedent situation.

-The analysis of data supports the claim of the paper, however; it would be better to connect this study with more previous published data and literatures in a way that reduce duplication and support the findings of this paper.
Response: in the discussion section, this review has linked this study with more previously published data and literature for analysis.
-a more detailed protocol of the statistical analysis is needed especially, most of the data used in the analysis has been retrieved from papers in Chinese language.
Response: in our review, a more detailed protocol of the statistical analysis was developed. Trials on Chinese herbal medicine for mild to moderate COVID-19 were conducted in mainland China. Most of the trials were published online in Chinese. Therefore, most of the data used in the analysis has been retrieved from papers in Chinese language.
-Type of samples in treatment and control groups doesn't exclude the possibility of synergistic/ combination effect between CHM and western medicine. have you had any studies that used CHM only on separate groups as a treatment? Was there any control group that didn't receive any treatment? is there any information about hospitalization or receiving any other special care(ex. ventilator) beside the treatment?
Response: trials of Chinese herbal medicine in the treatment of mild to moderate COVID-19 were included in this review. The treatment group was treated with Chinese herbal medicine combined with conventional therapy. No trials that used CHM only on separate groups as a treatment. There was no control group that did not receive any treatment. Since the participants were diagnosed as mild to moderate COVID-19, patients did not receive ventilator treatment. The specific treatment information is listed in Table 1.
i.e: we can't conclude for sure the CHM as a separate, effective, and safe treatment for mild to moderate COVID-19.

Response: the conclusion of this review is that Chinese herbal medicine combined with conventional therapy could be effective and safe in the treatment of adults with mild to moderate COVID-19.

Reviewer #2: Valuable data was provided in this manuscript, which are not easily assessible for international readers outside China. Hence, I have to stress that this manuscript presents precious and valuable data that will benefit the literature and improve understanding of the role of TCM in COVID-19. However, in general, I find that there is lack of clarity in definition of many things including outcome measures and treatment groups. Importantly, the discussion was superficial. There needs to be correlation between ROB, quality of study, heterogeneity and interpretation of results.

Please find my suggestion as below and as specify in the attachment:

1. Strongly suggest for professional language/ scientific proof-reading to correct grammar, sentence structuring, and selection of words that are preferred to represent precise scientific writing for the entire manuscript. Kindly check for the use of oxford comma and appropriate/excessive use of connective words throughout. The authors in particular like to start sentences with the word "And". Spacing between words and symbols needs to be checked and made consistent.

Response: grammar, sentence structure, comma, and connective words have been corrected.

2. The eligibility criteria can be rewritten as inclusion and exclusion criteria clearly; or rearrange with clearer subtopics differentiation. The different levels of the subtopics in the methods needs to be clear. For example (here I am using numbers to explain an example of how the different levels needs to be clarified. It is to the authors discretion on presenting this without the numbers)

Response: the eligibility criteria have been rewritten as inclusion and exclusion criteria.

3. Specific to the methods

a. Kindly check against the PRISMA checklist- Present full electronic search strategy for at least one database (please present the combination of keywords used); Describe method of data extraction from reports (e.g., piloted forms, independently, in duplicate) and any processes for obtaining and confirming data from investigators (kindly mention if attempts were made to seek for additional data)

Response: the PubMed search strategy is listed. The method of data extraction from reports, and any processes for obtaining and confirming data from investigators were described in this review.

b. Clarify inclusion criteria- oral Chinese herbal medicine only

Response: inclusion criteria have been clarified - oral Chinese herbal medicine only.

c. Outcome measures need to be well defined e.g. what is clinical cure rate, what is effective rate of lung CT

Response: Outcome measures (e.g. clinical cure rate, lung CT) have been well defined.

4. Results

a. arrange the level of subheadings accordingly as suggested for methods

Response: the level of subheadings has been arranged accordingly as suggested for methods.

b. definition of CHM and CWM needs to be clear- the naming of the groups. Although it is mentioned that CHM group received both herbal and western medicine in methods, CHM is still abbreviated as chinese herbal medicine. The results are mostly written as 'the outcomes are better with treatment by CHM', which can be confusing to interpret, and easily misunderstood as if CHM solely (without western medicine) is beneficial. Suggest to clearly describe what each group means with distinct abbreviations for groups. Perhaps it is also because of the choice of word 'by' which when read, is interpreted this way, hence consider rewriting the results section with more precise

selection of words.

Response: the naming of the groups has been rewritten.

5. Discussion

Although an interesting topic with very valuable data (I cannot emphasize this enough, this is very valuable data), the discussion is superficial and lacked depth. few suggestion of topics to discuss include

- heterogeneity of the studies and the impact on the findings.
- impact of different formulations used and how did the authors came to collectively interpreting them in the same meta-analyses (also consider that different herbs would have acted differently, and certainly herb-herb interaction should be discussed)
- risk of bias and how that affects results interpretation
- discuss on adverse events, reporting bias?
- quality of herbal intervention used
- suggest to consider consort checklist for tcm to evaluate quality of reporting which can further strengthen discussion
- how does this new information applies to the global scenario and what are the challenges of applying TCM in this scenario
- difference between TCM approach (Which is based on individualised assessment, and can be even affected by factors such as diet, body type, environment, geographical location, weather) and western medicine approach
- it is also important to point out that the concept of selecting treatment based on TCM philosophy is vastly different. My own personal experience consulting TCM experts from China , which I quote him, the treatment in China (Wuhan experiencing winter that time) may not suit for countries with different climate and weather (e.g. a Southeast Asian country with hot and humid climate, with different diet practices)
- also consider that herbs, in raw form, extracted, or in different extraction medium in phytochemistry context would yield different phytocompounds, and one of the main gap here is a lack of consistency/ documentation/ quantitation/ interpretation of what is the mechanisms and bioactive compound involved
- regulatory challenges
- contribution of confounding factors such as co-morbidities, differences in western medicine used

Response: in our review, the suggestions on the above topics have been incorporated into the discussion.

6. Conclusion

The conclusion partly answers the objective. However, critical appraisal (as mentioned in the discussion section) would help interpret the results better and make it more relevant to the global scenario. The limitations are not only to conducting high quality studies (to which quality of studies were not actually evaluated and discussed in the discussion section), but application to the world, and consideration of knowledge gap.

Response: critical appraisal has been made.

7. Is the western medicine arm treatment really identical? There is no data available on what is given as western medicine and difficult to decide if they are identical, similar, or if they actually can be a confounding factor.

Response: the western medicine arm treatment really is not identical in different trials. Specific treatment information is listed in Table 1.

8. It would be good to at least describe what are the different composition of the common TCM formulations used.

Response: the different components of TCM were described in this review.

But overall, I am very appreciative that this data will be made available and I look forward to the amended version. Again, I cannot emphasize enough how valuable these data are.

Reviewer #3: Reviewer's Comments

Chinese herbal medicine in adults with mild to moderate coronavirus disease 2019(COVID-19): A systematic review and meta-analysis with MS ID PONE-D-20-38124.

Major Comments

1. Meta-analytical studies have been carried out majorly on the basis of ref 10-20 and all of them are published in Chinese journals except ref 14 only, which indicates towards the biasness of choice of content used for carrying out the study. Authors are recommended to refer the content from other sources as well to further validate the findings.

Response: trials of Chinese herbal medicine in the treatment of mild to moderate COVID-19 were comprehensively searched in eight electronic databases. Potentially eligible data was obtained by manually searching the reference list of previously published reviews. If possible, the conference abstracts were reviewed to find unpublished trials, and the data was obtained by contacting the author.

2. COVID-19 data provided in introduction section is contradictory with WHO data. Authors are suggested to cross-check the COVID-19 count provided on WHO website.

Response: COVID-19 data was cross-checked according to WHO website.

3. Conclusion of study is not in accordance with results therefore needs to be modified accordingly.

Response: conclusion of our study was modified in accordance with results.

4. Manuscript mandatorily needs to be handled by language experts as there exists several ambiguities in its current form.

Response: our manuscript was handled by language experts.

Minor Comments

1. Abbreviations are missing throughout the manuscript.

Response: abbreviations full names were listed in the manuscript.

2. Cross-check the format of references to maintain homogeneity.

Response: the format of references was cross-checked.

Reviewer #4: This is a very important review to publish at this time. These findings are very relevant and contribute to the essential knowledge about a globally crippling disease. The review was performed with rigorous standards and therefore the results can contribute significantly to the prevention and treatment of COVID-19. Thank you for your work.
