# Supplementary material for: Add-on effect of Chinese herbal medicine in the treatment of mild to moderate COVID-19: A systematic review and meta-analysis
Source: PLoS One. 2021 Aug 20;16(8):e0256429. doi: 10.1371/journal.pone.0256429 (PMC8378756; doi:10.1371/journal.pone.0256429)
Supplement: Attachment — Submitted filename: Response to Reviewers.pdf [file pone.0256429.s009.pdf]

Response to Reviewers

Reviewer #2： Thank you for revising your very valuable manuscript. It was really interesting and my honour to have read such valuable data. It is really precious. However, major revisions especially in data interpretation, critical analysis and discussion writing, as well as definition of outcomes still needs to be amended.

1) Introduction - need to better justify the need for this systematic review (i.e research gap leading to the objective). It is too brief. In fact the introduction in abstract better justified the purpose of the review than the introduction in the manuscript. Suggest to amend. Also, very importantly, there is an overstatement on the findings when citing two studies on benefits of CHM in the introduction, please review again as commented in the manuscript).

Response: the need for this systematic review has been further illustrated. In the introduction, the authors have stated the specific benefits of CHM combined with conventional therapy in the treatment of mild to moderate COVID-19.

2) Methods and results: Please define in depth for outcomes: lung CT- what is the definition of improved/good lung CT etc, please elaborate. and how does that relate to outcome assessment. The same applies to definition of clinical cure rate and viral nucleic acid testing. The definition of effective, improved, ineffective also needs to be clarified

Response: the authors have defined in depth for outcomes of lung CT, clinical cure rate, and viral nucleic acid testing.

3) Results- table 2 is truncated and I could not view the entire table. The resolution of the forest plots were low and many are not readable. I suggest to rename the axis to favour therapy alone and favour CHM + conventional therapy instead of favour control/favour experimental to make it easier to read at first glance.

Response: the authors have modified table 2 and the resolution of the forest plots. The authors have renamed the axis to favour conventional therapy and favour combination therapy instead of favour control/favour experimental to make it easier to read at first glance.

4) The benefits on inflammatory markers- this is very important to be clarified. Is higher levels better or lower levels better? This would depend on the course of the disease. For example, at initial stages, we probably need higher levels of immunity to produce antiviral effects. However, if inflammation is overt and prolonged at later stages, this may push the patient into a cytokine storm and hence detrimental i.e. timing is important. This component is not discussed and should be discussed, considering that inflammatory markers is one of the outcomes assessed and duration of administration varies.

Response: the benefits on inflammatory markers have been clarified. Subgroup analyses of outcomes of inflammatory markers were carried out according to treatment duration.

5) I would appreciate more discussion on CHM+ conventional therapy discussion in view that this plays a major role in your findings too

Response: this review has discussed combination therapy of CHM and conventional therapy.

6) English has improved but still needs to be improved further. In particular in selection of accurate and politically appropriate words, and an entire highlighted paragraph in discussion. Minor formatting changes of the tables are also recommended.

Response: the authors have received linguistic assistance provided by AJE (https://secure.aje.com/cn/researcher/) during the preparation of this manuscript. The format of the tables has been modified.

7) Data availability statements should be clarify- its contradicting with what is already provided.

Response: the authors have clarified the data availability statements.
