# Supplementary material for: Add-on effect of Chinese herbal medicine in the treatment of mild to moderate COVID-19: A systematic review and meta-analysis
Source: PLoS One. 2021 Aug 20;16(8):e0256429. doi: 10.1371/journal.pone.0256429 (PMC8378756; doi:10.1371/journal.pone.0256429)
Supplement: Attachment — Submitted filename: Reviewers Comments (1).docx [file pone.0256429.s004.docx]

**Reviewer’s Comments**

Chinese herbal medicine in adults with mild to moderate coronavirus disease 2019(COVID-19): A systematic review and meta-analysis with MS ID PONE-D-20-38124.

**Major Comments**

1. Meta-analytical studies have been carried out majorly on the basis of ref 10-20 and all of them are published in Chinese journals except ref 14 only, which indicates towards the biasness of choice of content used for carrying out the study. Authors are recommended to refer the content from other sources as well to further validate the findings.
2. COVID-19 data provided in introduction section is contradictory with WHO data. Authors are suggested to cross-check the COVID-19 count provided on WHO website.
3. Conclusion of study is not in accordance with results therefore needs to be modified accordingly.
4. Manuscript mandatorily needs to be handled by language experts as there exists several ambiguities in its current form.

**Minor Comments**

1. Abbreviations are missing throughout the manuscript.
2. Cross-check the format of references to maintain homogeneity.
